# Supplementary material for: Antifungal Thiazolidines: Synthesis and Biological Evaluation of Mycosidine Congeners
Source: Pharmaceuticals (Basel). 2022 May 1;15(5):563. doi: 10.3390/ph15050563 (PMC9145892; doi:10.3390/ph15050563)
Supplement: Supplementary file 1 [file pharmaceuticals-15-00563-s001.zip › Supplement_1D_3-17.pdf]

3

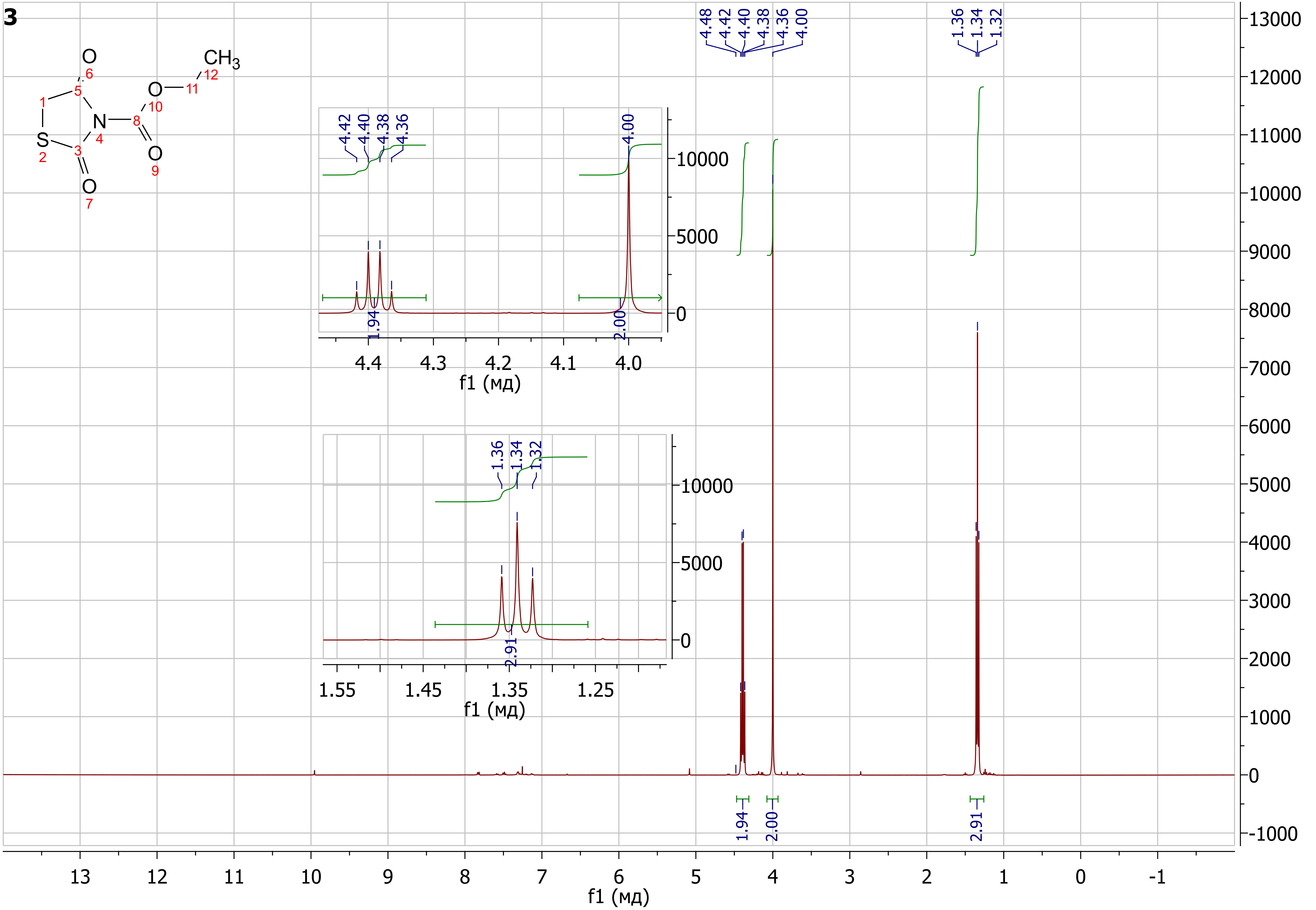

O
CH3
6
12
1
5
O
11
10
N
8
4
S
3
O
2
9
O
7
4.48
4.42
4.40
4.38
4.36
4.00
1.36
1.34
1.32
1.94
2.00
2.91
10000
5000
0
4.4
4.3
4.2
4.1
4.0
f1 (мд)
1.55
1.45
1.35
1.25
13000
12000
11000
9000
8000
7000
6000
4000
3000
2000
1000
-1000
13
12
11
10
9
7
6
3
2
1
-1

3

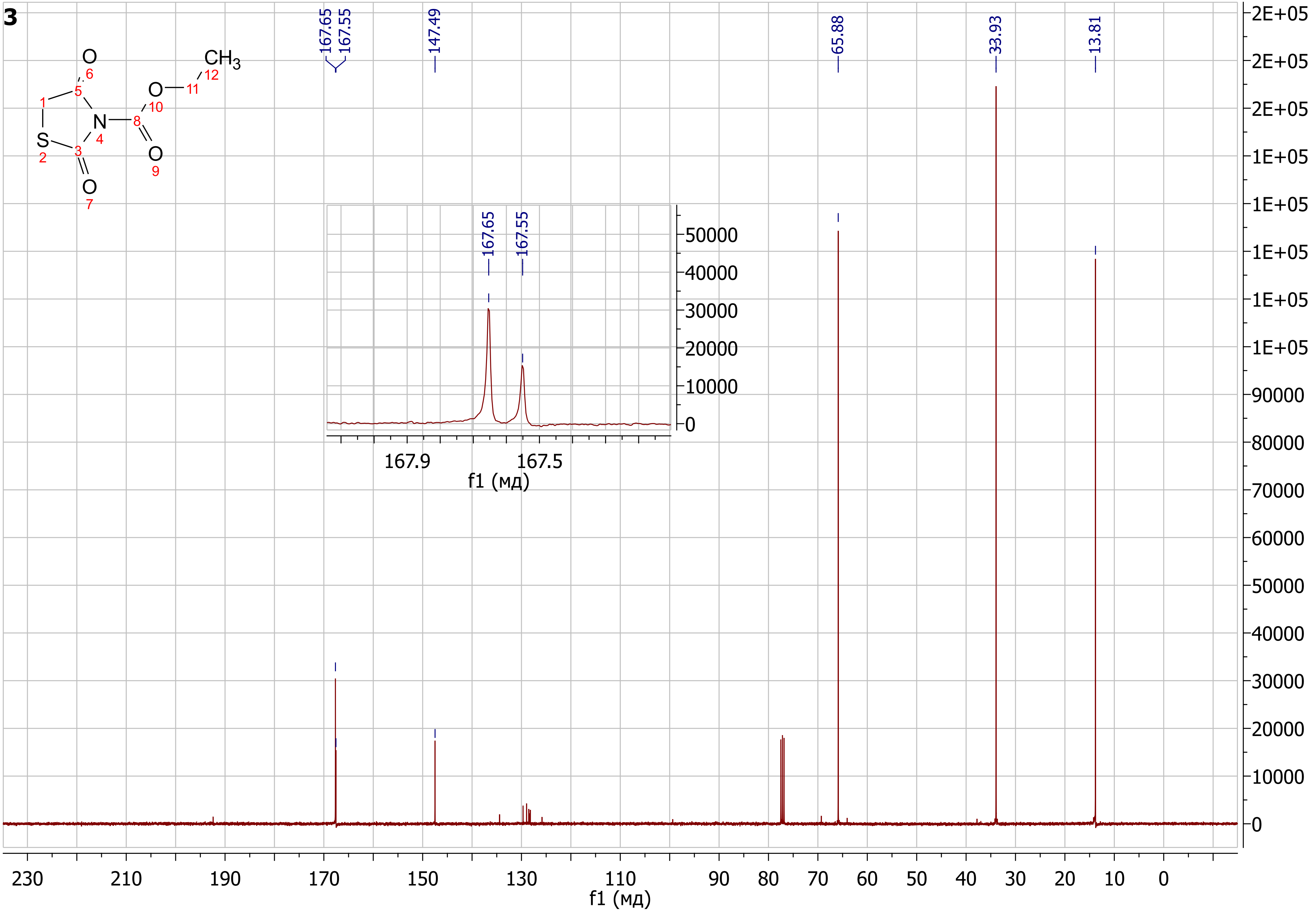

O
6
5
1
CH3
12
O
11
10
N
8
4
S
3
2
O
9
O
7
167.65
167.55
147.49
65.88
33.93
13.81
2E+05
1E+05
90000
80000
70000
60000
50000
40000
30000
20000
10000
0
50000
40000
30000
20000
10000
0
167.9
167.5
f1 (мд)
230
210
190
170
150
130
110
90
80
70
60
50
40
30
20
10
0
f1 (мд)

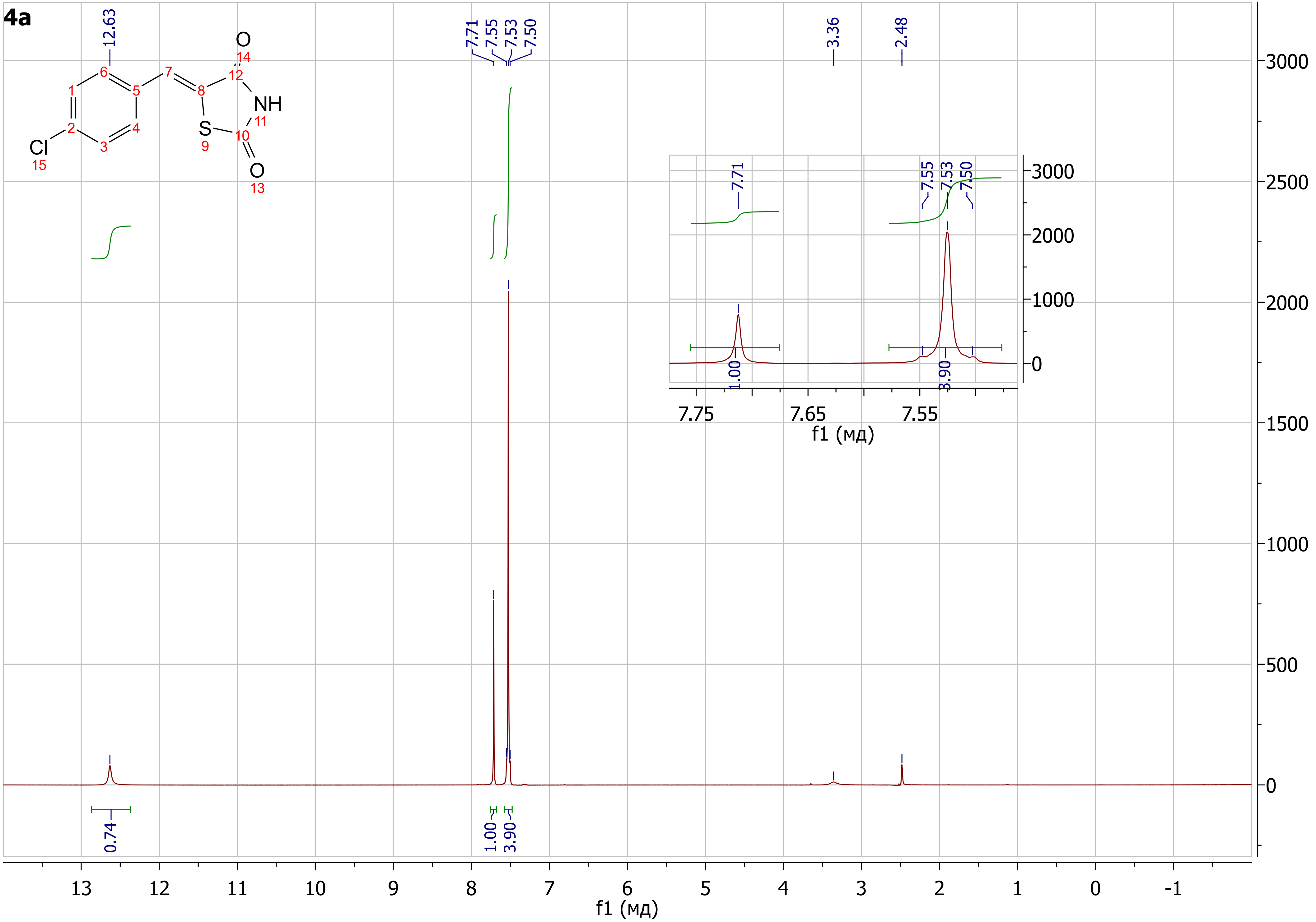

4a
Cl
NH
S
O
12.63
7.71
7.55
7.53
7.50
3.36
2.48
0.74
1.00
3.90
f1 (мд)

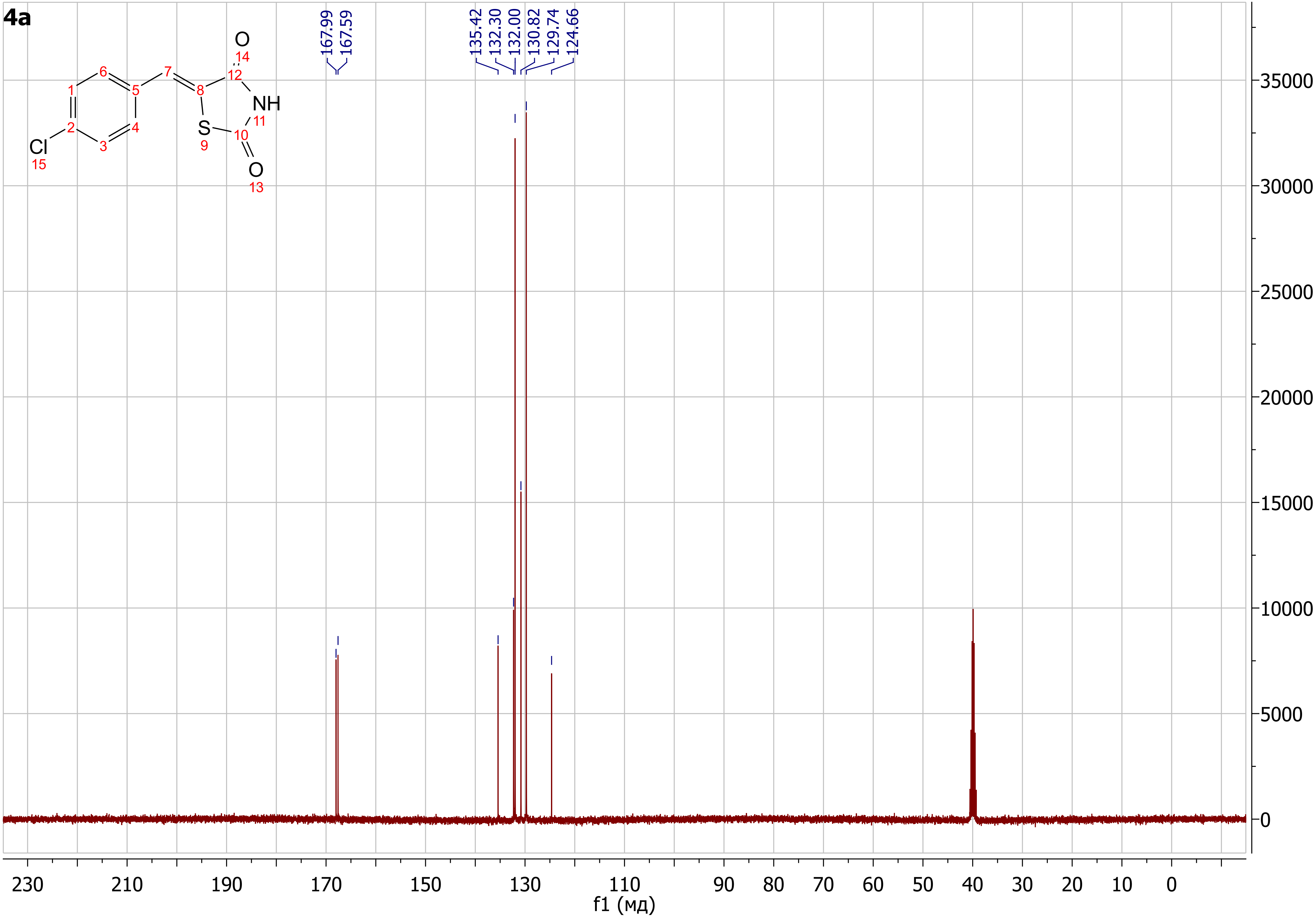

4a
Cl
NH
S
O
O
167.99
167.59
135.42
132.30
132.00
130.82
129.74
124.66
f1 (мд)

4a

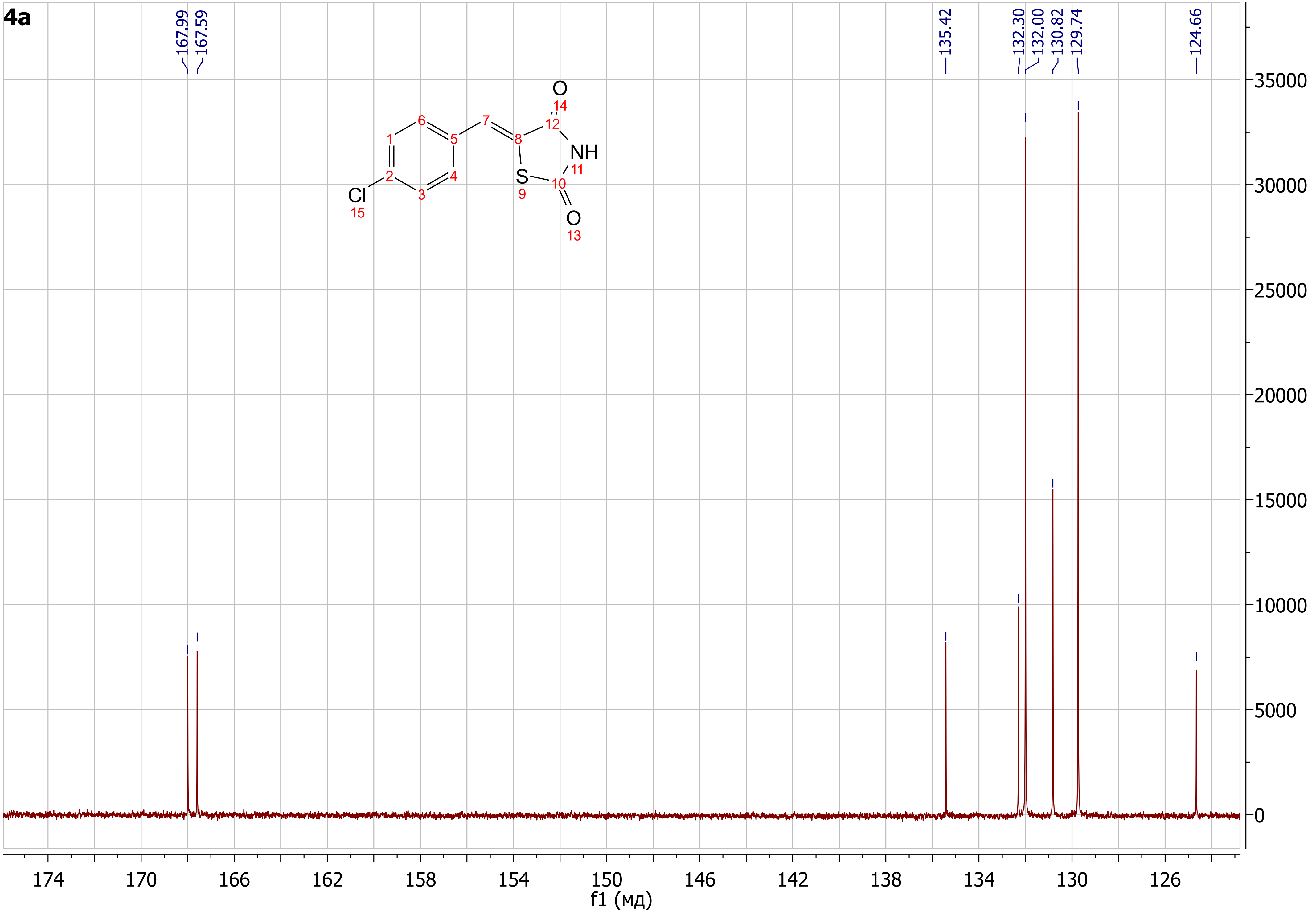

167.99
167.59
135.42
132.30
132.00
130.82
129.74
124.66
Cl
S
NH
O
O
35000
30000
25000
20000
15000
10000
5000
0
174
170
166
162
158
154
150
146
142
138
134
130
126
f1 (мд)

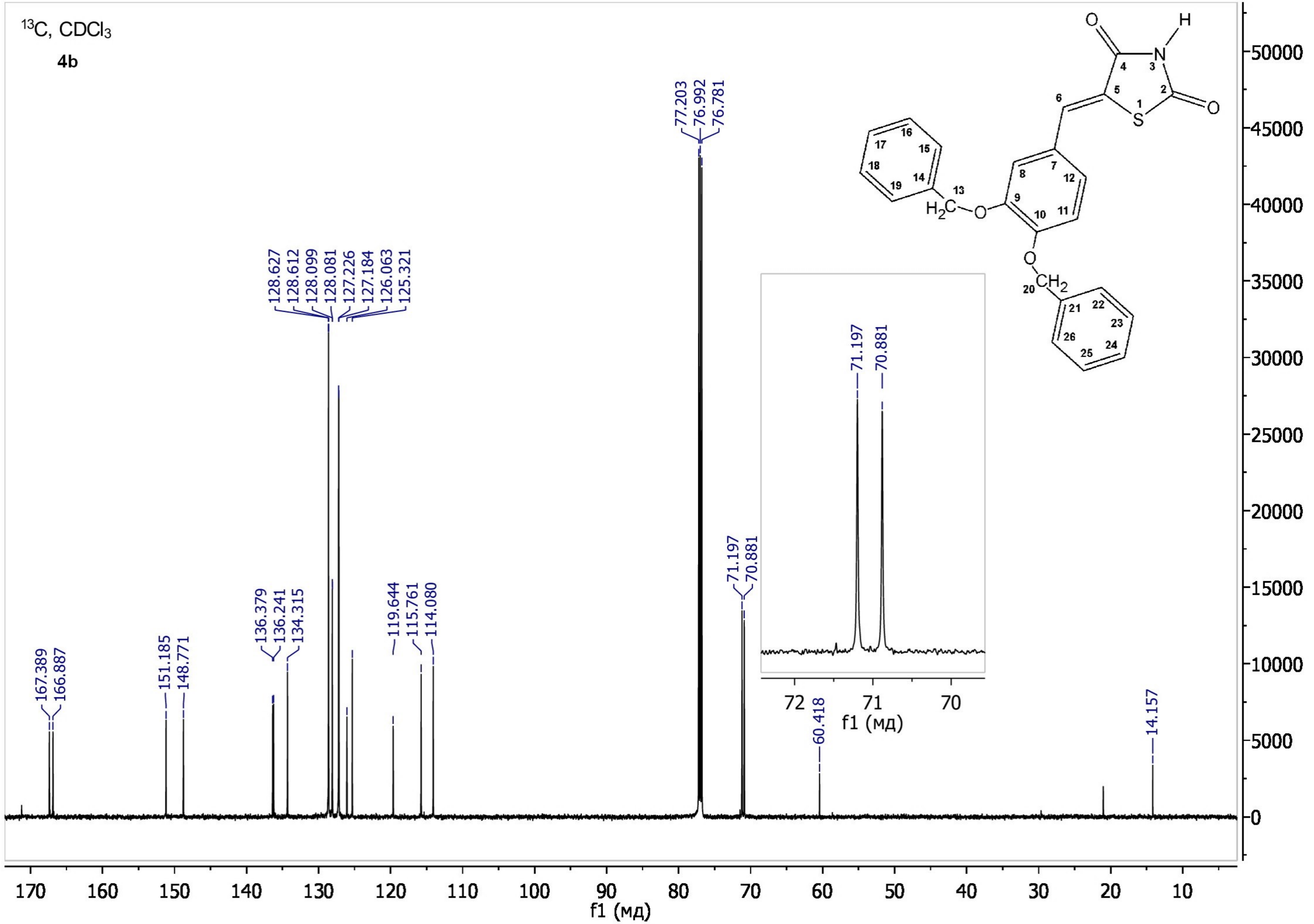

13C, CDCl3
4b
167.389
166.887
151.185
148.771
136.379
136.241
134.315
128.627
128.612
128.099
128.081
127.226
127.184
126.063
125.321
119.644
115.761
114.080
77.203
76.992
76.781
71.197
70.881
60.418
14.157
f1 (мд)

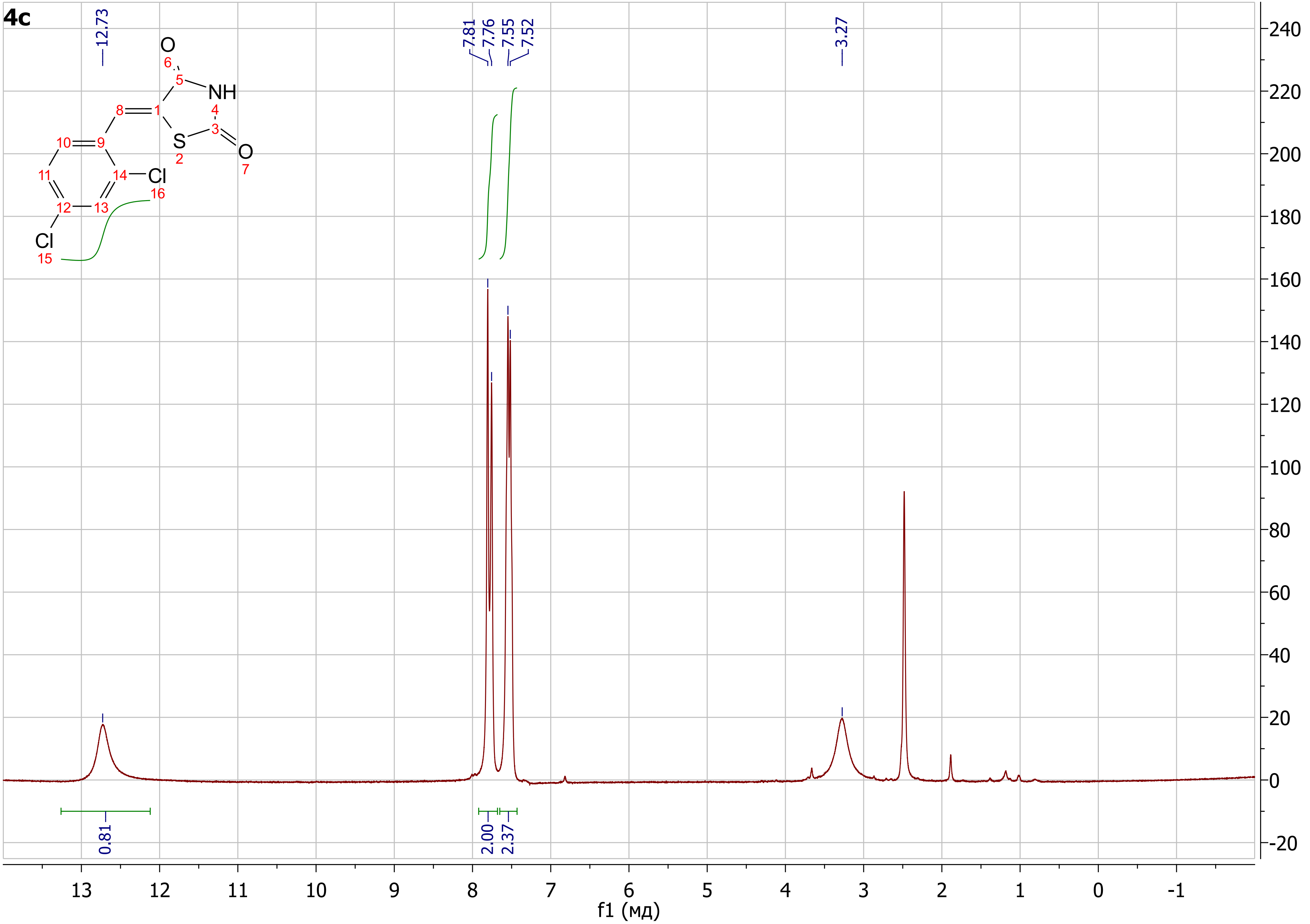

4c
12.73
7.81
7.76
7.55
7.52
3.27
O
6
5
NH
4
8
1
3
S
2
O
7
10
9
11
14
Cl
16
12
13
Cl
15
0.81
2.00
2.37
f1 (мд)

4c

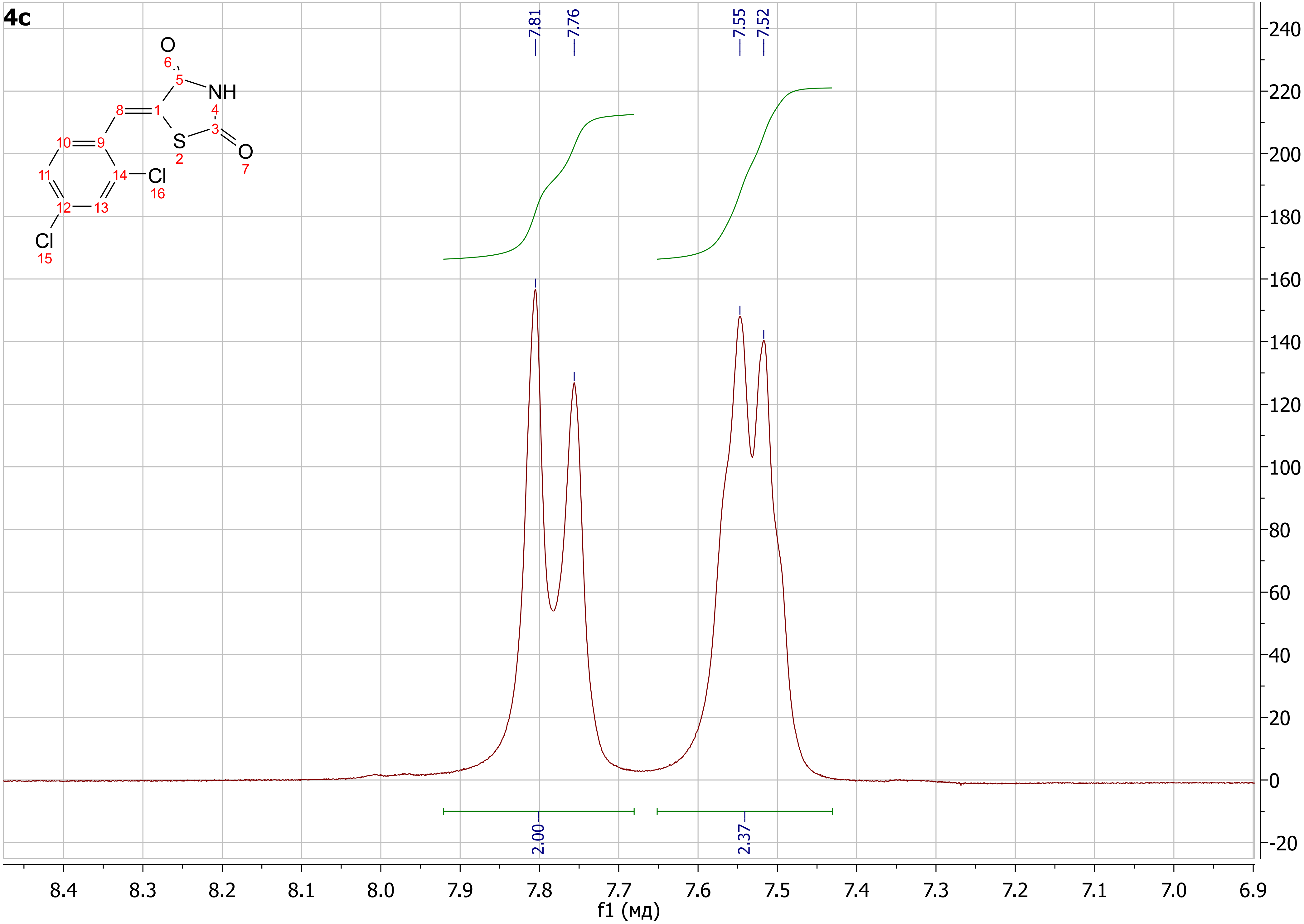

4c

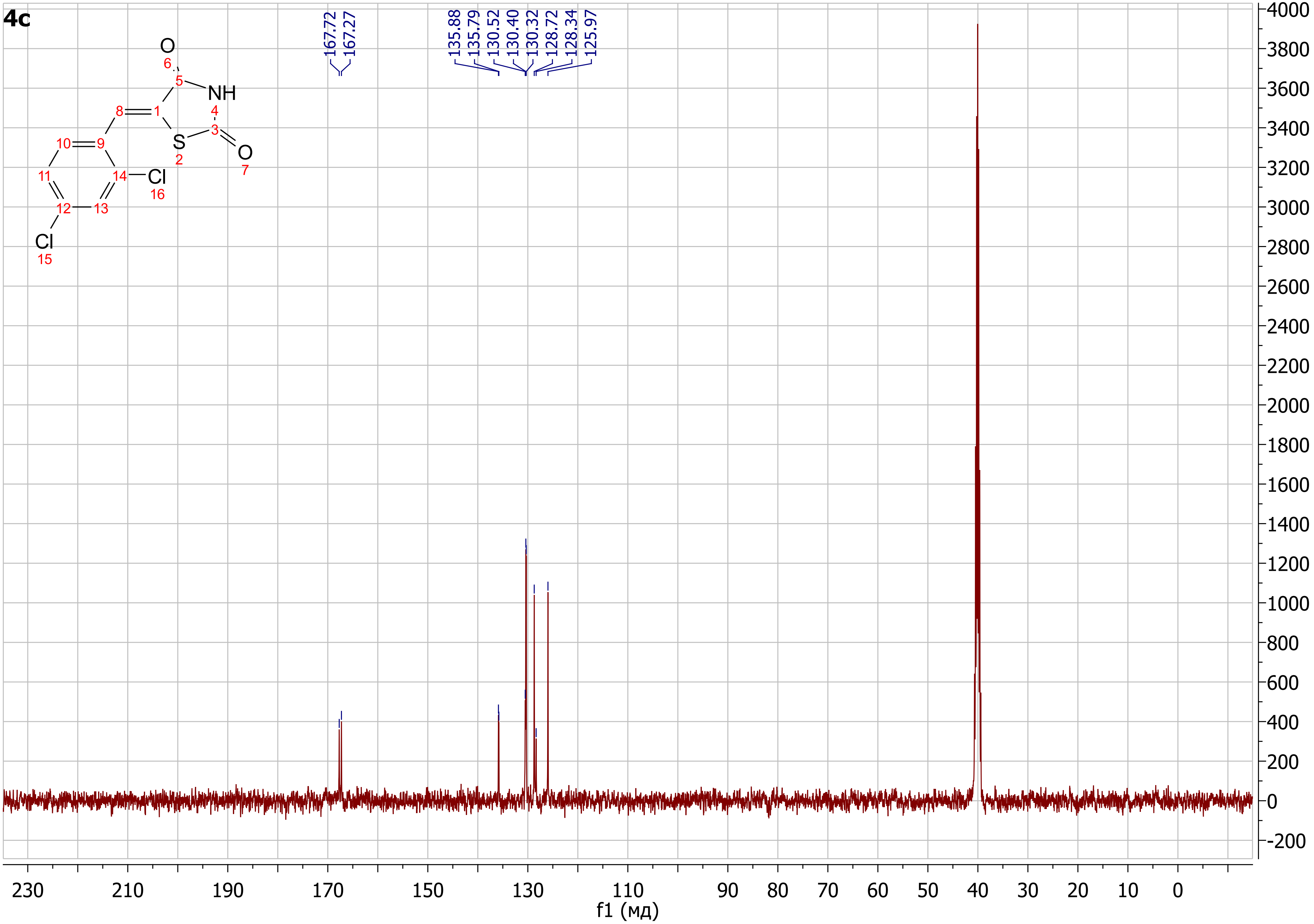

167.72
167.27
135.88
135.79
130.52
130.40
130.32
128.72
128.34
125.97
O
6
5
NH
4
8
1
3
10
9
S
2
O
7
11
14
Cl
16
12
13
Cl
15
f1 (мд)

4c

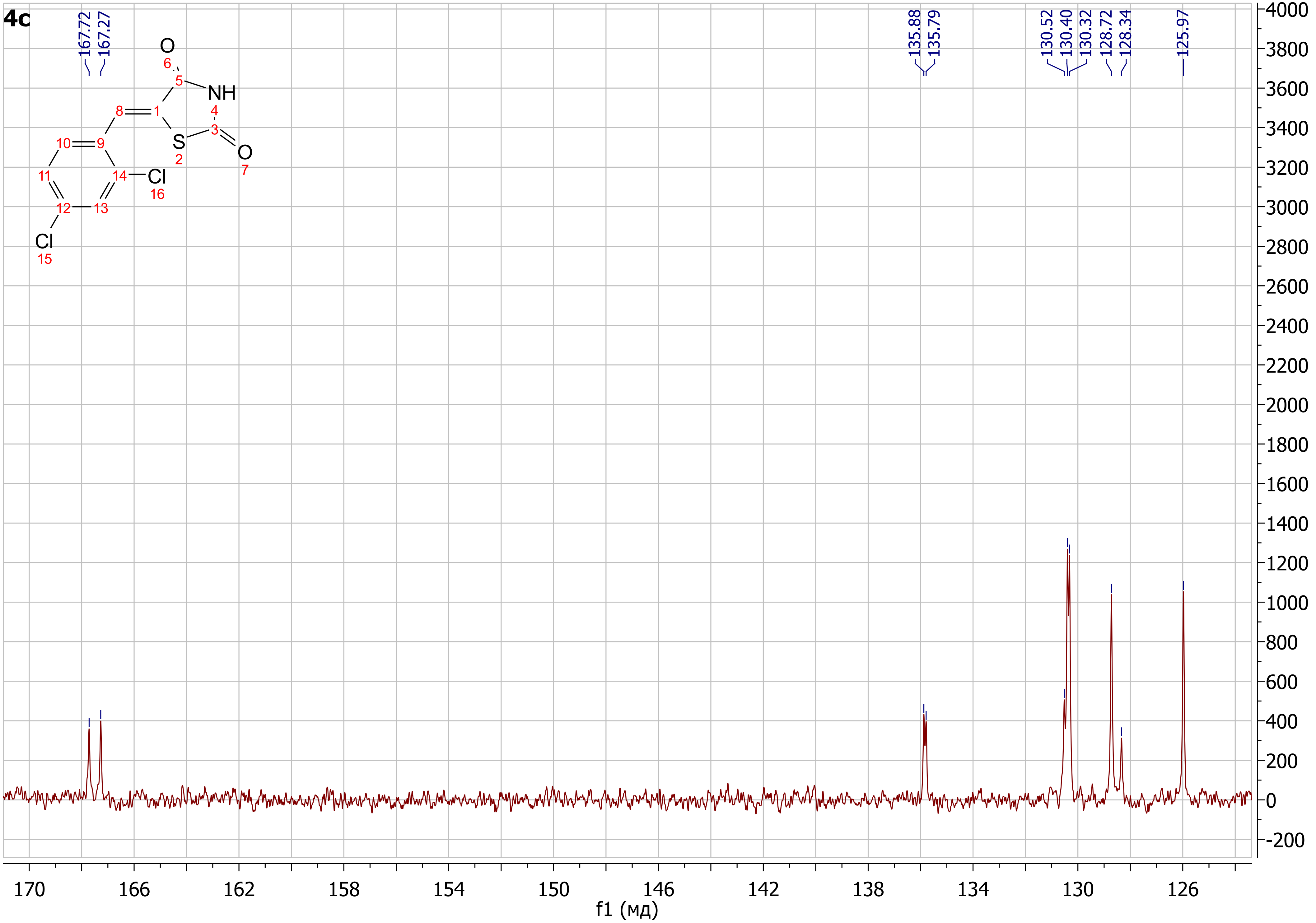

167.72
167.27
135.88
135.79
130.52
130.40
130.32
128.72
128.34
125.97
O
6
5
NH
4
8
1
3
S
2
O
7
10
9
11
14
Cl
16
12
13
Cl
15
4000
3800
3600
3400
3200
3000
2800
2600
2400
2200
2000
1800
1600
1400
1200
1000
800
600
400
200
0
-200
170
166
162
158
154
150
146
142
138
134
130
126
f1 (мд)

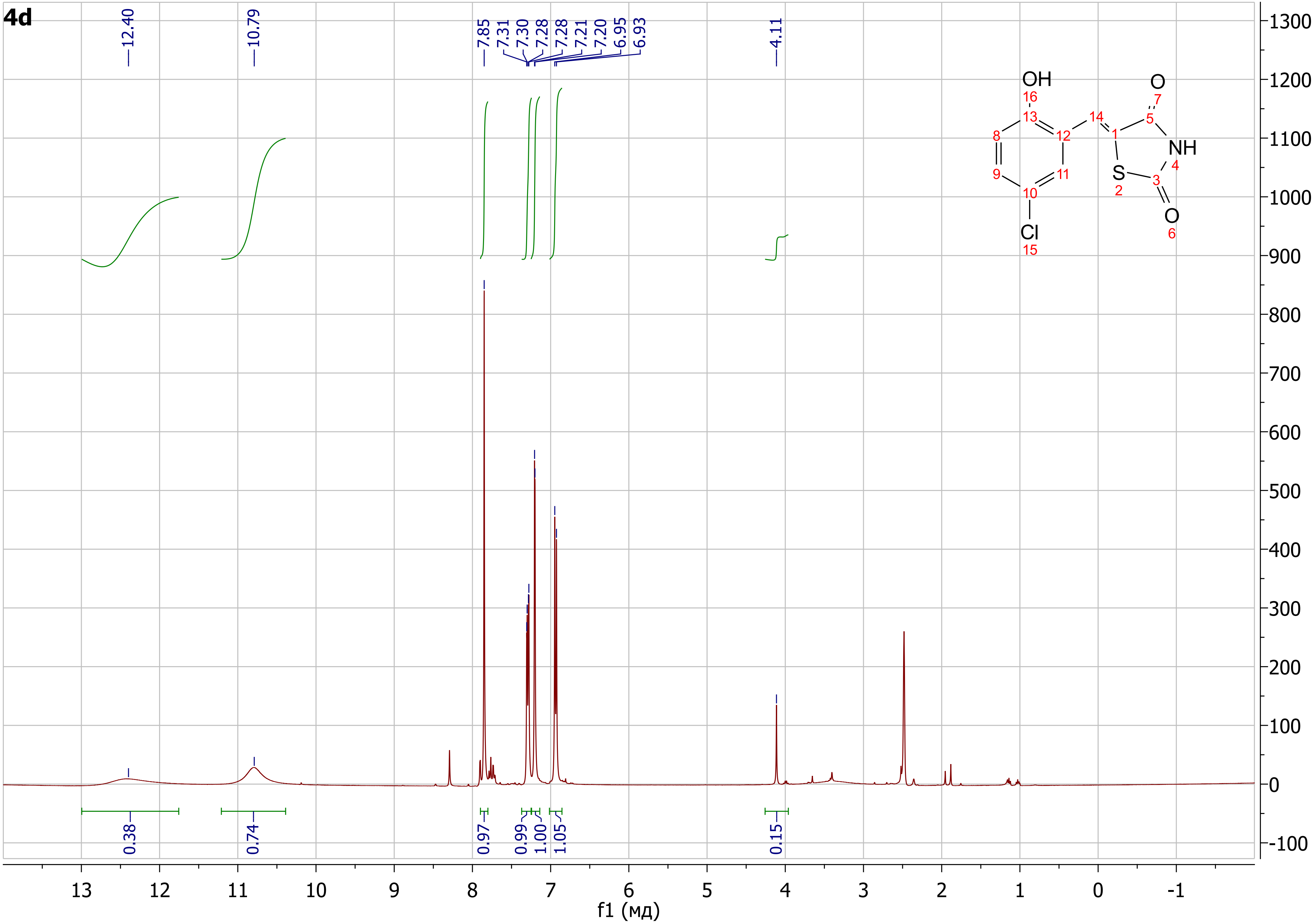

4d
12.40
10.79
7.85
7.31
7.30
7.28
7.28
7.21
7.20
6.95
6.93
4.11
OH
O
NH
S
Cl
O
0.38
0.74
0.97
0.99
1.00
1.05
0.15
f1 (мд)

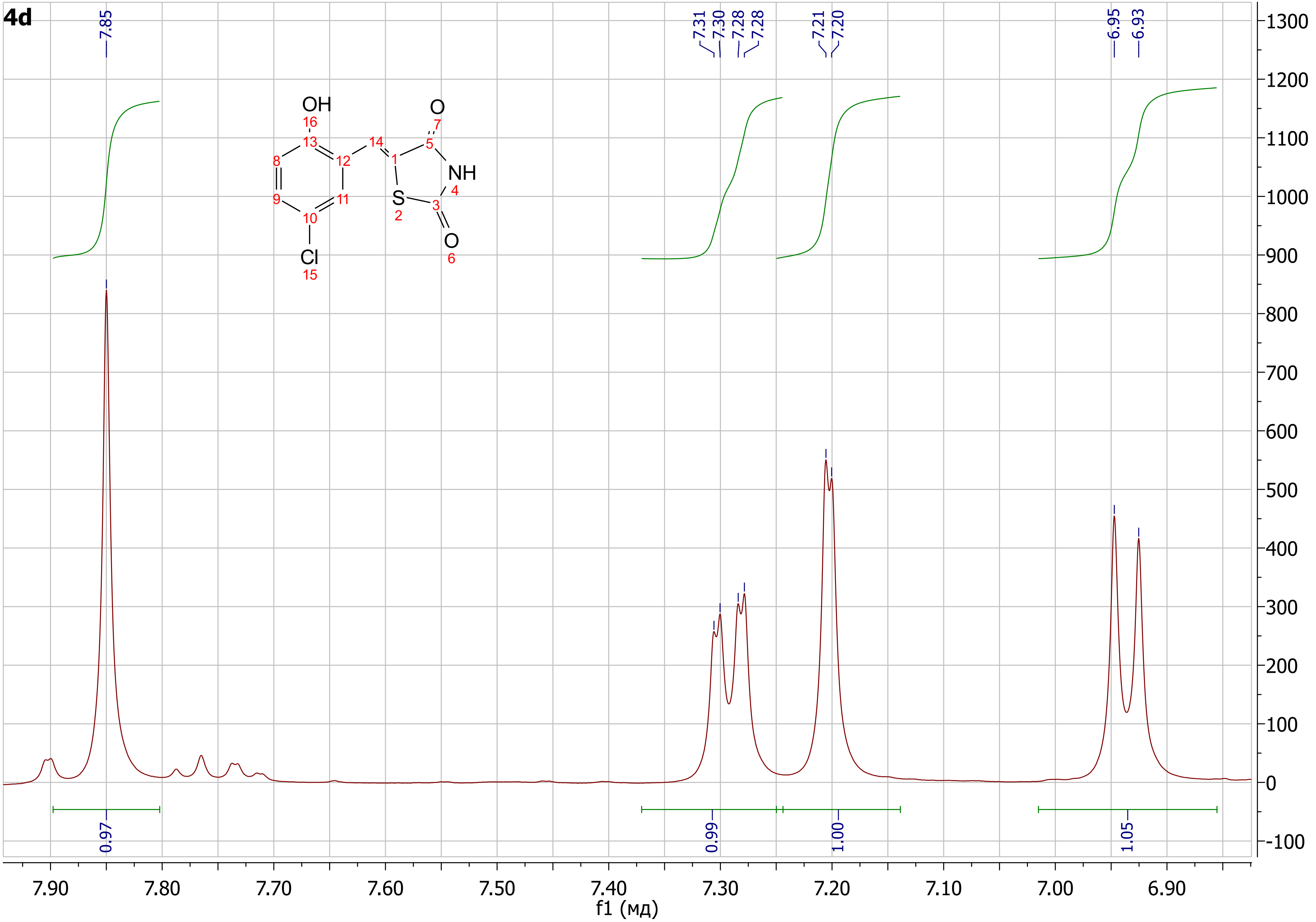

4d
7.85
7.31
7.30
7.28
7.28
7.21
7.20
6.95
6.93
OH
16
13
12
14
1
5
7
O
NH
4
8
9
11
10
S
2
3
O
6
Cl
15
0.97
0.99
1.00
1.05
7.90
7.80
7.70
7.60
7.50
7.40
7.30
7.20
7.10
7.00
6.90
f1 (мд)
1300
1200
1100
1000
900
800
700
600
500
400
300
200
100
0
-100

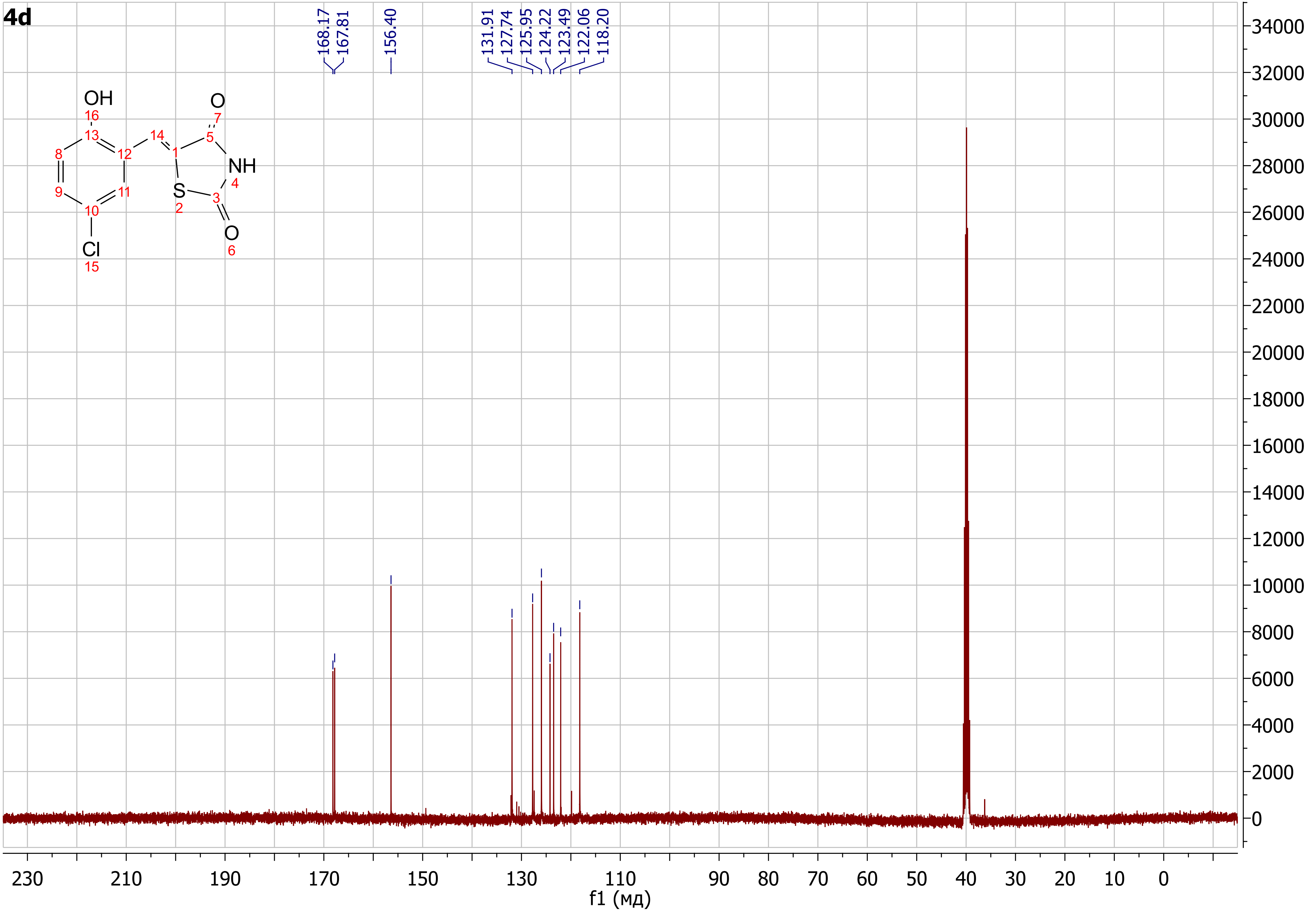

4d
168.17
167.81
156.40
131.91
127.74
125.95
124.22
123.49
122.06
118.20
OH
16
13
8
12
14
1
5
7
O
NH
4
9
11
10
S
2
3
O
6
Cl
15
f1 (мд)

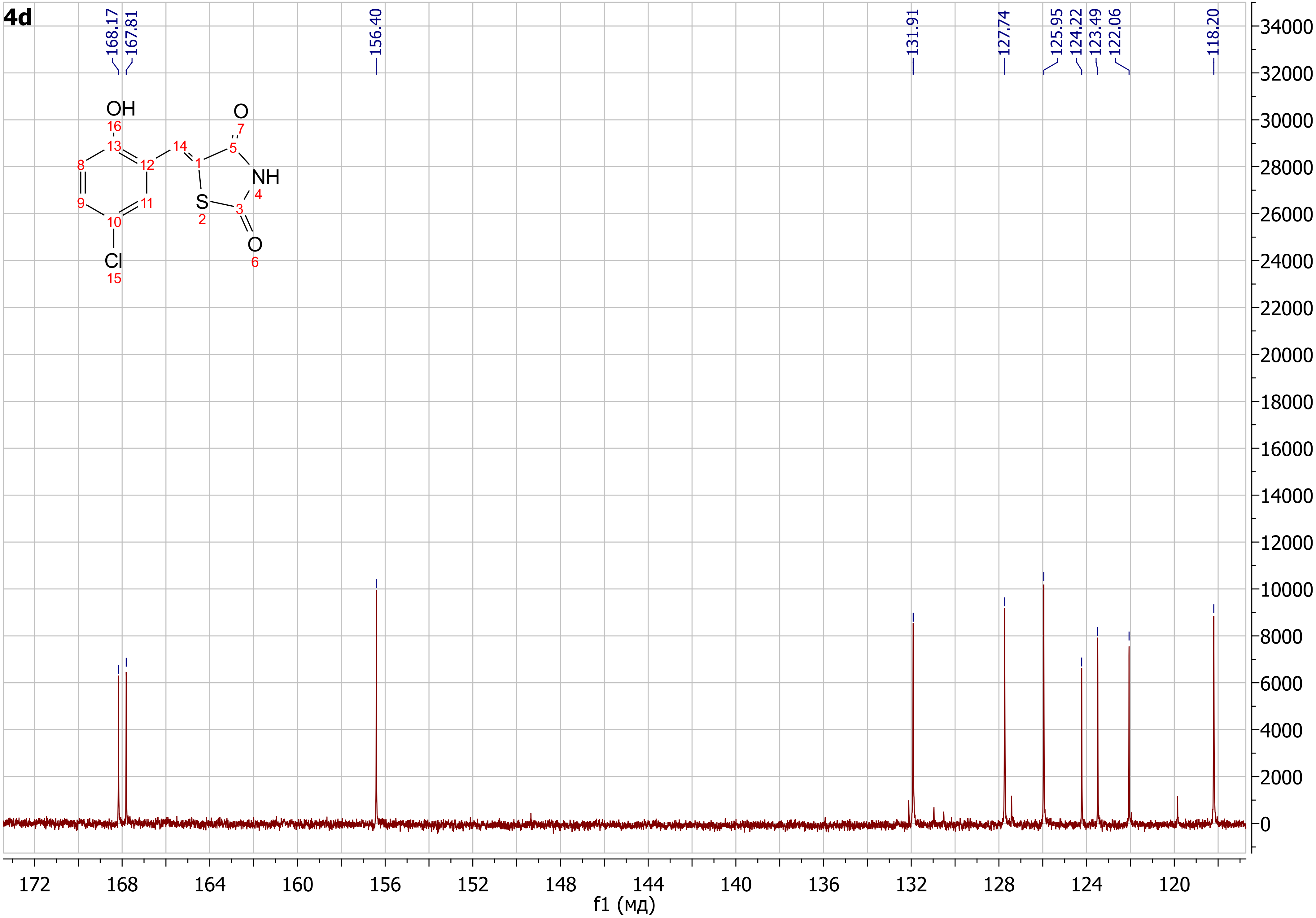

4d
168.17
167.81
156.40
131.91
127.74
125.95
124.22
123.49
122.06
118.20
OH
O
NH
S
O
Cl
172
168
164
160
156
152
148
144
140
136
132
128
124
120
f1 (мд)
34000
32000
30000
28000
26000
24000
22000
20000
18000
16000
14000
12000
10000
8000
6000
4000
2000
0

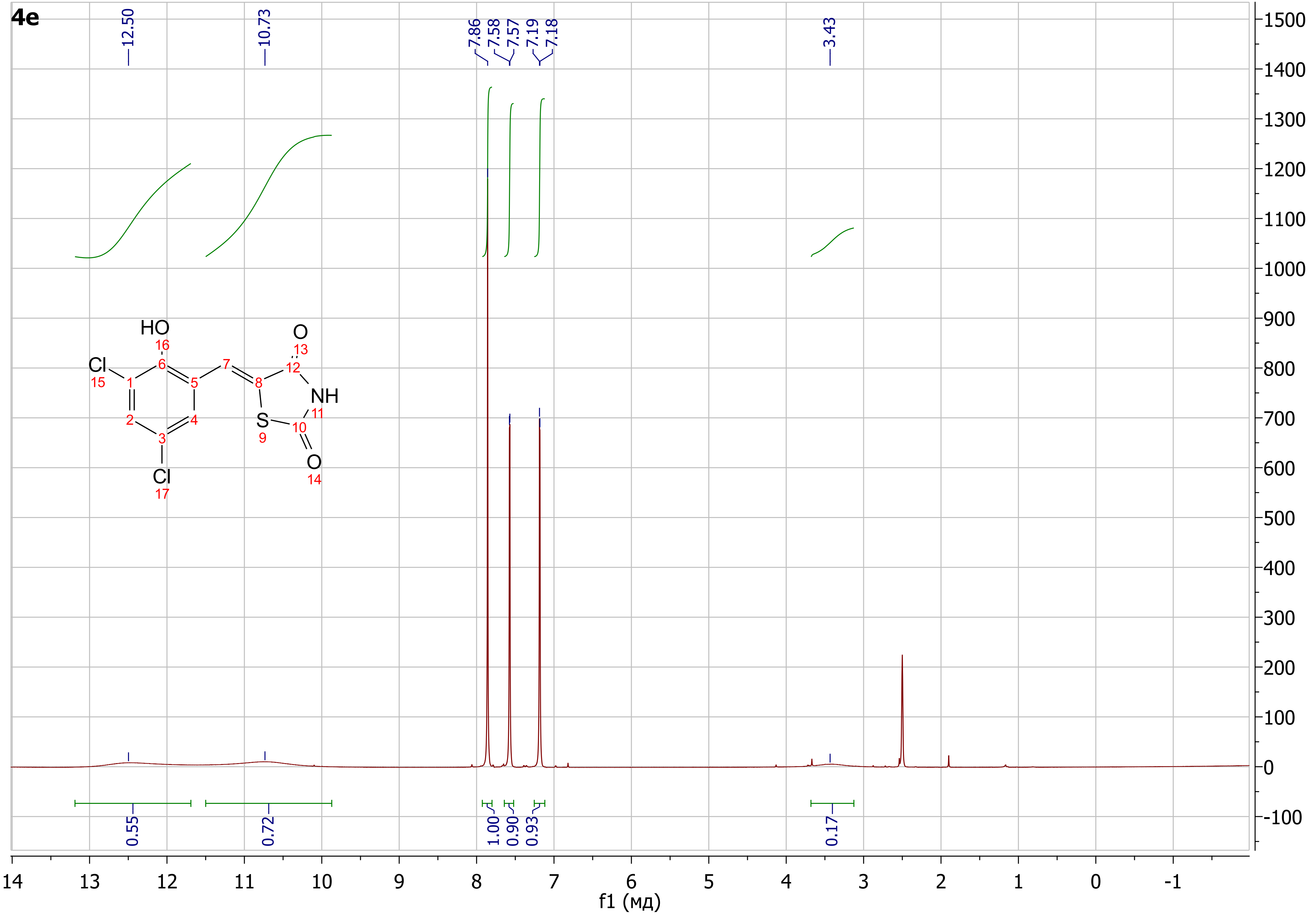

4e
12.50
10.73
7.86
7.58
7.57
7.19
7.18
3.43
HO
Cl
Cl
NH
S
O
O
0.55
0.72
1.00
0.90
0.93
0.17
f1 (мд)

4e

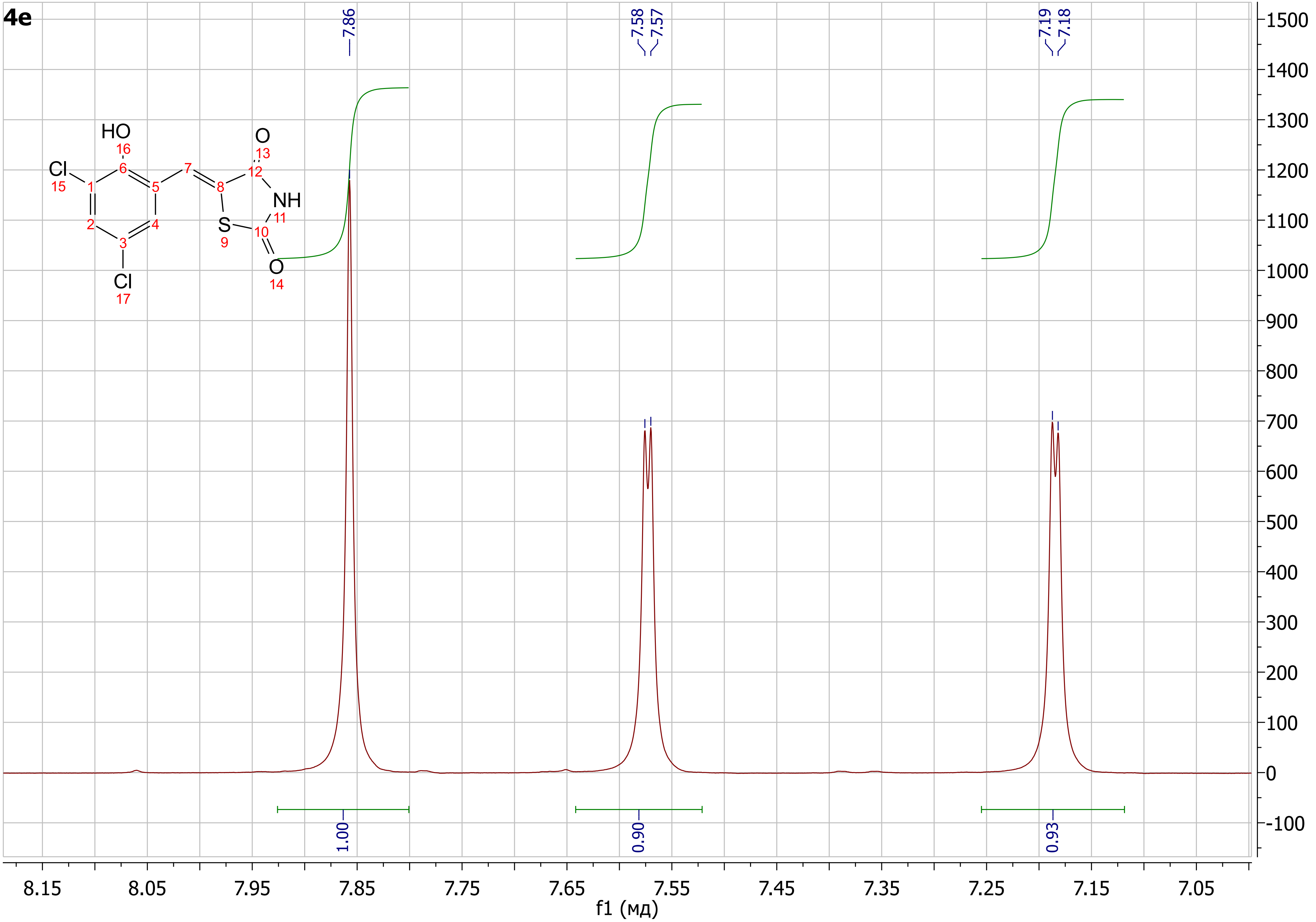

7.86
7.58
7.57
7.19
7.18
HO
16
Cl
15
6
1
5
7
8
O
13
12
NH
11
2
4
3
S
9
10
O
14
Cl
17
1.00
0.90
0.93
1500
1400
1300
1200
1100
1000
900
800
700
600
500
400
300
200
100
0
-100
8.15
8.05
7.95
7.85
7.75
7.65
7.55
7.45
7.35
7.25
7.15
7.05
f1 (мд)

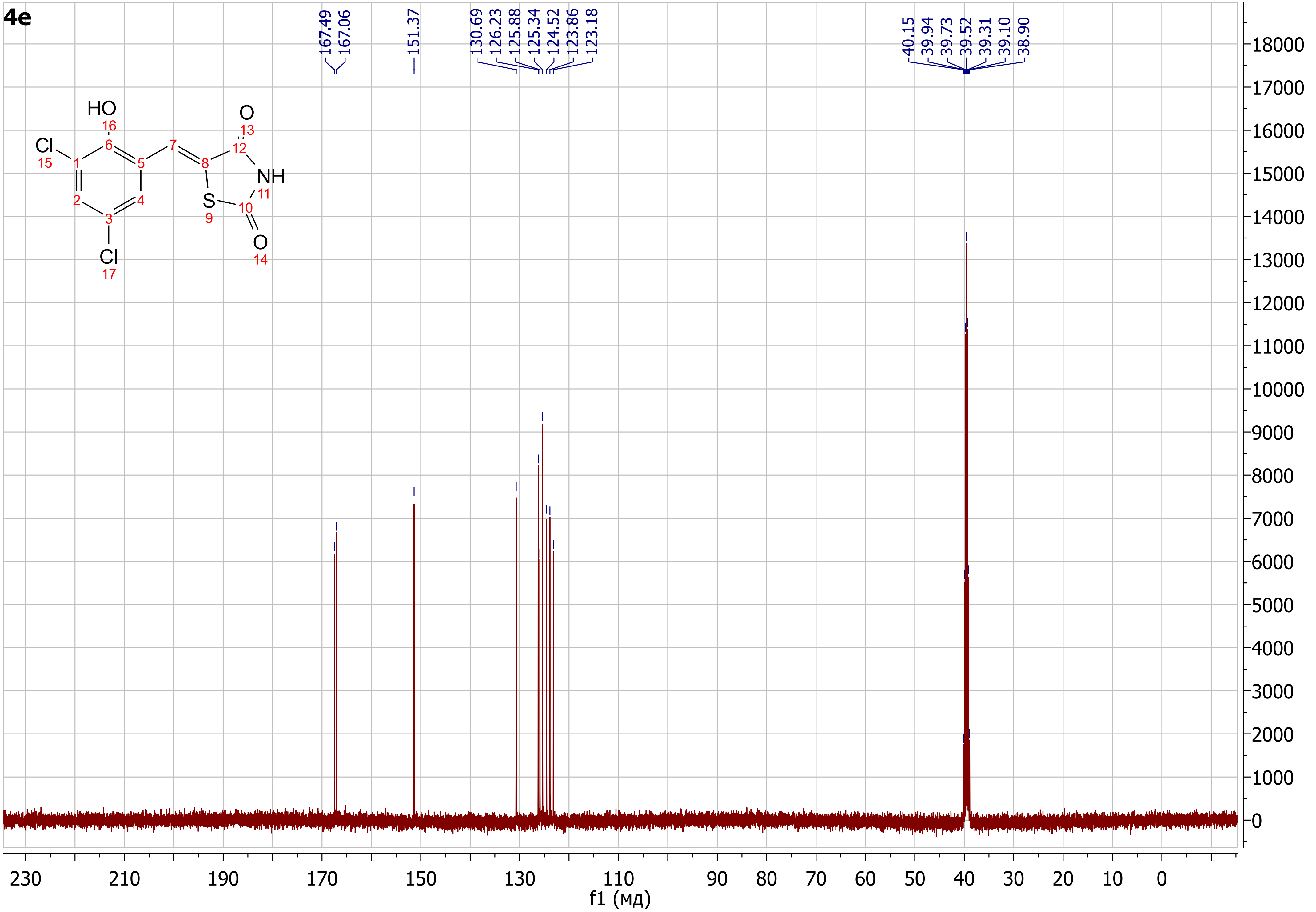
4e
HO
Cl
NH
S
O
O
Cl
167.49
167.06
151.37
130.69
126.23
125.88
125.34
124.52
123.86
123.18
40.15
39.94
39.73
39.52
39.31
39.10
38.90
f1 (мд)

4e

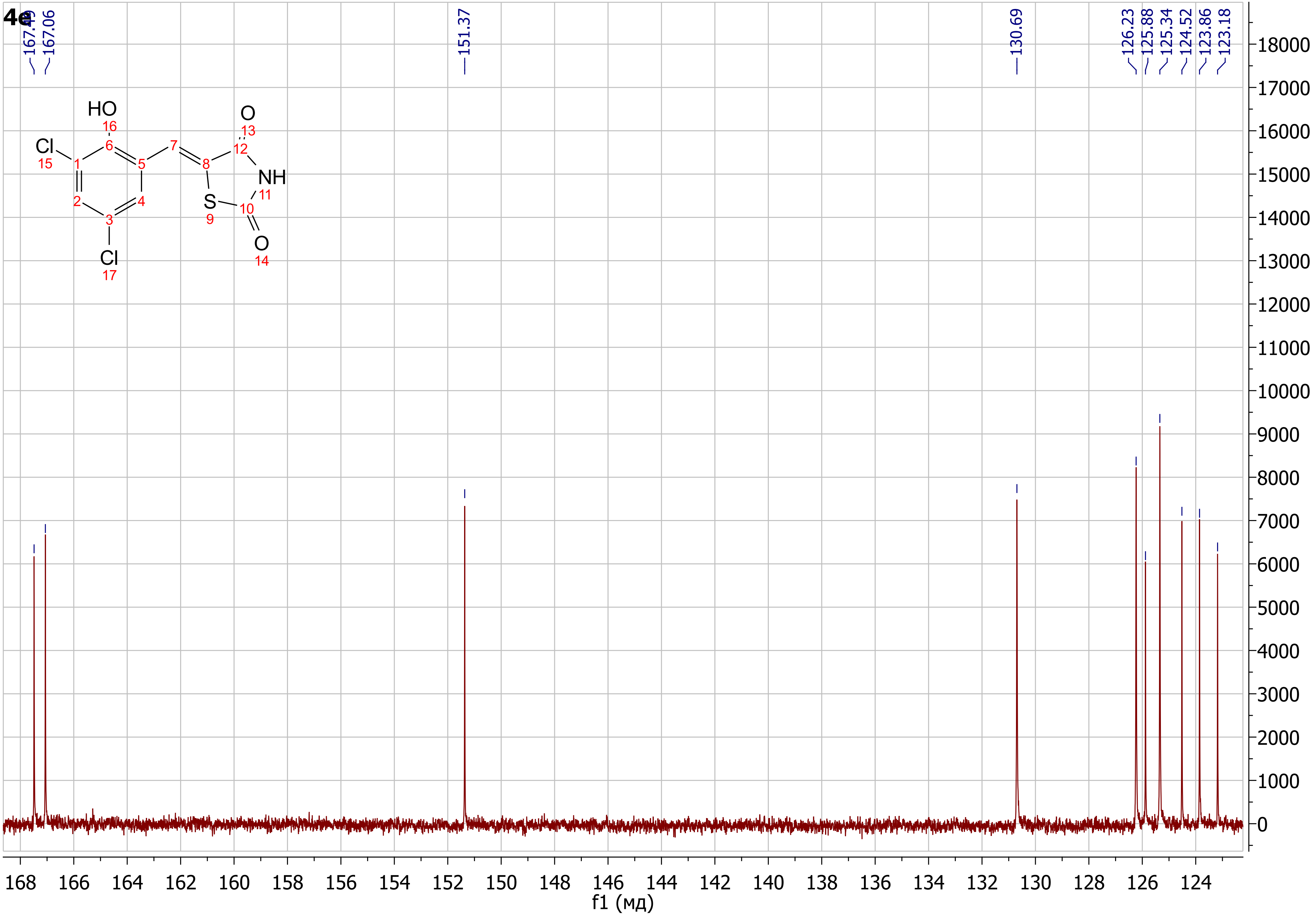
167.49
167.06
151.37
130.69
126.23
125.88
125.34
124.52
123.86
123.18
HO
16
Cl
15
1
2
3
4
5
6
7
8
9
10
11
12
O
13
NH
S
O
14
Cl
17
18000
17000
16000
15000
14000
13000
12000
11000
10000
9000
8000
7000
6000
5000
4000
3000
2000
1000
0
168
166
164
162
160
158
156
154
152
150
148
146
144
142
140
138
136
134
132
130
128
126
124
f1 (мд)

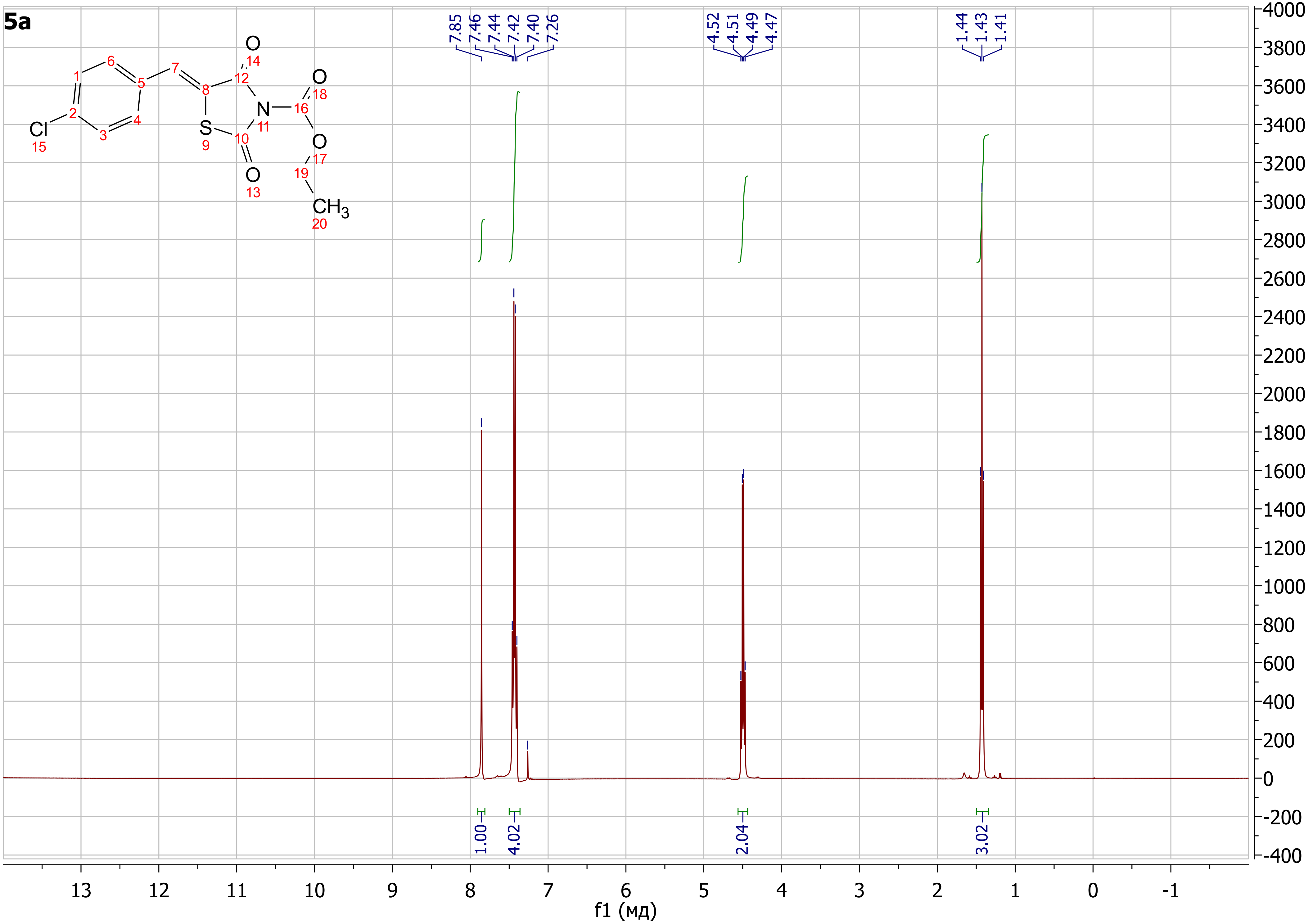
5a
7.85
7.46
7.44
7.42
7.40
7.26
4.52
4.51
4.49
4.47
1.44
1.43
1.41
1.00
4.02
2.04
3.02
Cl
O
N
S
CH3
f1 (мд)

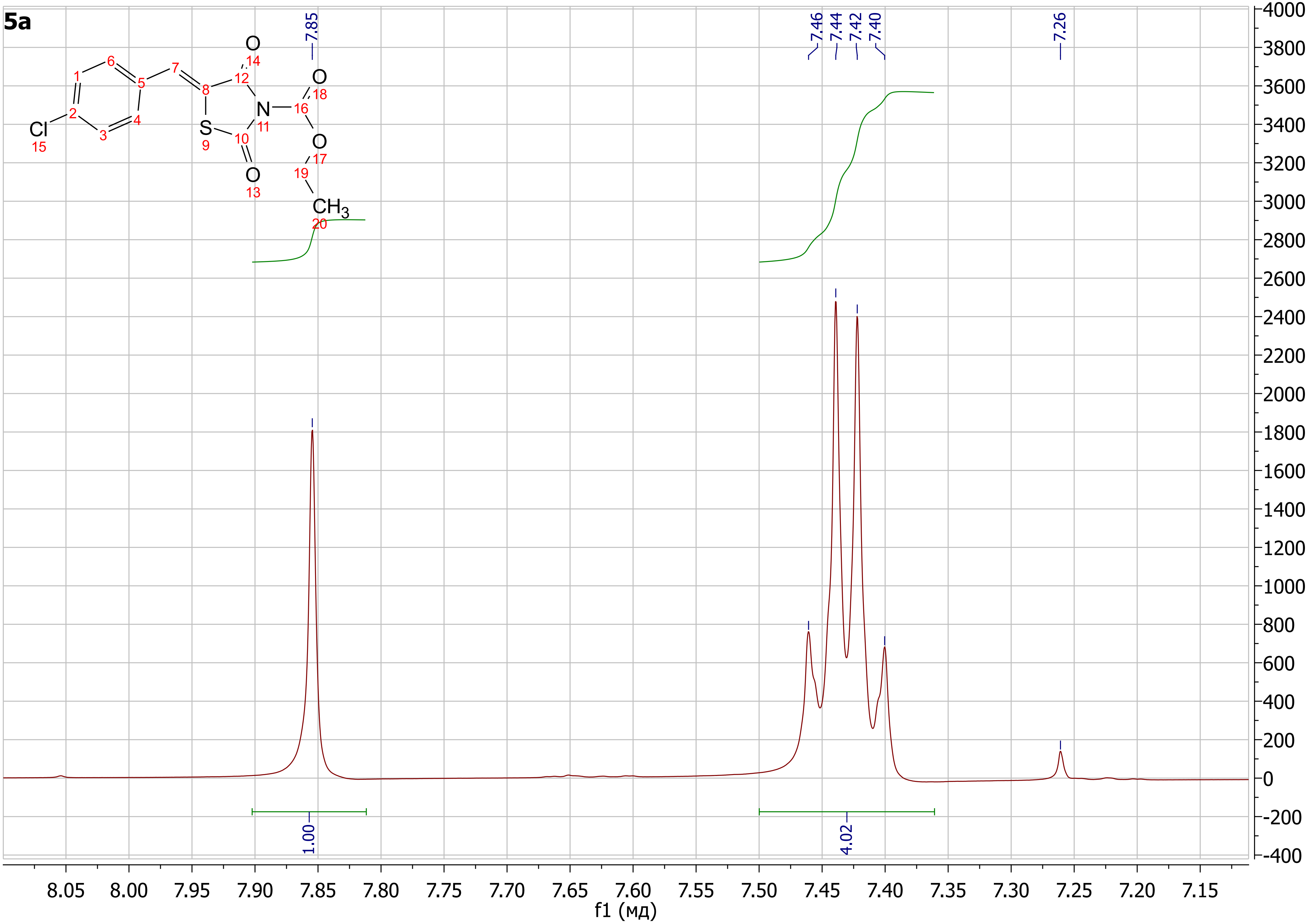

5a
7.85
7.46
7.44
7.42
7.40
7.26
1.00
4.02
f1 (мд)

5a

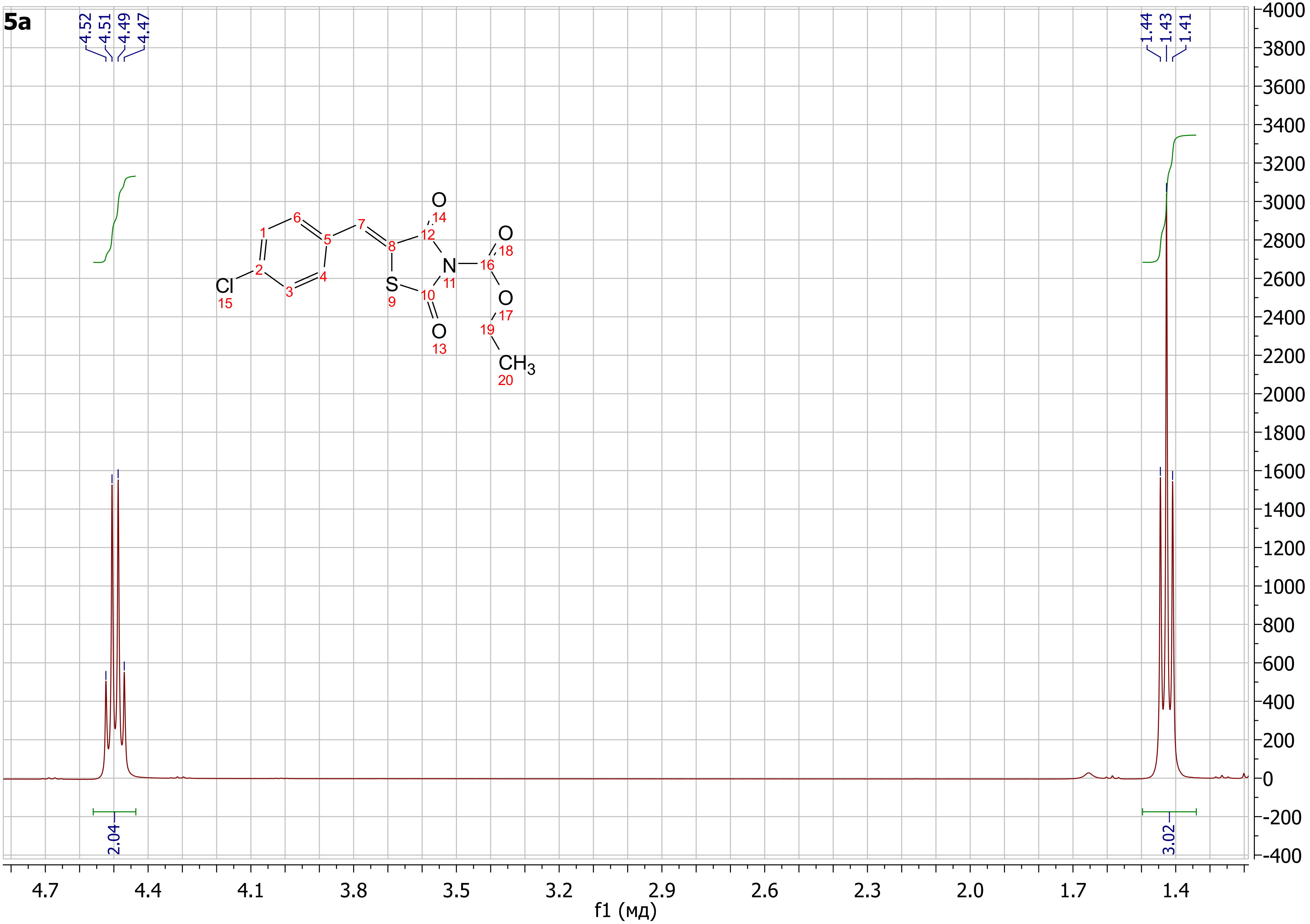

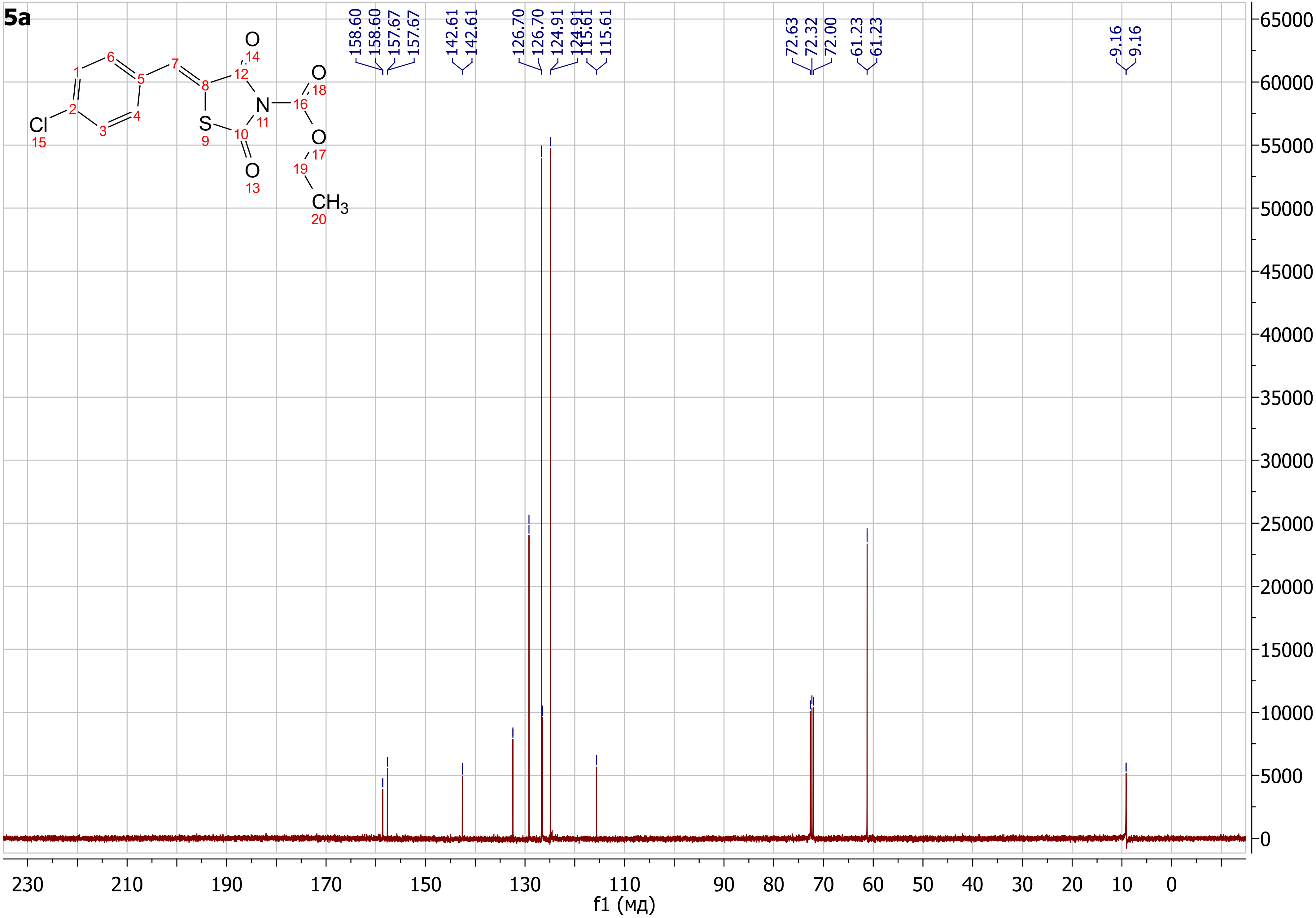

5a
Cl
CH3
158.60
158.60
157.67
157.67
142.61
142.61
126.70
126.70
124.91
124.91
115.61
115.61
72.63
72.32
72.00
61.23
61.23
9.16
9.16
f1 (мд)

5a

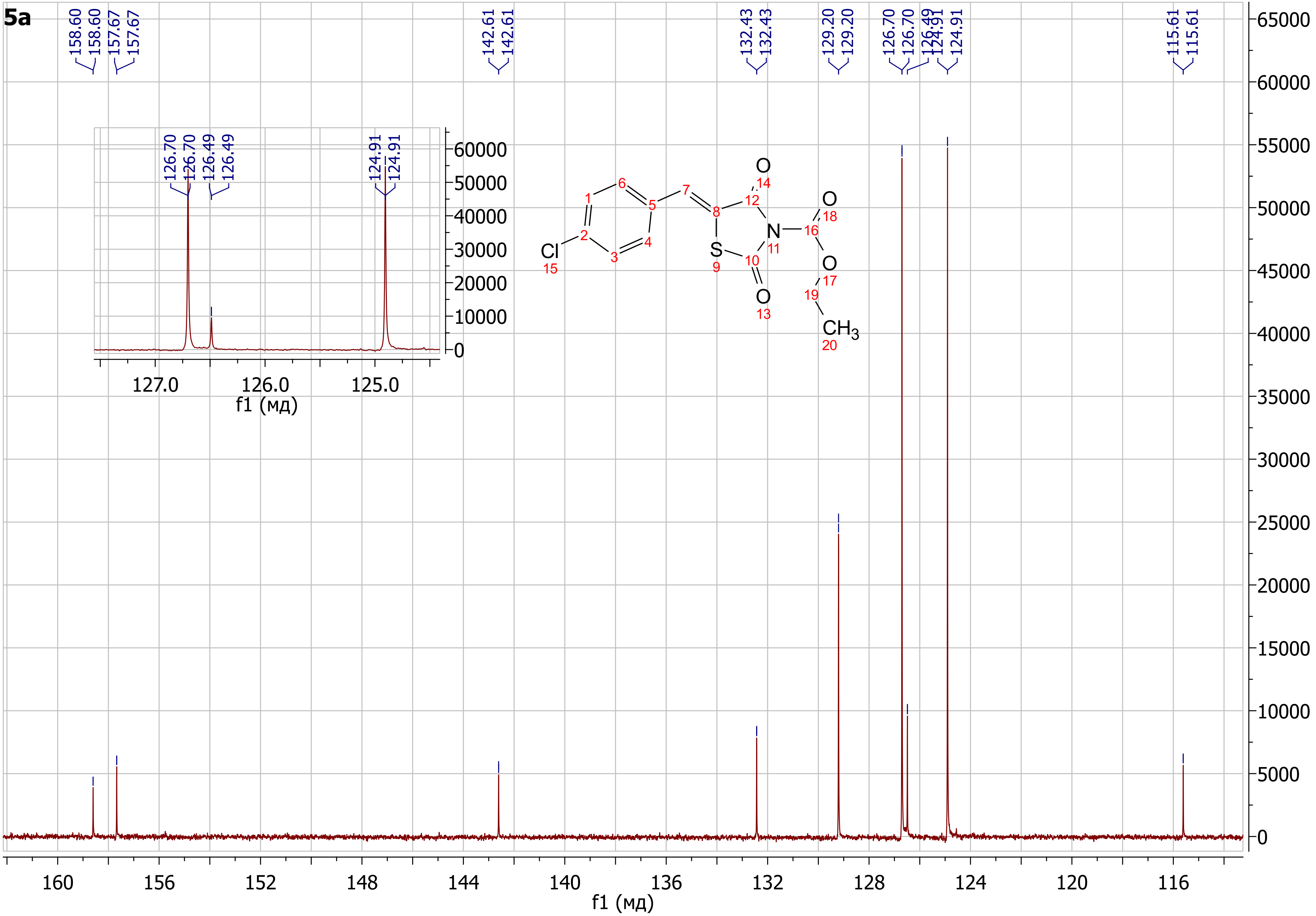

158.60
158.60
157.67
157.67
142.61
142.61
132.43
132.43
129.20
129.20
126.70
126.70
126.49
124.91
124.91
115.61
115.61
Cl
CH3
f1 (мд)

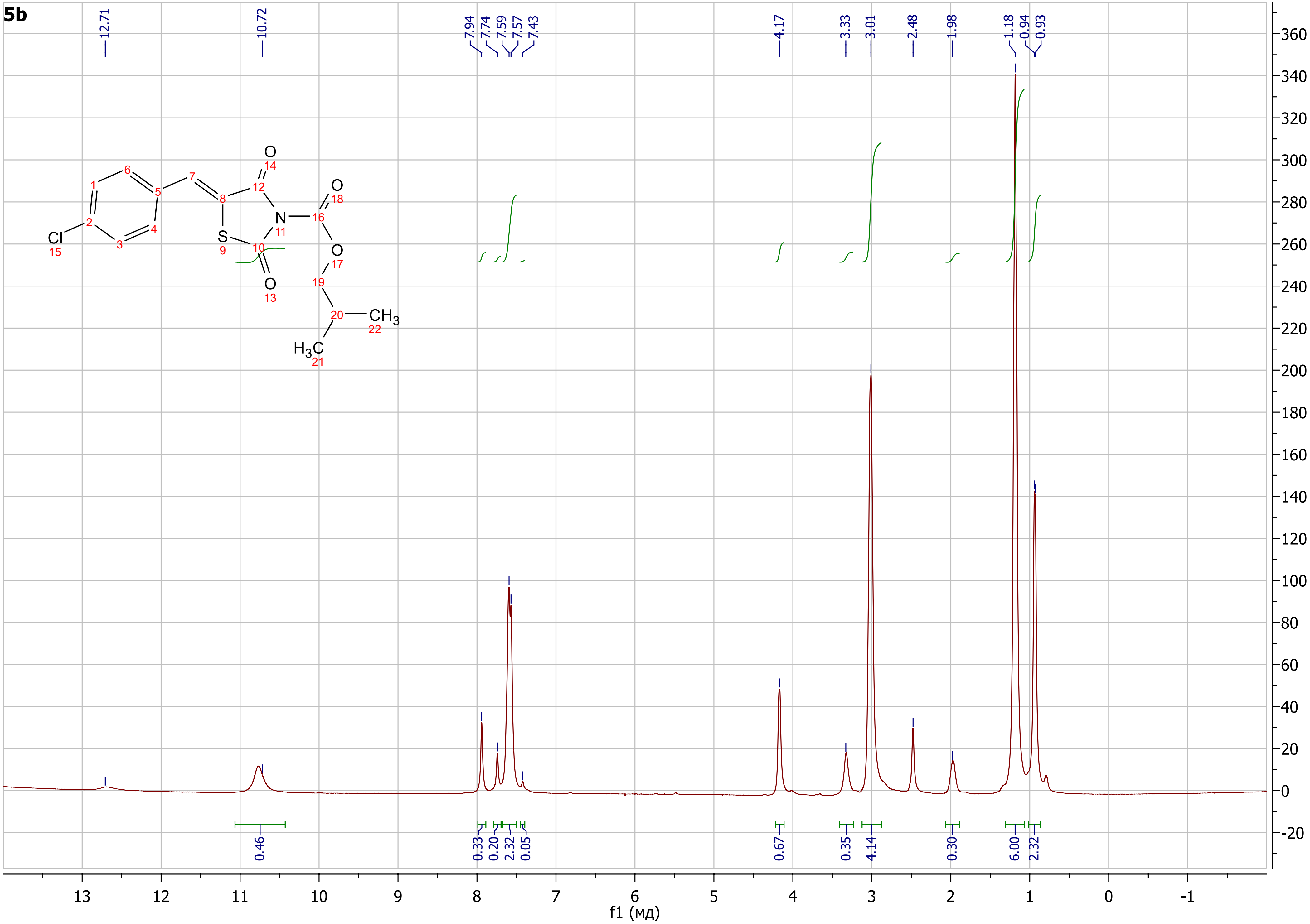

5b
12.71
10.72
7.94
7.74
7.59
7.57
7.43
4.17
3.33
3.01
2.48
1.98
1.18
0.94
0.93
0.46
0.33
0.20
2.32
0.05
0.67
0.35
4.14
0.30
6.00
2.32
f1 (мд)

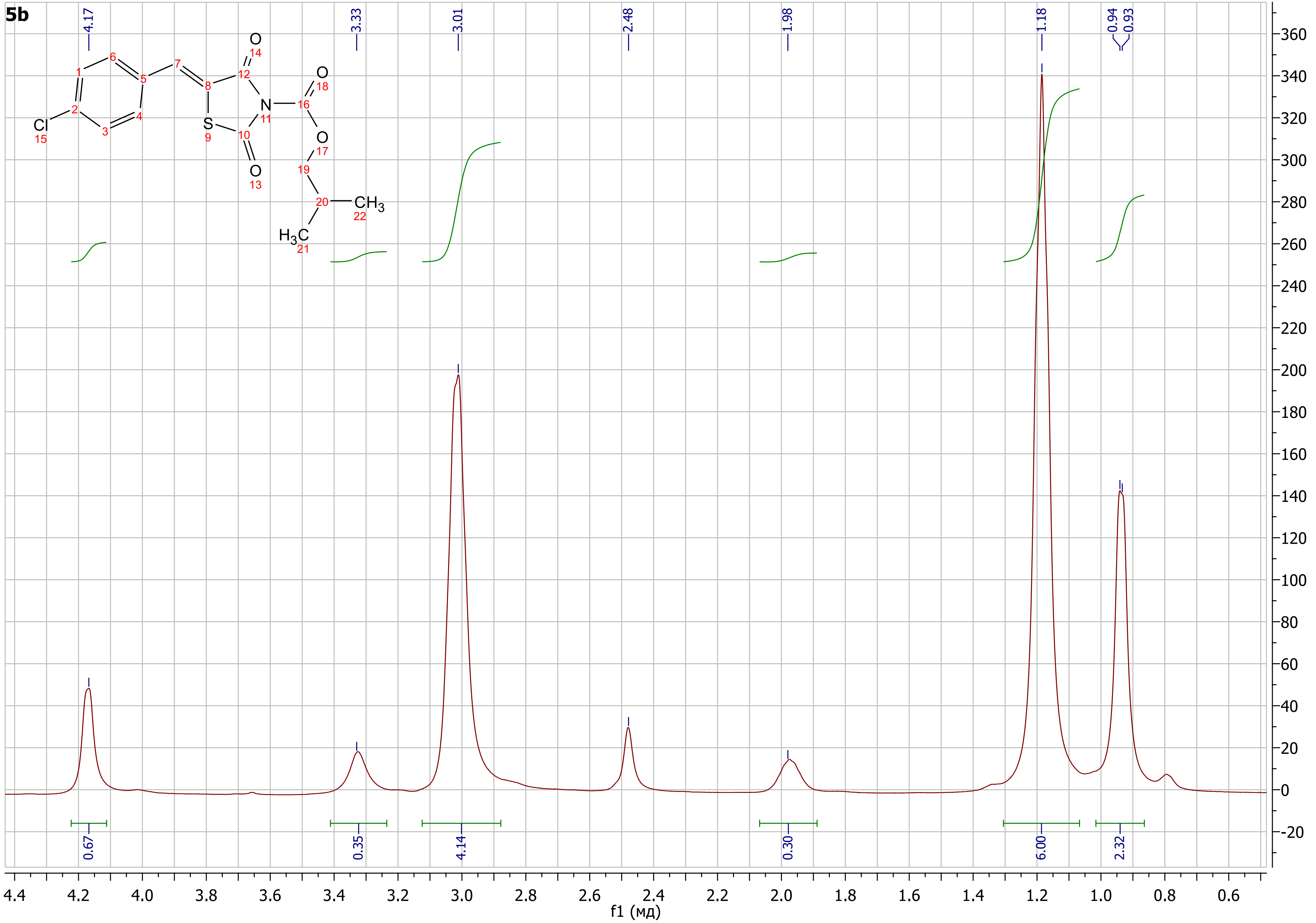

5b
4.17
3.33
3.01
2.48
1.98
1.18
0.94
0.93
Cl
H_3C
CH_3
0.67
0.35
4.14
0.30
6.00
2.32
f1 (мд)

5b

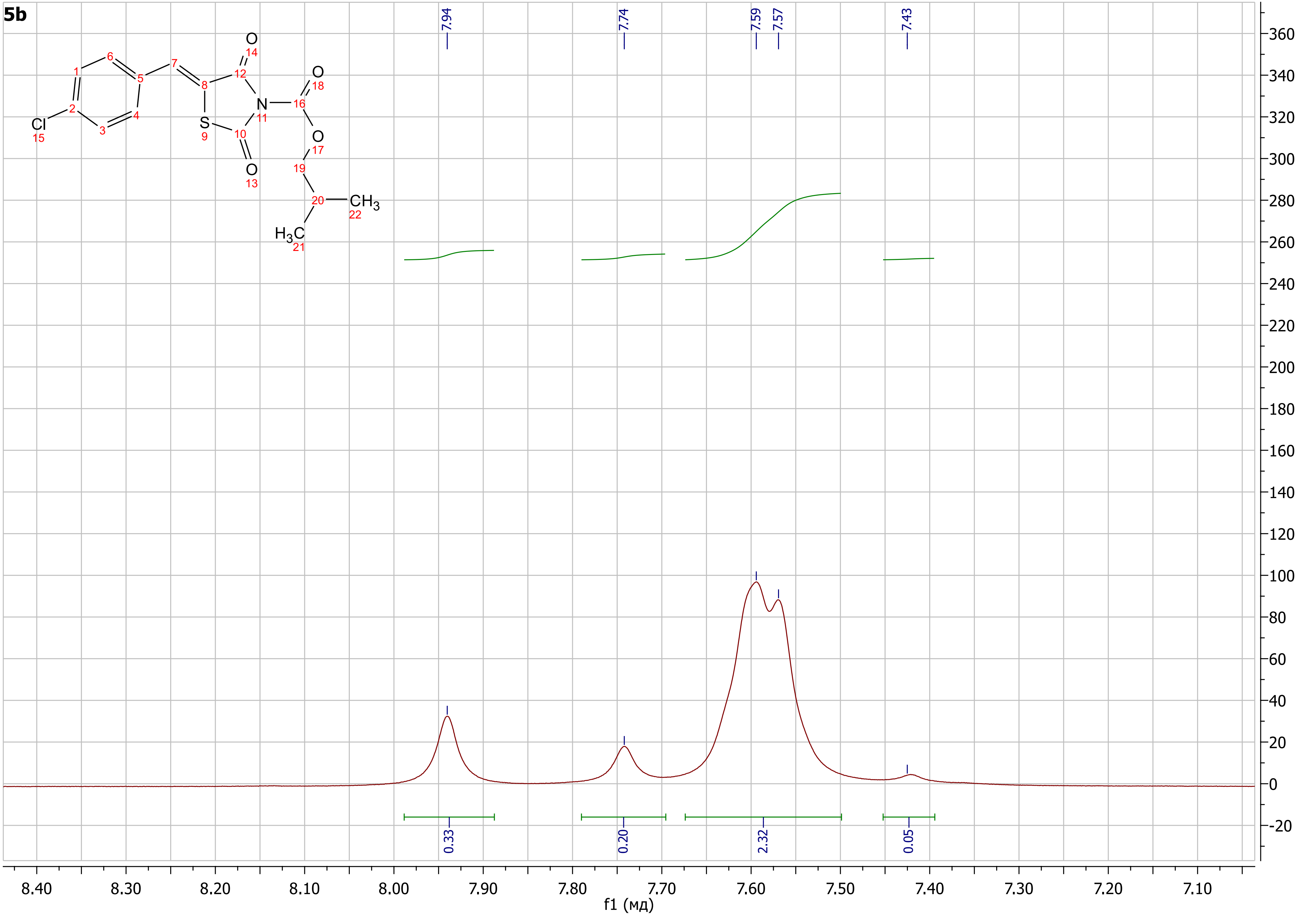

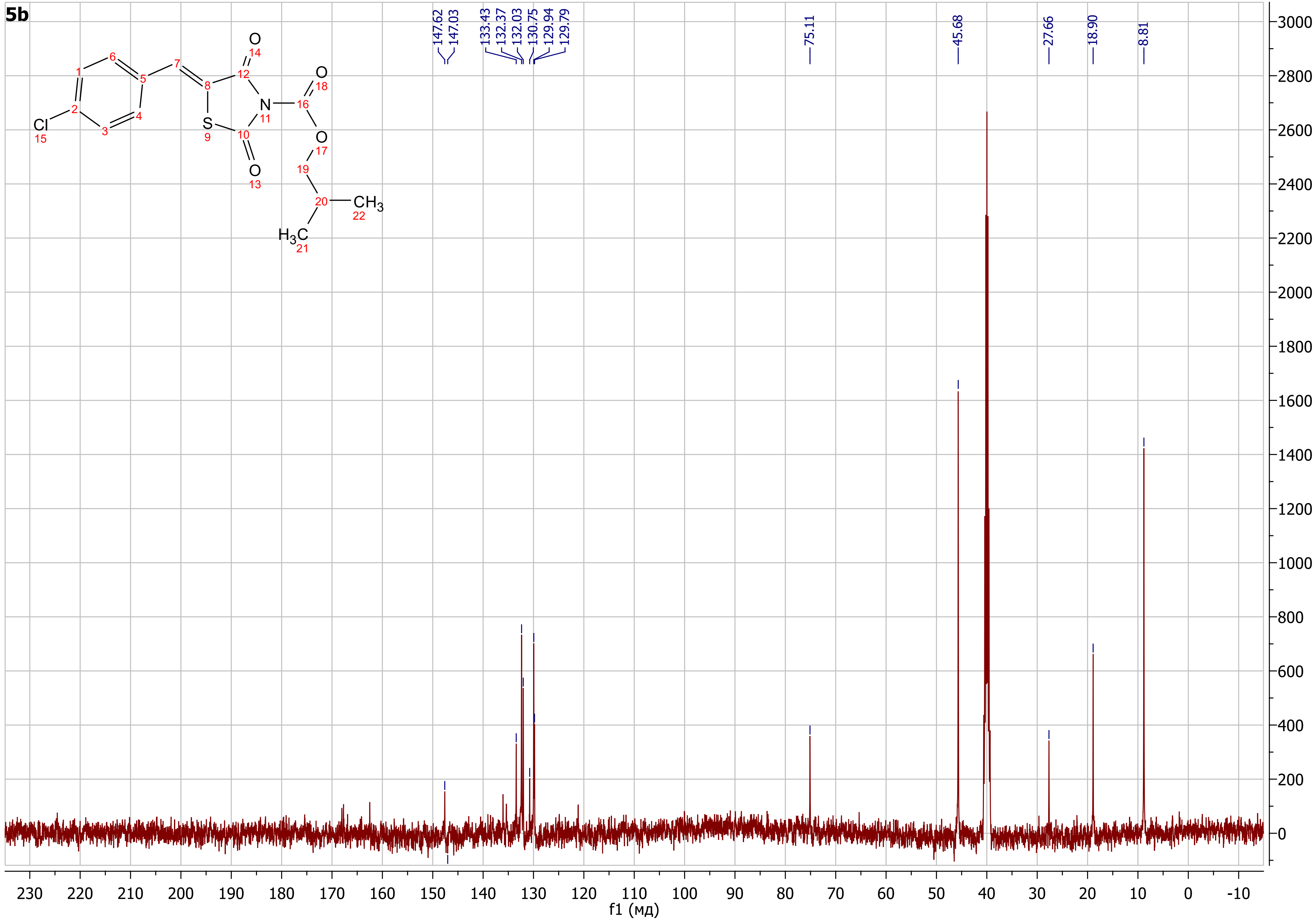

5b
147.62
147.03
133.43
132.37
132.03
130.75
129.94
129.79
75.11
45.68
27.66
18.90
8.81
Cl
O
N
S
CH3
H3C
f1 (мд)

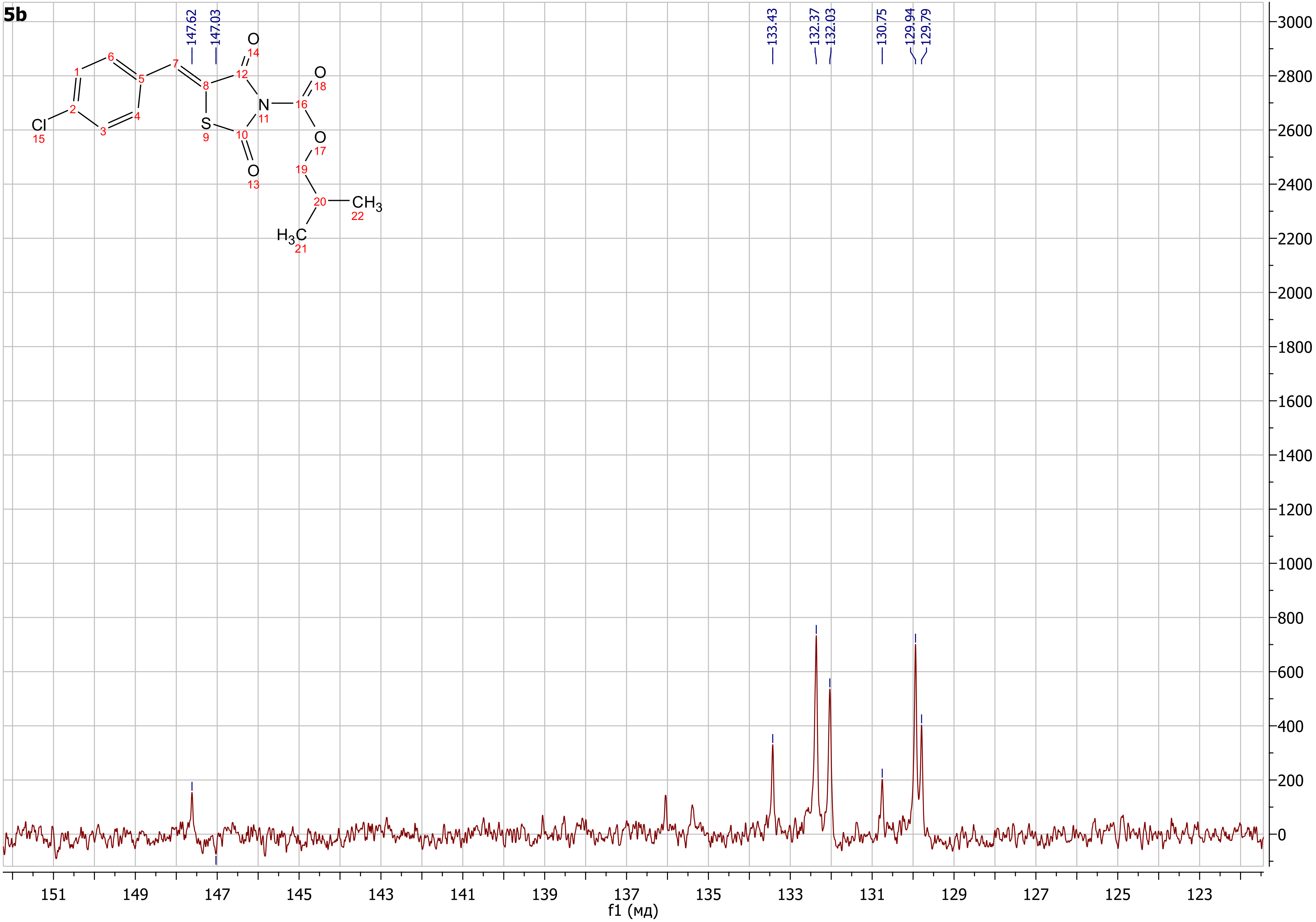

5b
147.62
147.03
133.43
132.37
132.03
130.75
129.94
129.79
O
N
S
Cl
CH3
H3C
1 2 3 4 5 6 7 8 9 10 11 12 13 14 15 16 17 18 19 20 21 22
3000
2800
2600
2400
2200
2000
1800
1600
1400
1200
1000
800
600
400
200
0
151
149
147
145
143
141
139
137
135
133
131
129
127
125
123
f1 (мд)

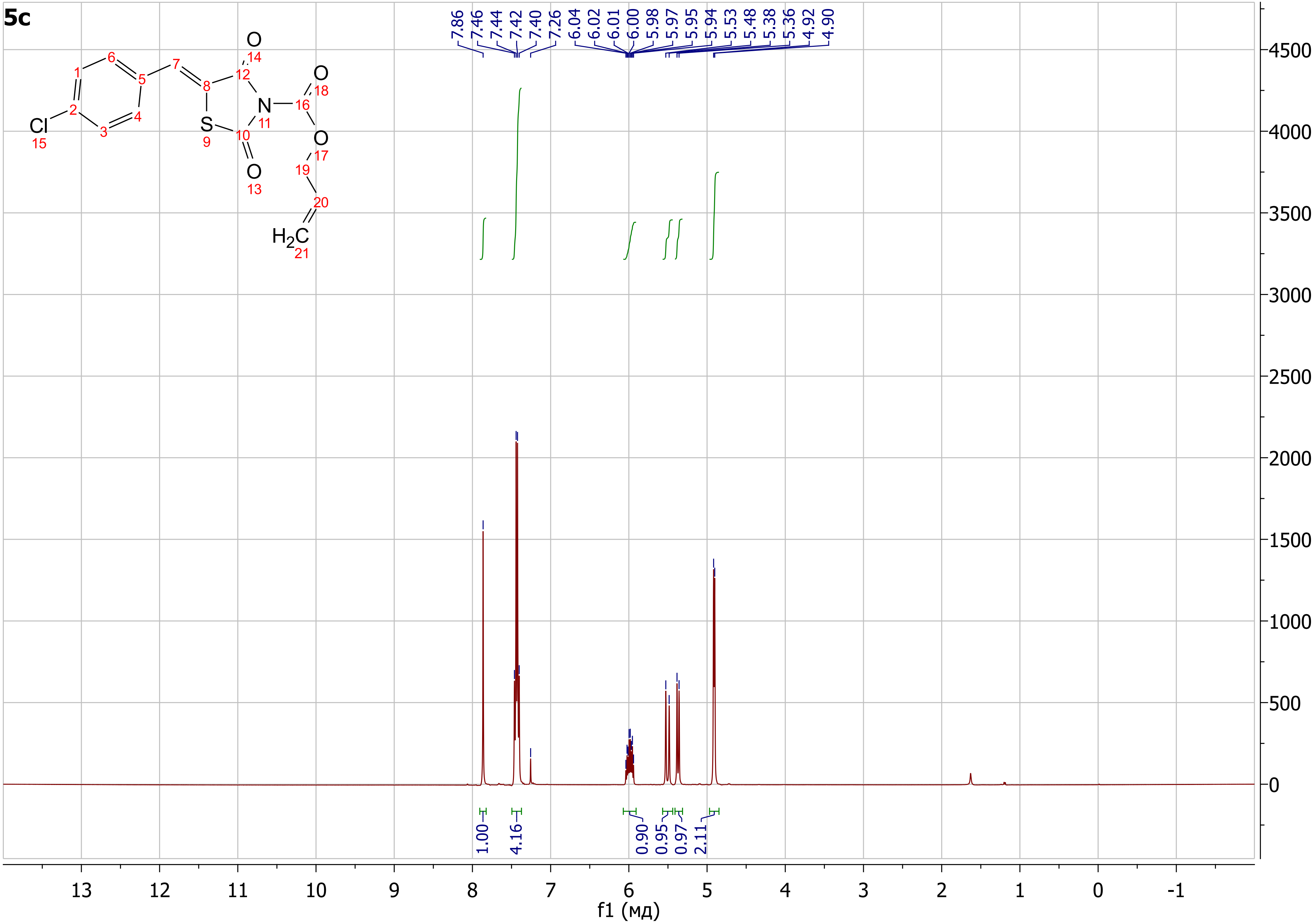

5c
Cl
H2C
f1 (мд)
7.86
7.46
7.44
7.42
7.40
7.26
6.04
6.02
6.01
6.00
5.98
5.97
5.95
5.94
5.53
5.48
5.38
5.36
4.92
4.90
1.00
4.16
0.90
0.95
0.97
2.11

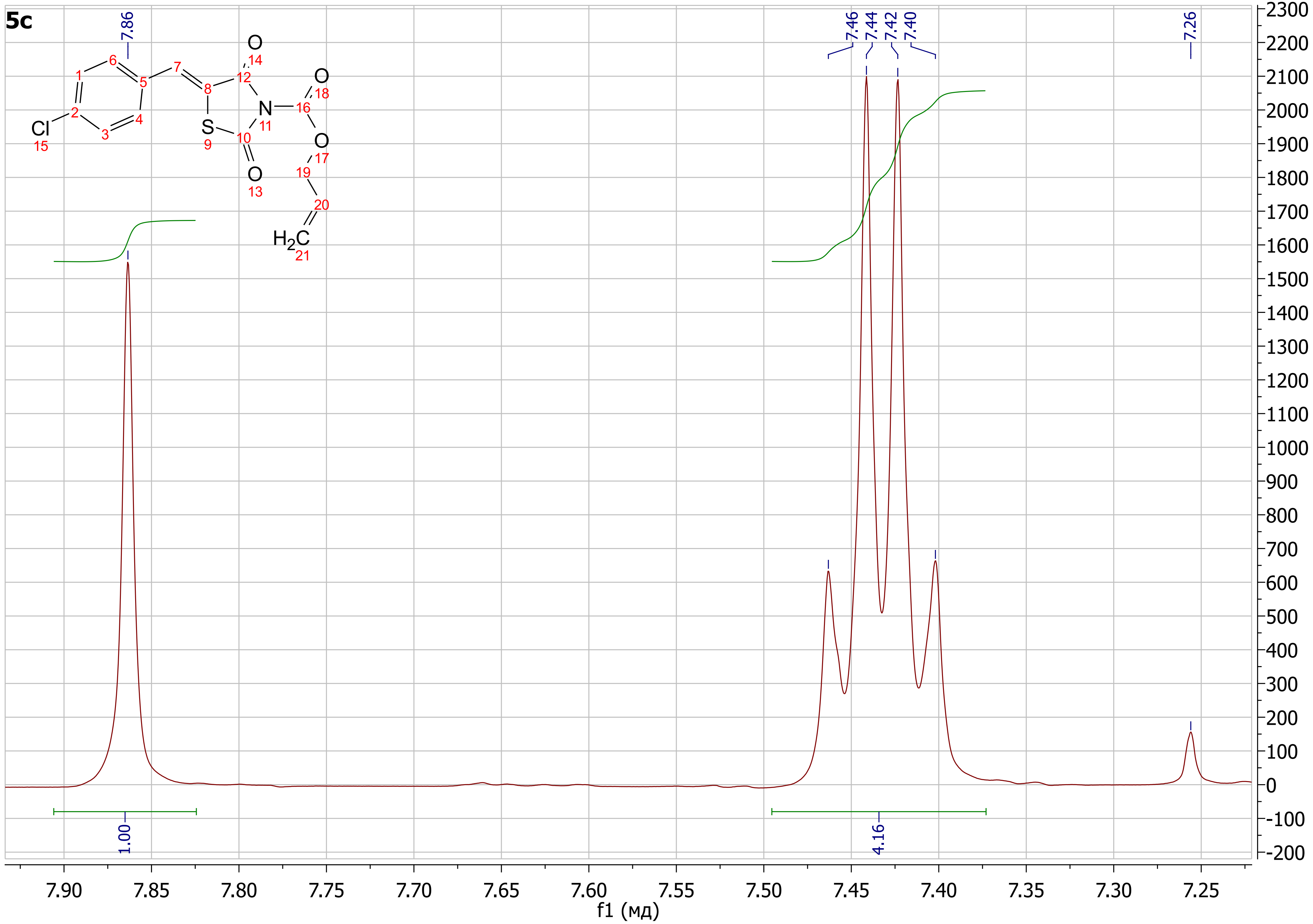

5c
7.86
7.46
7.44
7.42
7.40
7.26
1.00
4.16
f1 (мд)

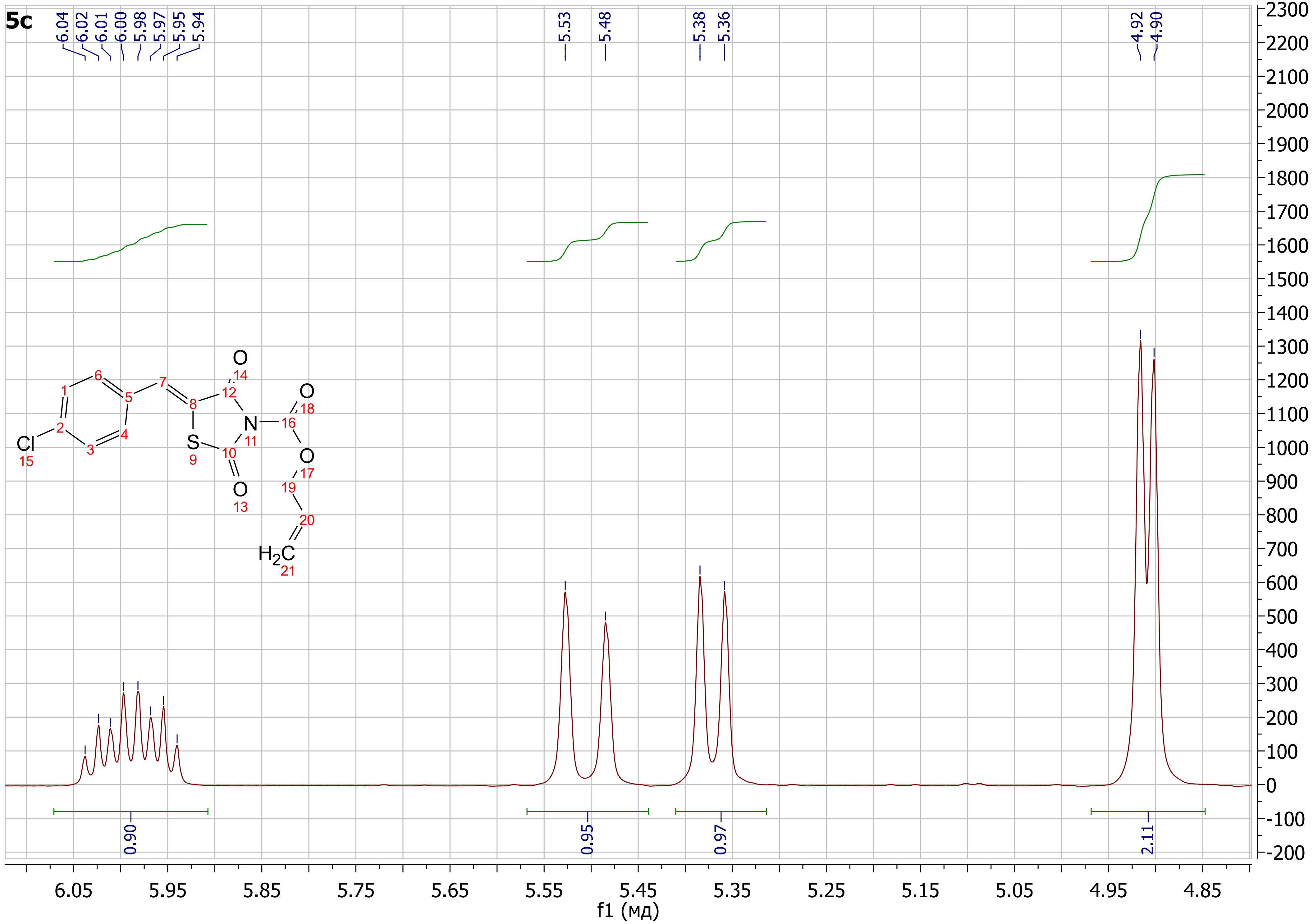
5c
6.04
6.02
6.01
6.00
5.98
5.97
5.95
5.94
5.53
5.48
5.38
5.36
4.92
4.90
0.90
0.95
0.97
2.11
Cl
O
N
S
H2C
f1 (мд)

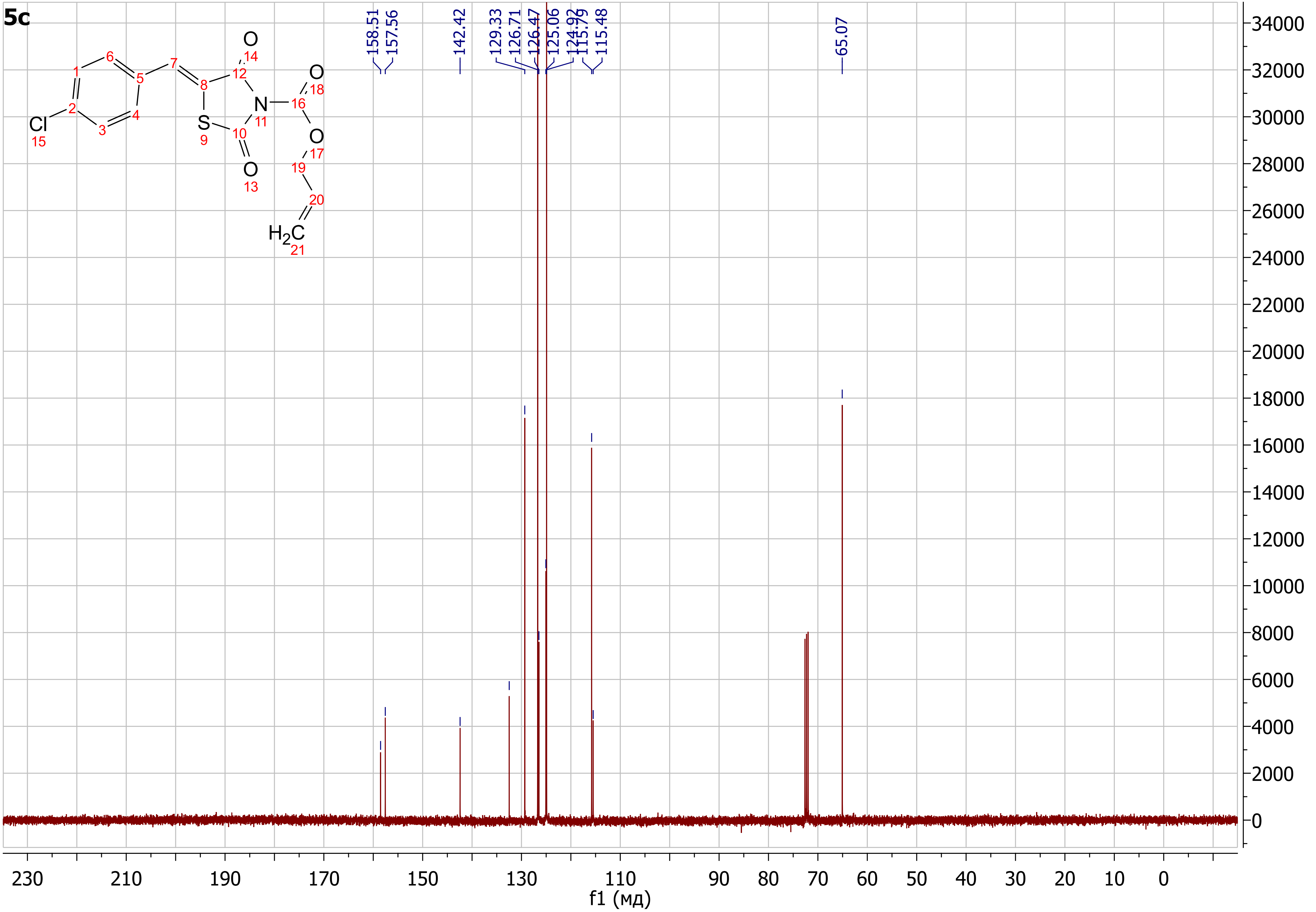

5c
Cl
S
N
O
H2C
158.51
157.56
142.42
129.33
126.71
126.47
125.06
124.92
115.79
115.48
65.07
f1 (мд)

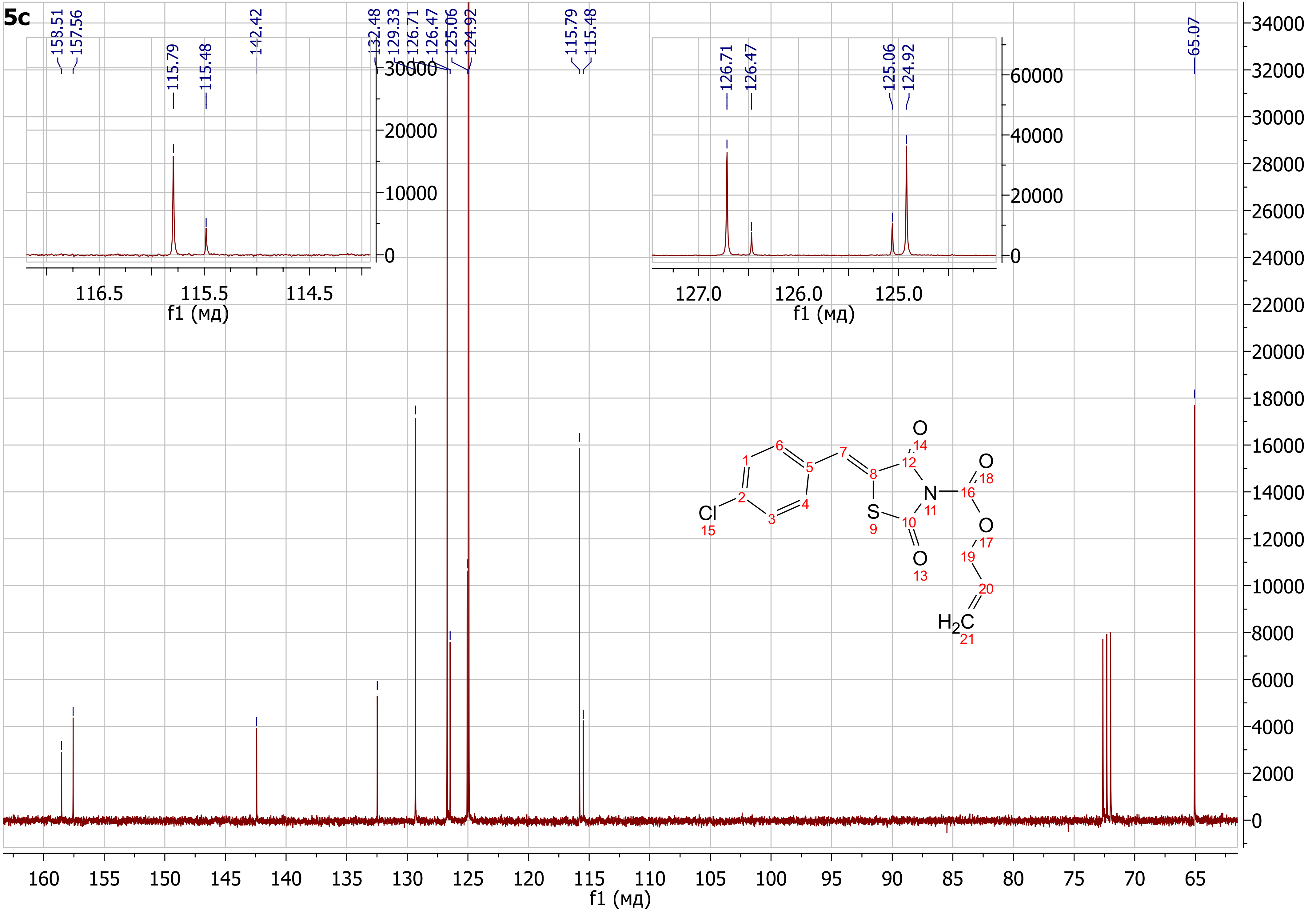
5c
158.51
157.56
142.42
132.48
129.33
126.71
126.47
125.06
124.92
115.79
115.48
65.07
f1 (мд)
Cl
S
N
O
H2C

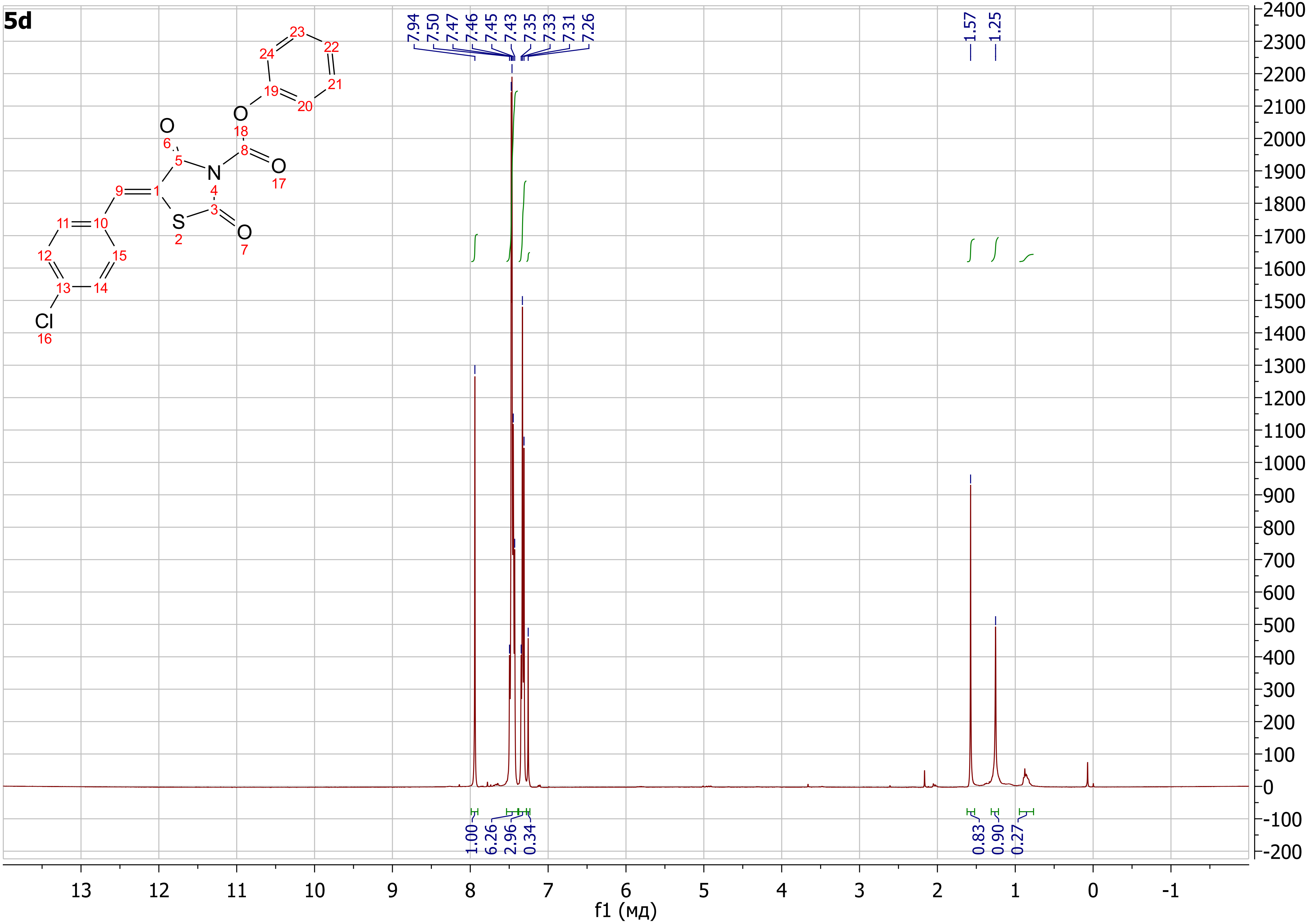
5d
7.94
7.50
7.47
7.46
7.45
7.43
7.35
7.33
7.31
7.26
1.57
1.25
1.00
6.26
2.96
0.34
0.83
0.90
0.27
f1 (мд)

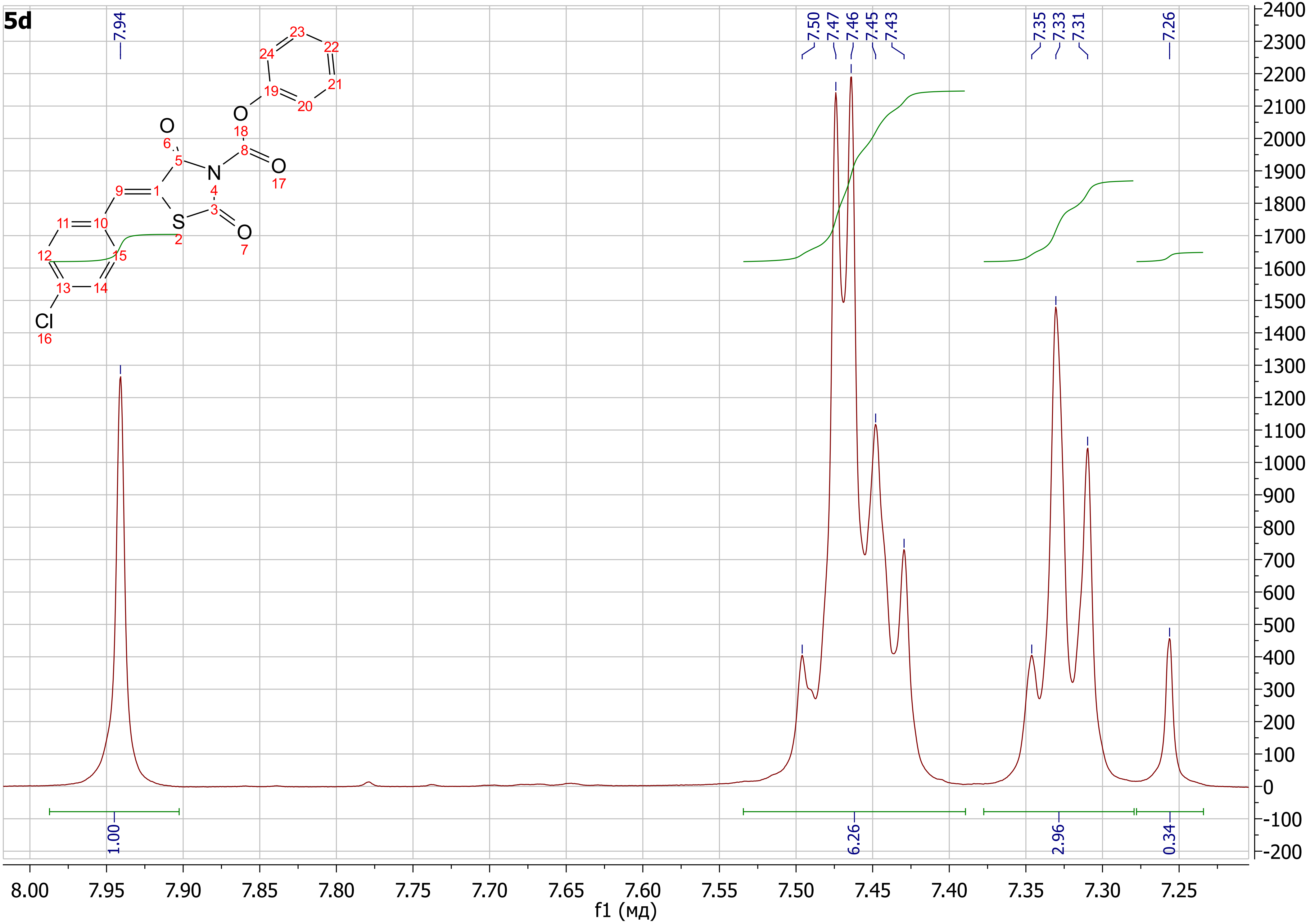

5d
7.94
7.50
7.47
7.46
7.45
7.43
7.35
7.33
7.31
7.26
1.00
6.26
2.96
0.34
f1 (мд)

5d

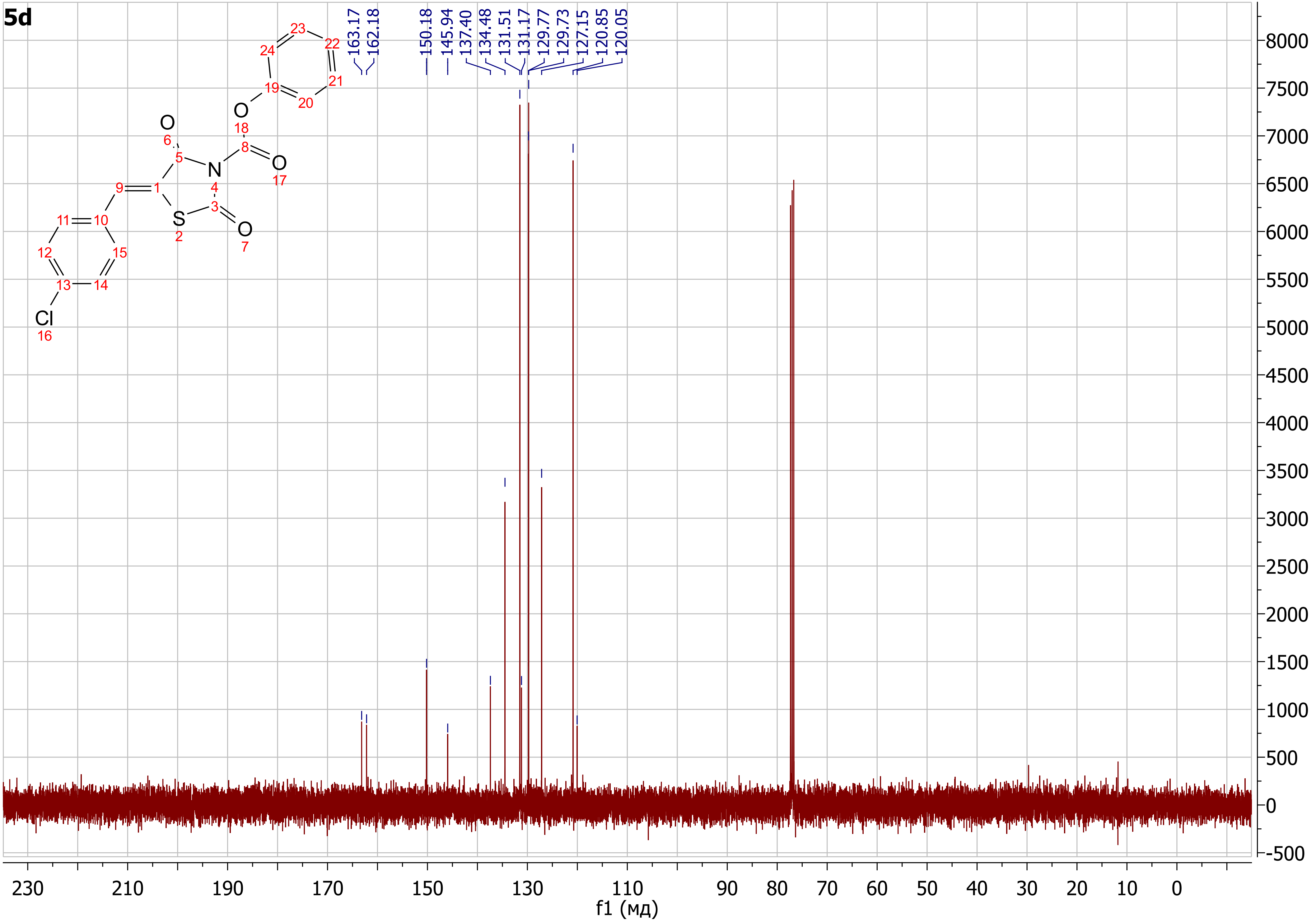

163.17
162.18
150.18
145.94
137.40
134.48
131.51
131.17
129.77
129.73
127.15
120.85
120.05
f1 (мд)

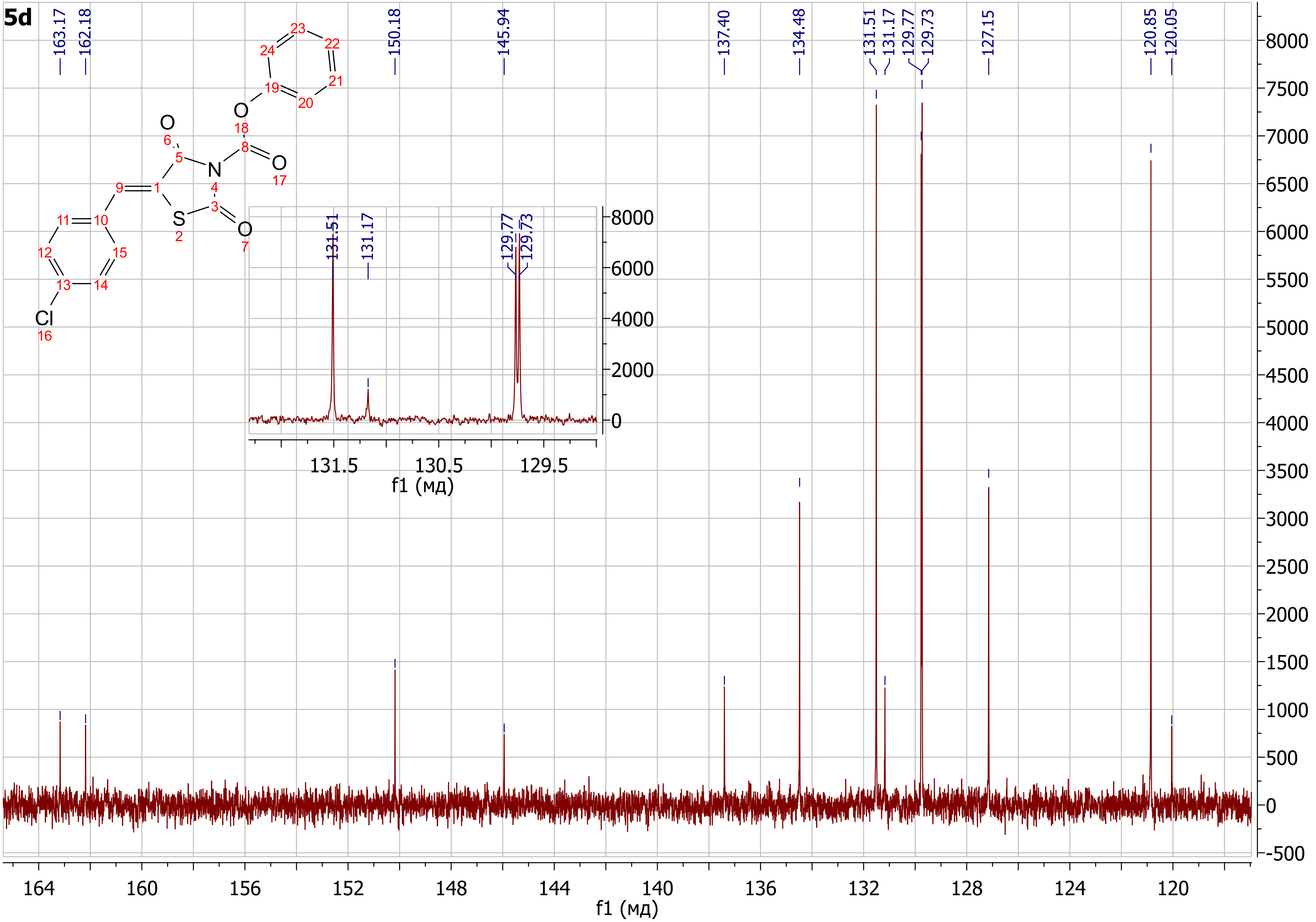

5d
163.17
162.18
150.18
145.94
137.40
134.48
131.51
131.17
129.77
129.73
127.15
120.85
120.05
O
N
S
Cl
131.5
130.5
129.5
f1 (мд)
164
160
156
152
148
144
140
136
132
128
124
120
f1 (мд)

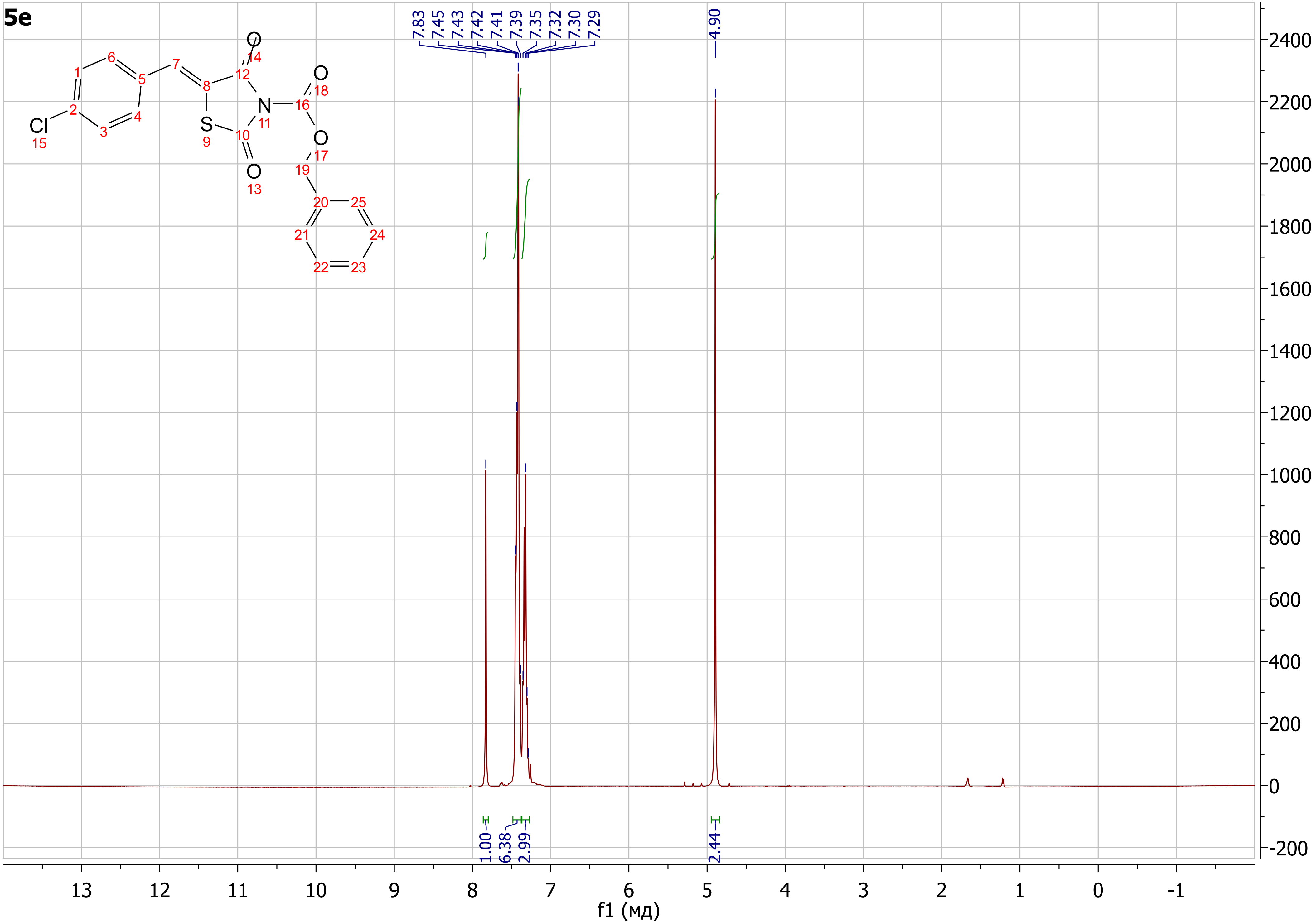

5e
Cl
O
S
N
7.83
7.45
7.43
7.42
7.41
7.39
7.35
7.32
7.30
7.29
4.90
1.00
6.38
2.99
2.44
f1 (мд)

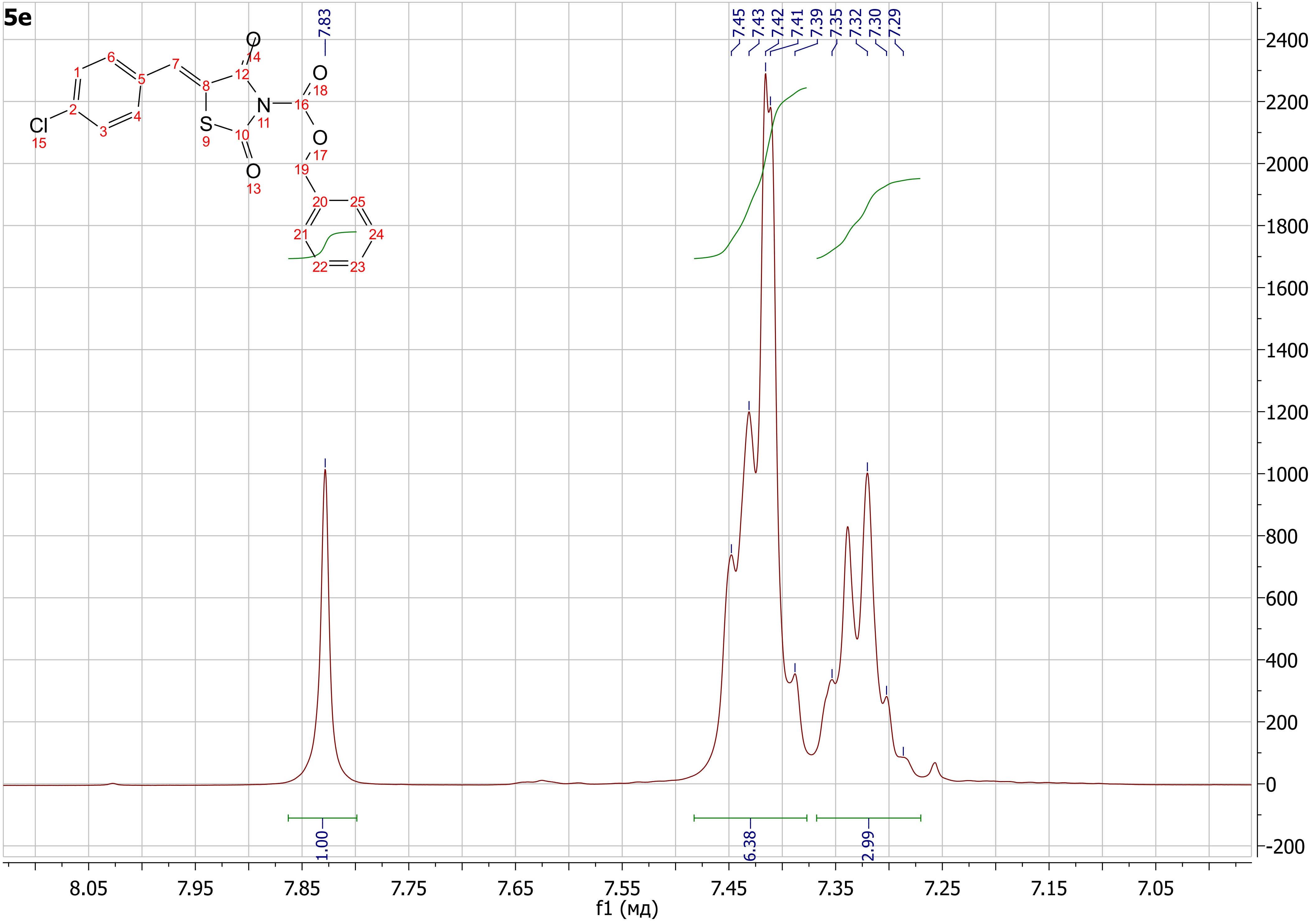

5e
Cl
S
N
O
7.83
7.45
7.43
7.42
7.41
7.39
7.35
7.32
7.30
7.29
1.00
6.38
2.99
8.05
7.95
7.85
7.75
7.65
7.55
7.45
7.35
7.25
7.15
7.05
f1 (мд)
2400
2200
2000
1800
1600
1400
1200
1000
800
600
400
200
0
-200

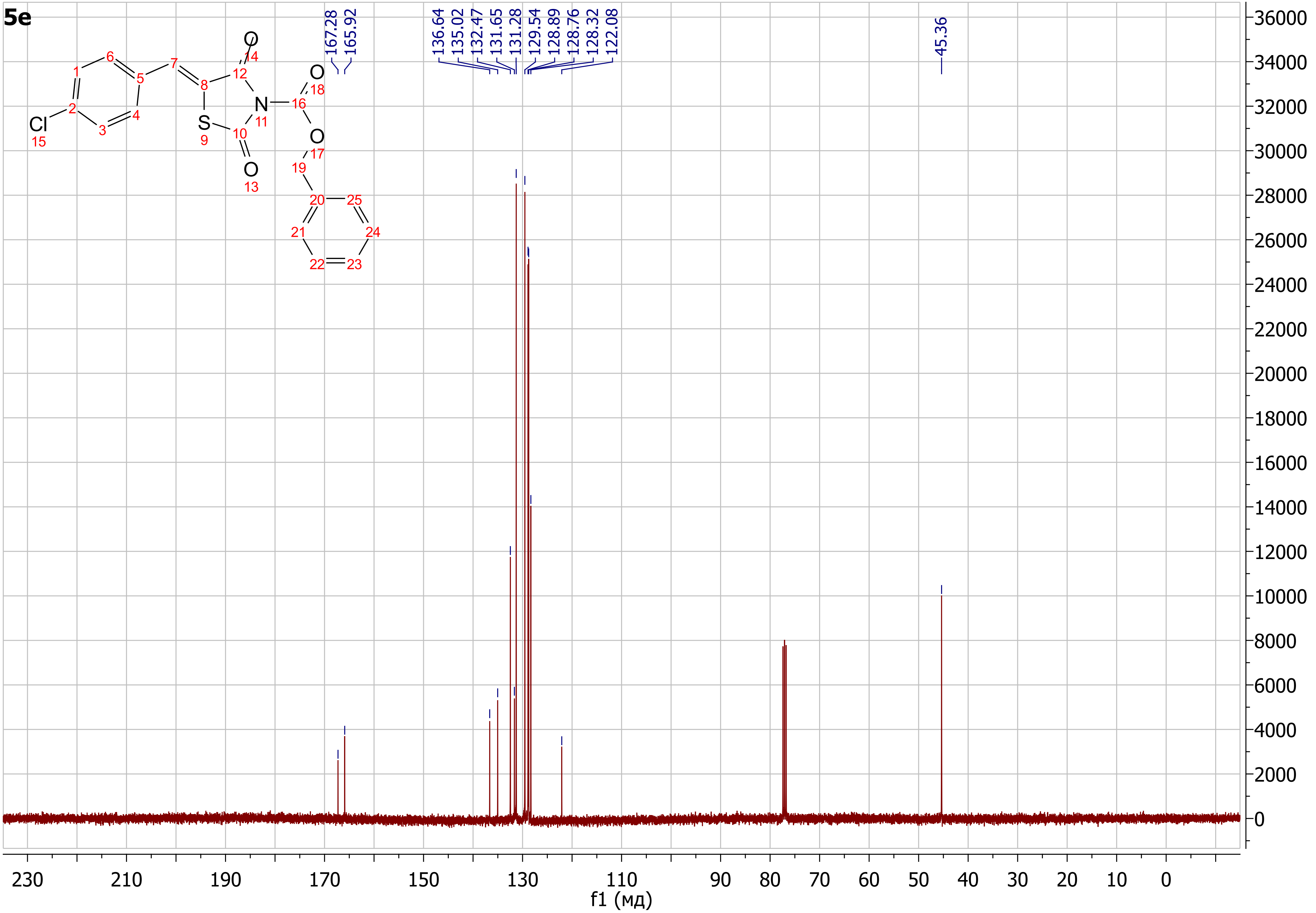

5e
167.28
165.92
136.64
135.02
132.47
131.65
131.28
129.54
128.89
128.76
128.32
122.08
45.36
Cl
S
N
O
f1 (мд)

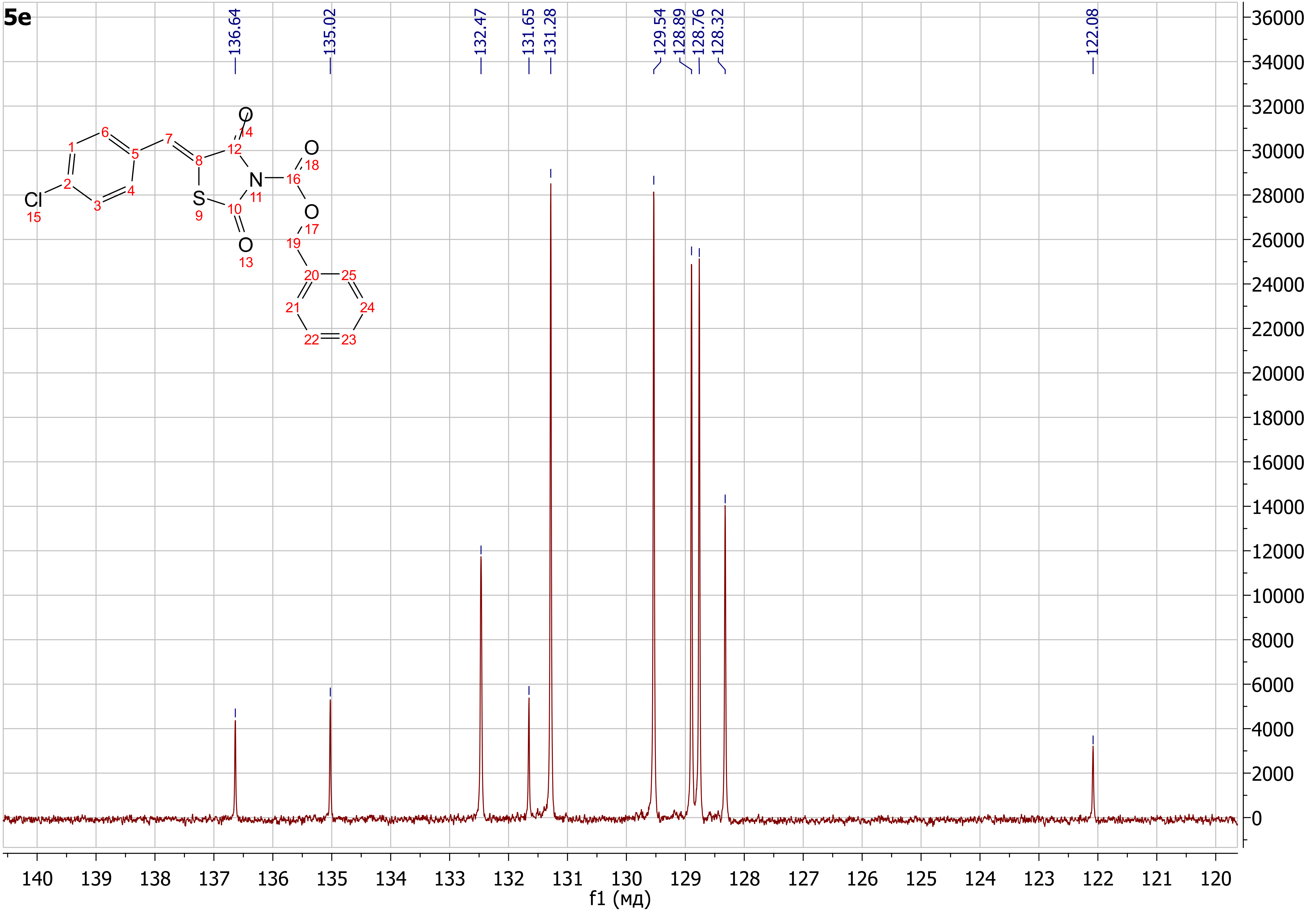

5e
136.64
135.02
132.47
131.65
131.28
129.54
128.89
128.76
128.32
122.08
Cl
O
N
S
f1 (мд)

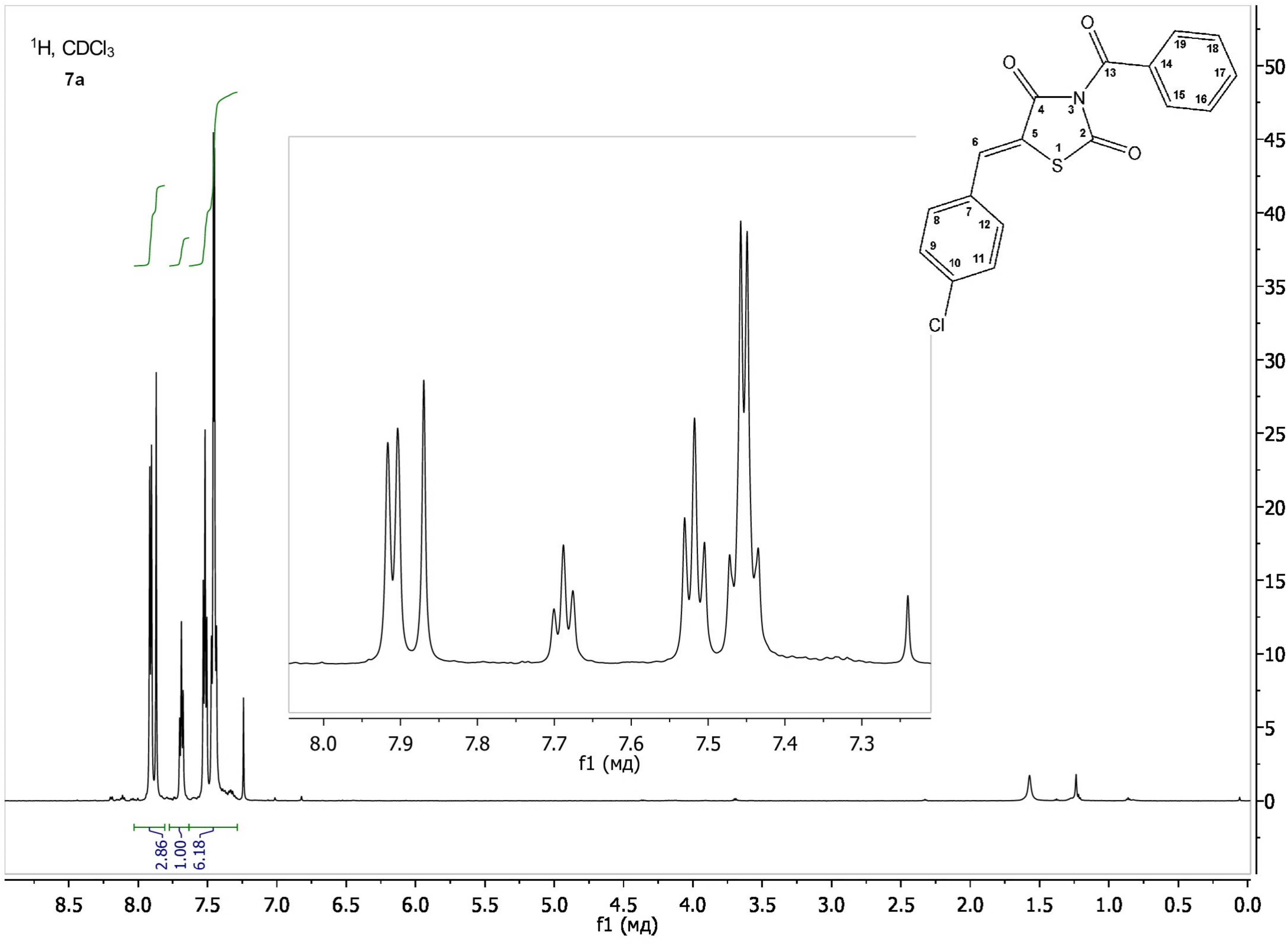

^{1}H, CDCl$_3$
7a
f1 (мд)
2.86
1.00
6.18

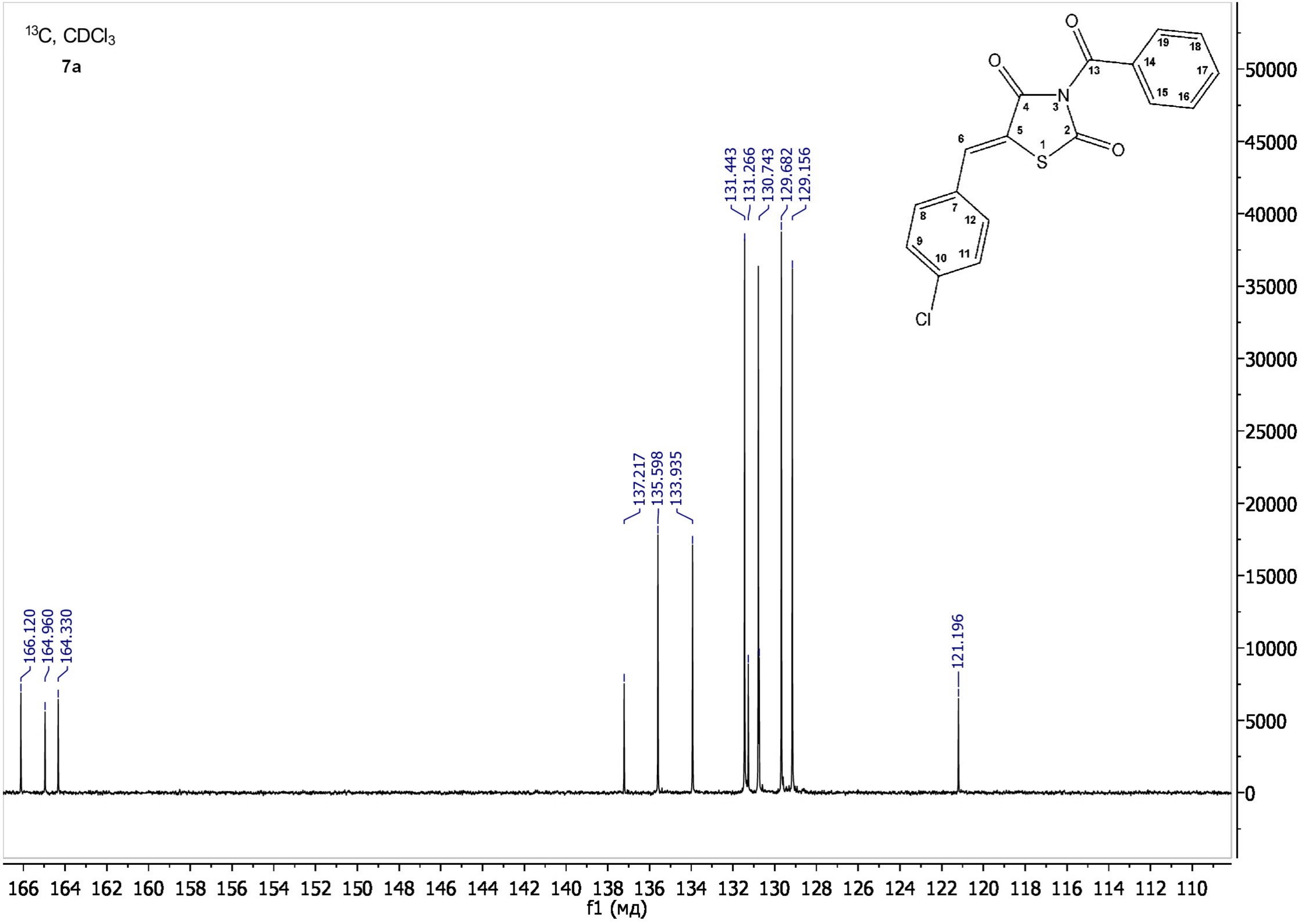

^{13}C, $CDCl_3$
7a
166.120
164.960
164.330
137.217
135.598
133.935
131.443
131.266
130.743
129.682
129.156
121.196
O
O
N
O
S
Cl
1
2
3
4
5
6
7
8
9
10
11
12
13
14
15
16
17
18
19
50000
45000
40000
35000
30000
25000
20000
15000
10000
5000
0
166 164 162 160 158 156 154 152 150 148 146 144 142 140 138 136 134 132 130 128 126 124 122 120 118 116 114 112 110
f1 (мд)

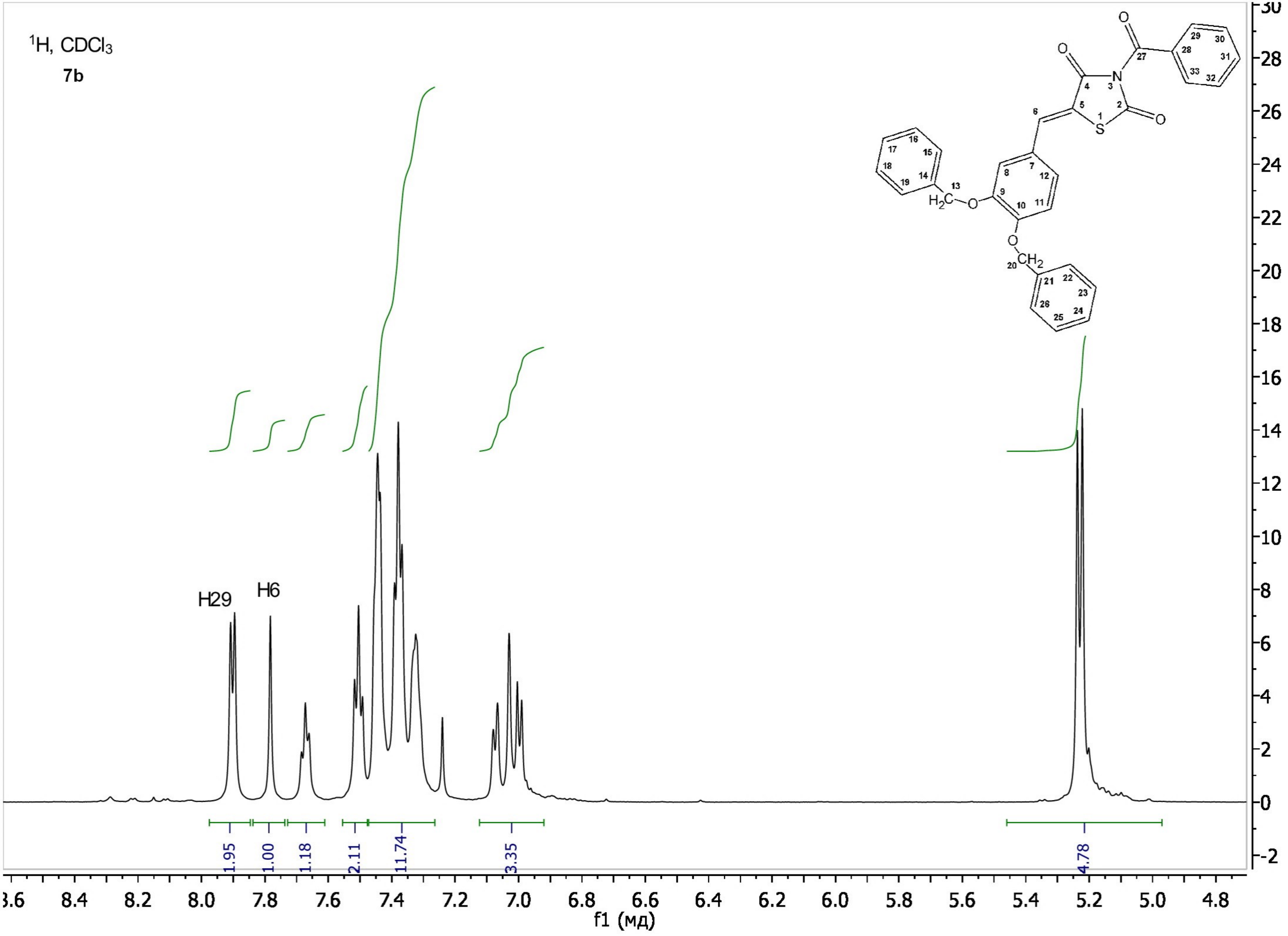

¹H, CDCl3
7b
H29
H6
1.95
1.00
1.18
2.11
11.74
3.35
4.78
f1 (мд)

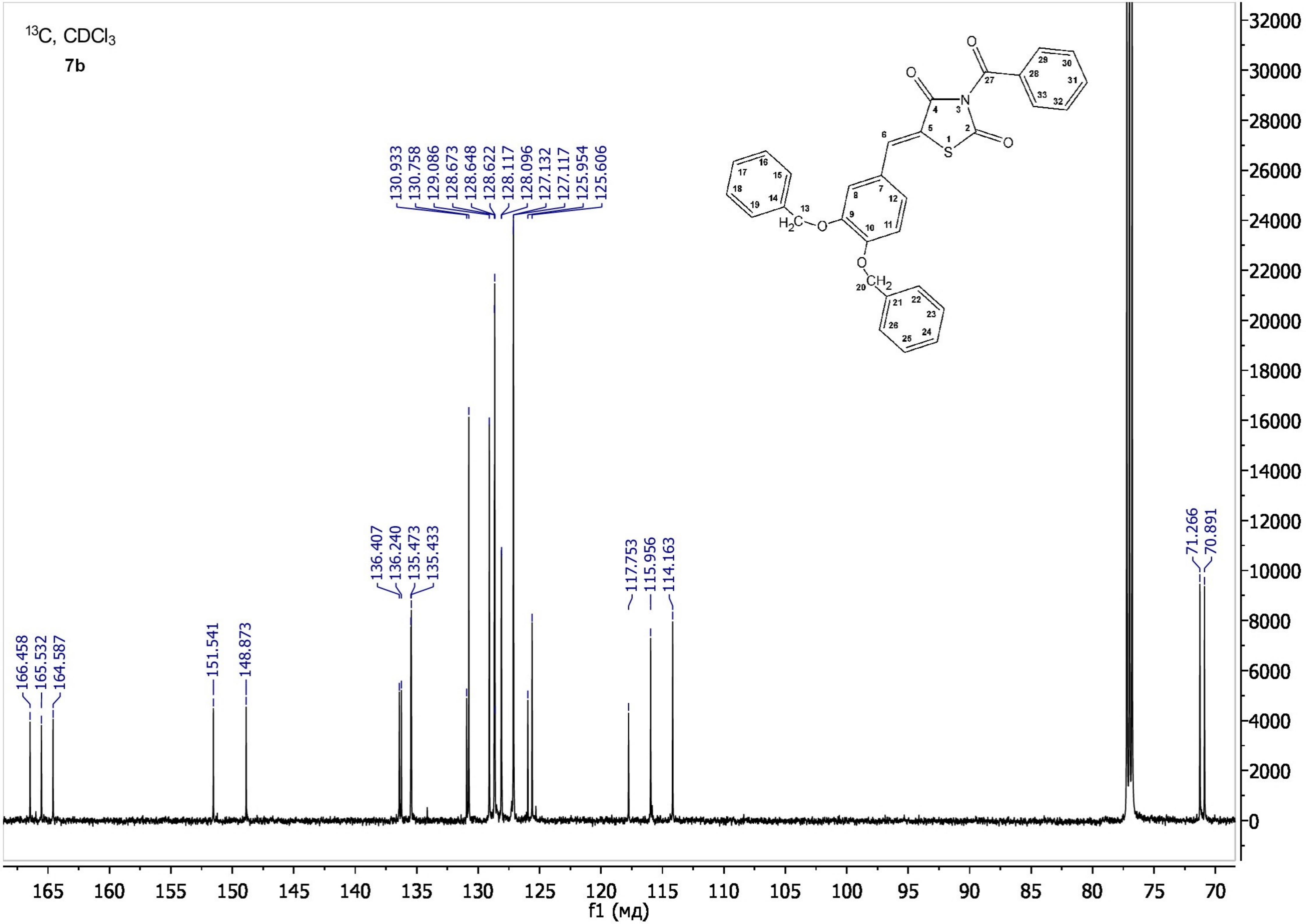

13C, CDCl3
7b
166.458
165.532
164.587
151.541
148.873
136.407
136.240
135.473
135.433
130.933
130.758
129.086
128.673
128.648
128.622
128.117
128.096
127.132
127.117
125.954
125.606
117.753
115.956
114.163
71.266
70.891
f1 (мд)

8

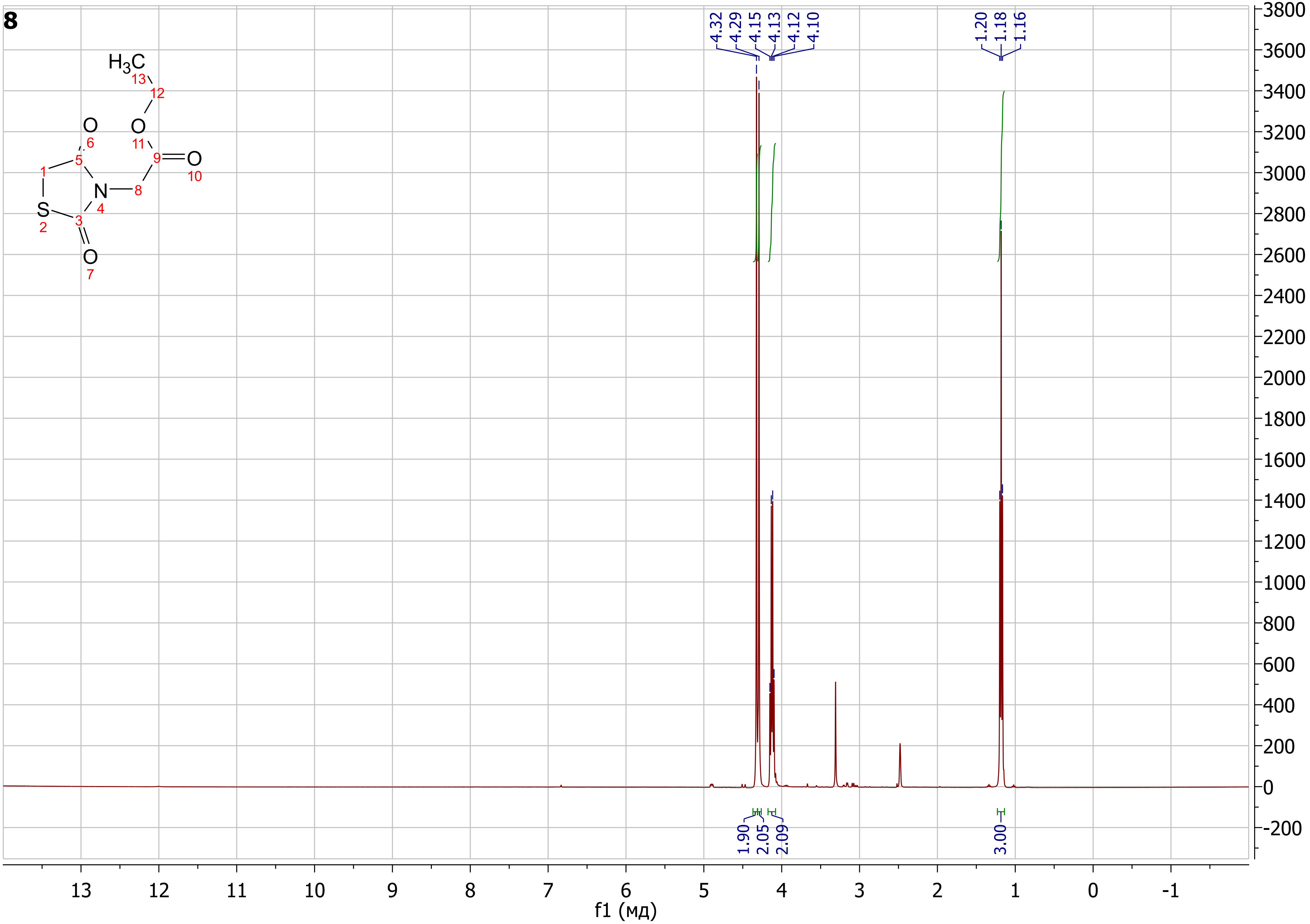

H3C
O
O
O
N
S
O
1
2
3
4
5
6
7
8
9
10
11
12
13
4.32
4.29
4.15
4.13
4.12
4.10
1.20
1.18
1.16
1.90
2.05
2.09
3.00
f1 (мд)

8

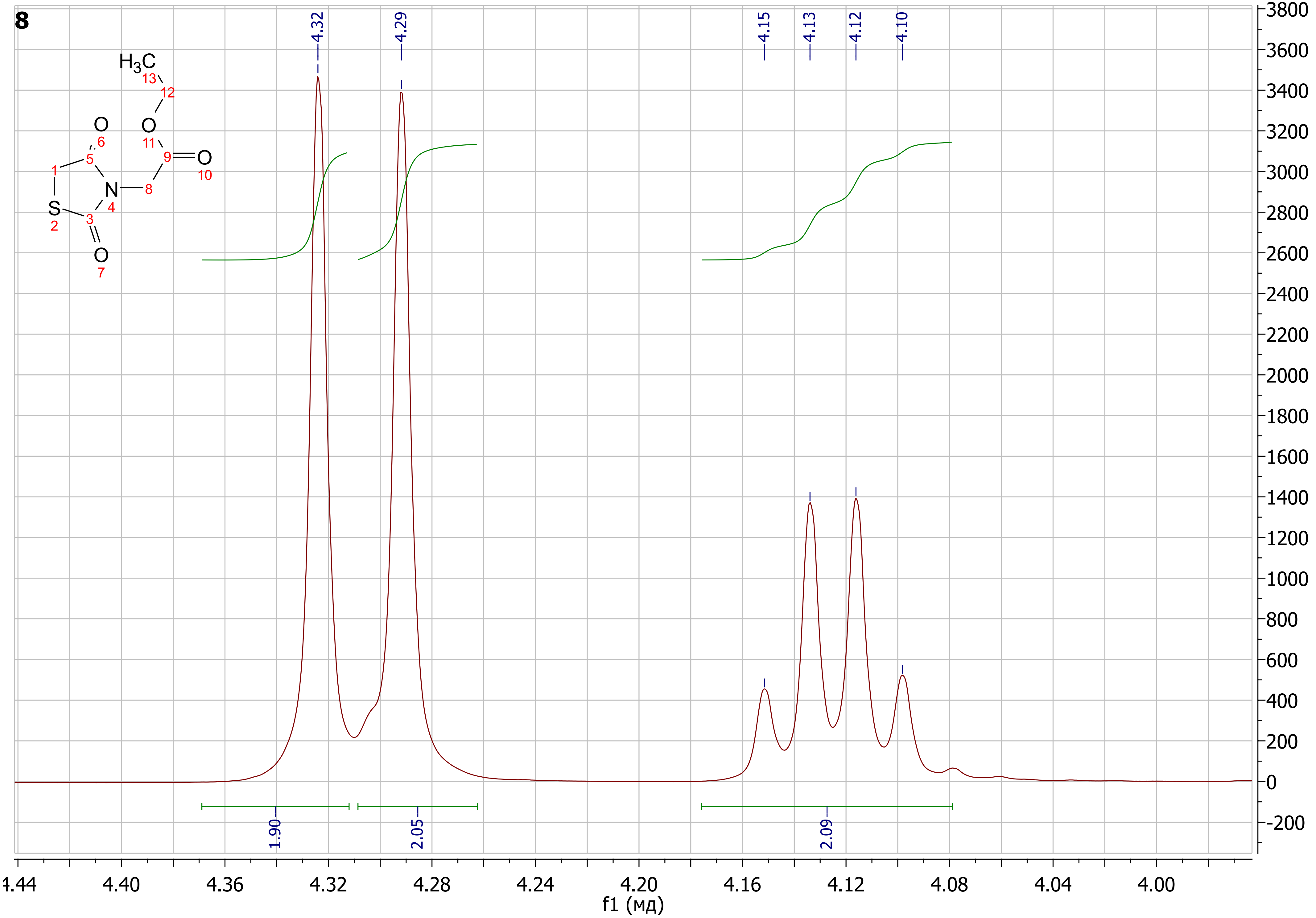

H3C
O
O
O
N
S
O
4.32
4.29
4.15
4.13
4.12
4.10
1.90
2.05
2.09
4.44
4.40
4.36
4.32
4.28
4.24
4.20
4.16
4.12
4.08
4.04
4.00
f1 (мд)
3800
3600
3400
3200
3000
2800
2600
2400
2200
2000
1800
1600
1400
1200
1000
800
600
400
200
0
-200

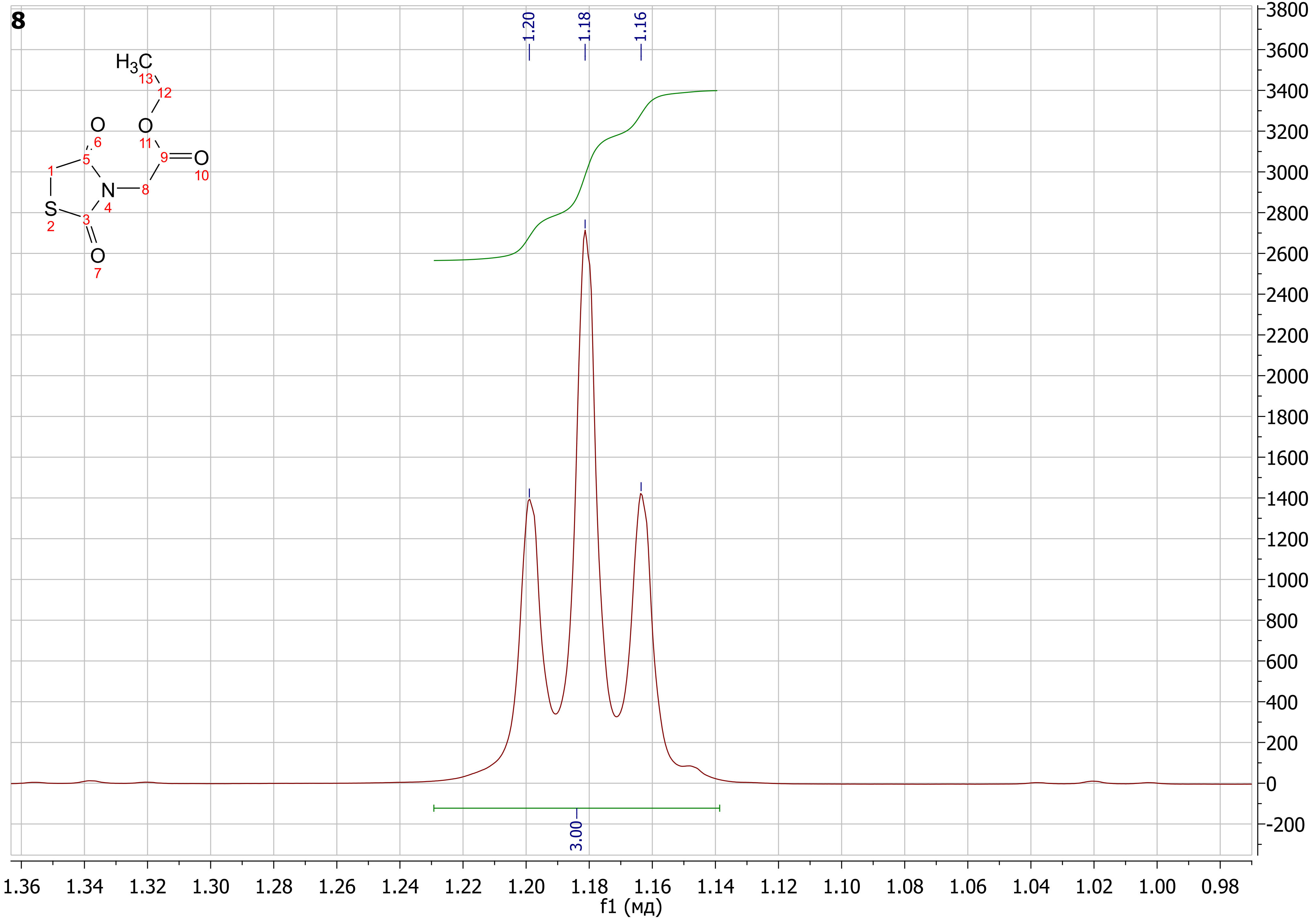

8
H3C
13
12
O
11
O
6
5
1
9
O
10
N
4
8
S
2
3
O
7
1.20
1.18
1.16
3.00
f1 (мд)

8

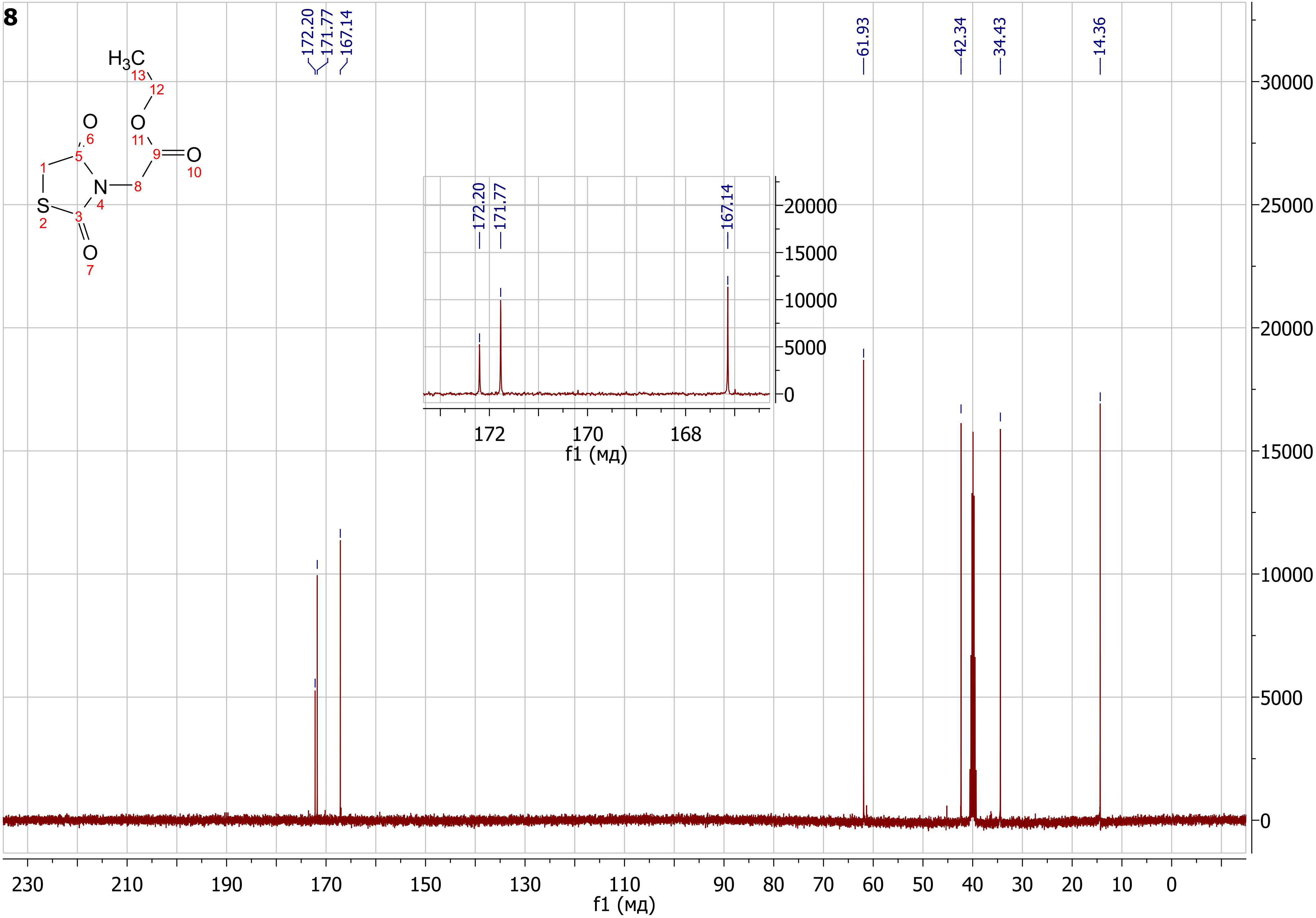

9

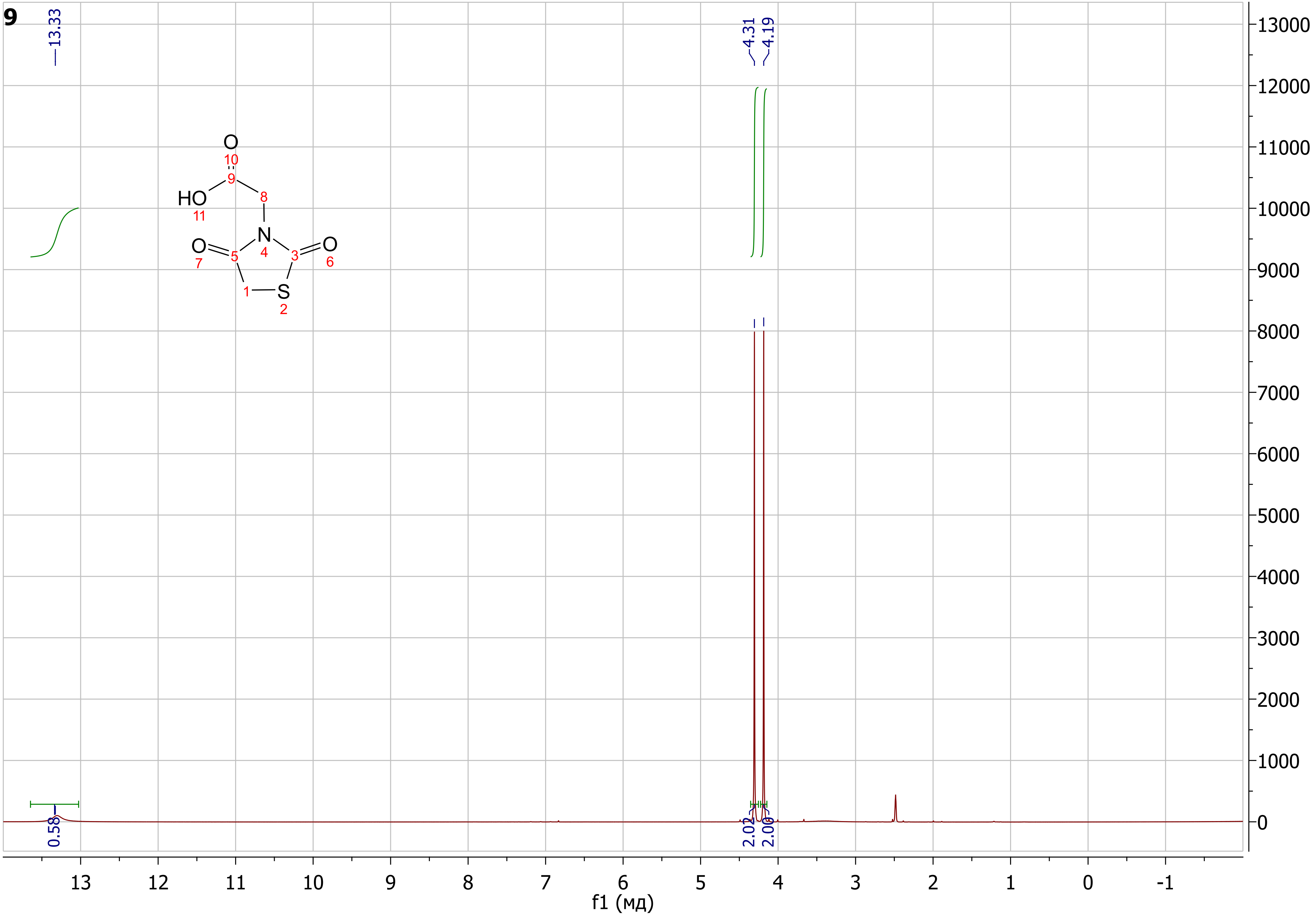

13.33
4.31
4.19
HO
O
N
S
0.58
2.02
2.00
13 12 11 10 9 8 7 6 5 4 3 2 1 0 -1
f1 (мд)
13000 12000 11000 10000 9000 8000 7000 6000 5000 4000 3000 2000 1000 0

9

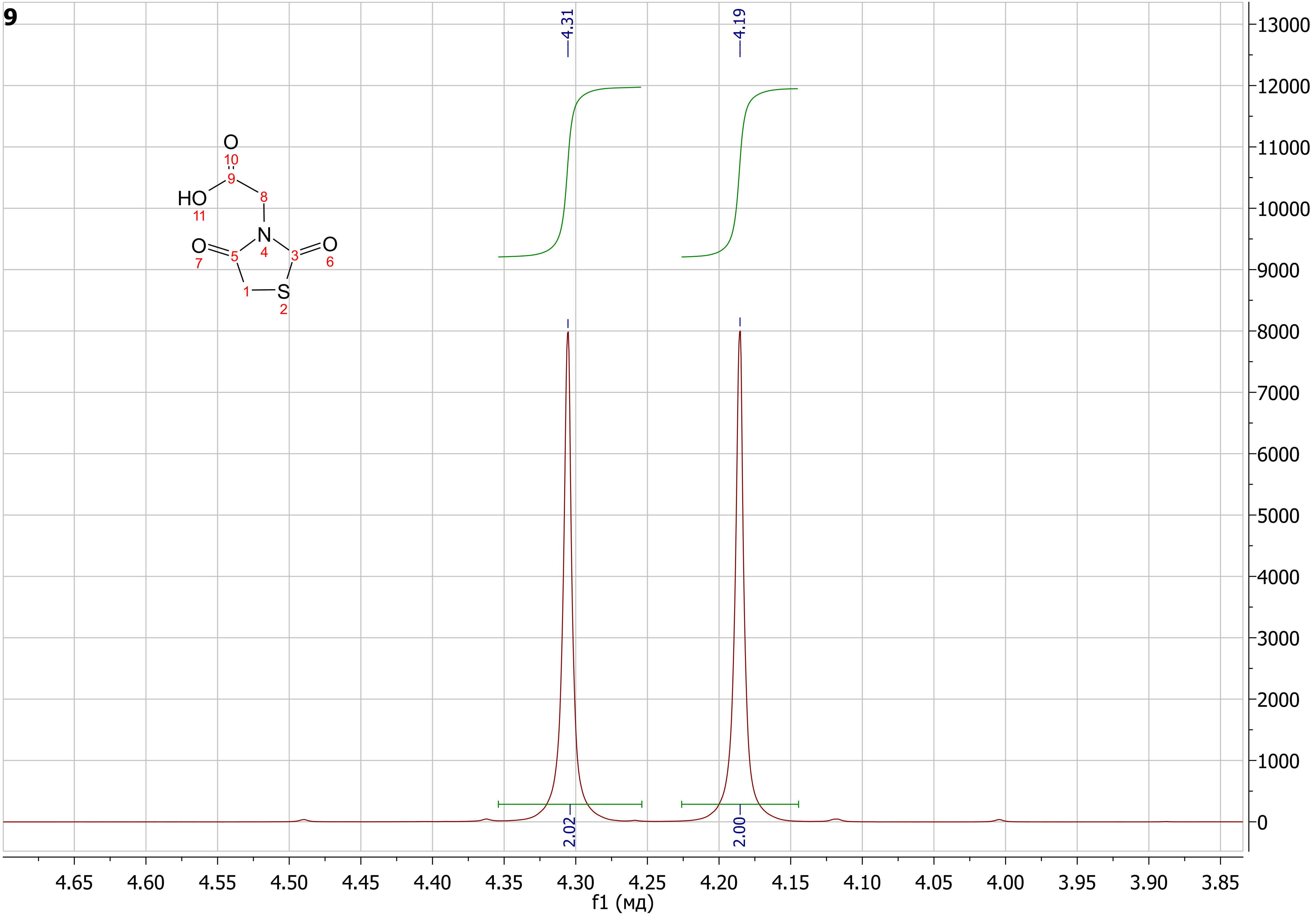

4.31
4.19
2.02
2.00
HO
O
N
S
f1 (мд)
4.65 4.60 4.55 4.50 4.45 4.40 4.35 4.30 4.25 4.20 4.15 4.10 4.05 4.00 3.95 3.90 3.85
13000 12000 11000 10000 9000 8000 7000 6000 5000 4000 3000 2000 1000 0

9

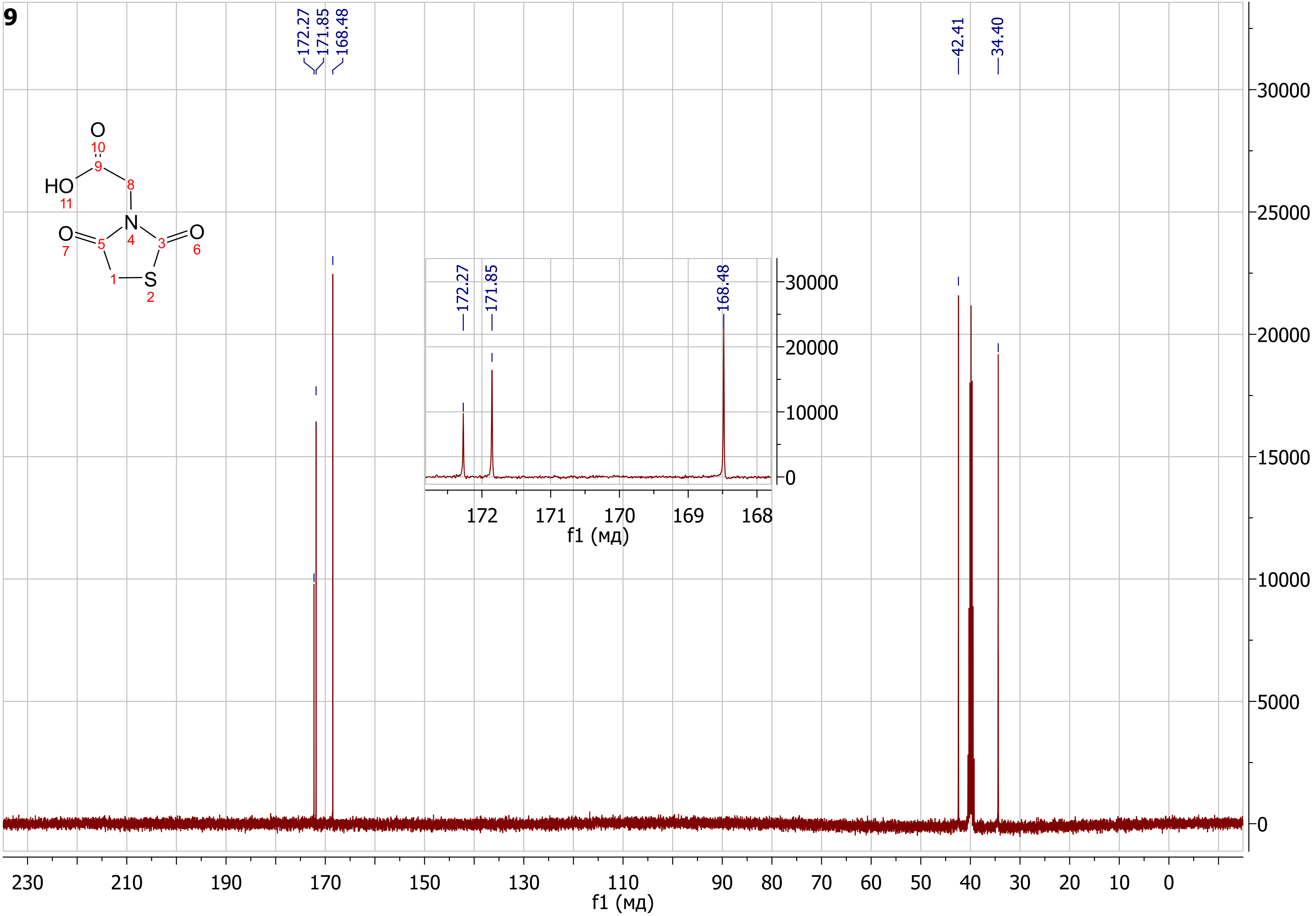

172.27
171.85
168.48
42.41
34.40
O
10
9
HO
11
8
N
4
O
7
5
3
O
6
1
S
2
30000
25000
20000
15000
10000
5000
0
230
210
190
170
150
130
110
90
80
70
60
50
40
30
20
10
0
f1 (мд)
172
171
170
169
168
f1 (мд)

10a

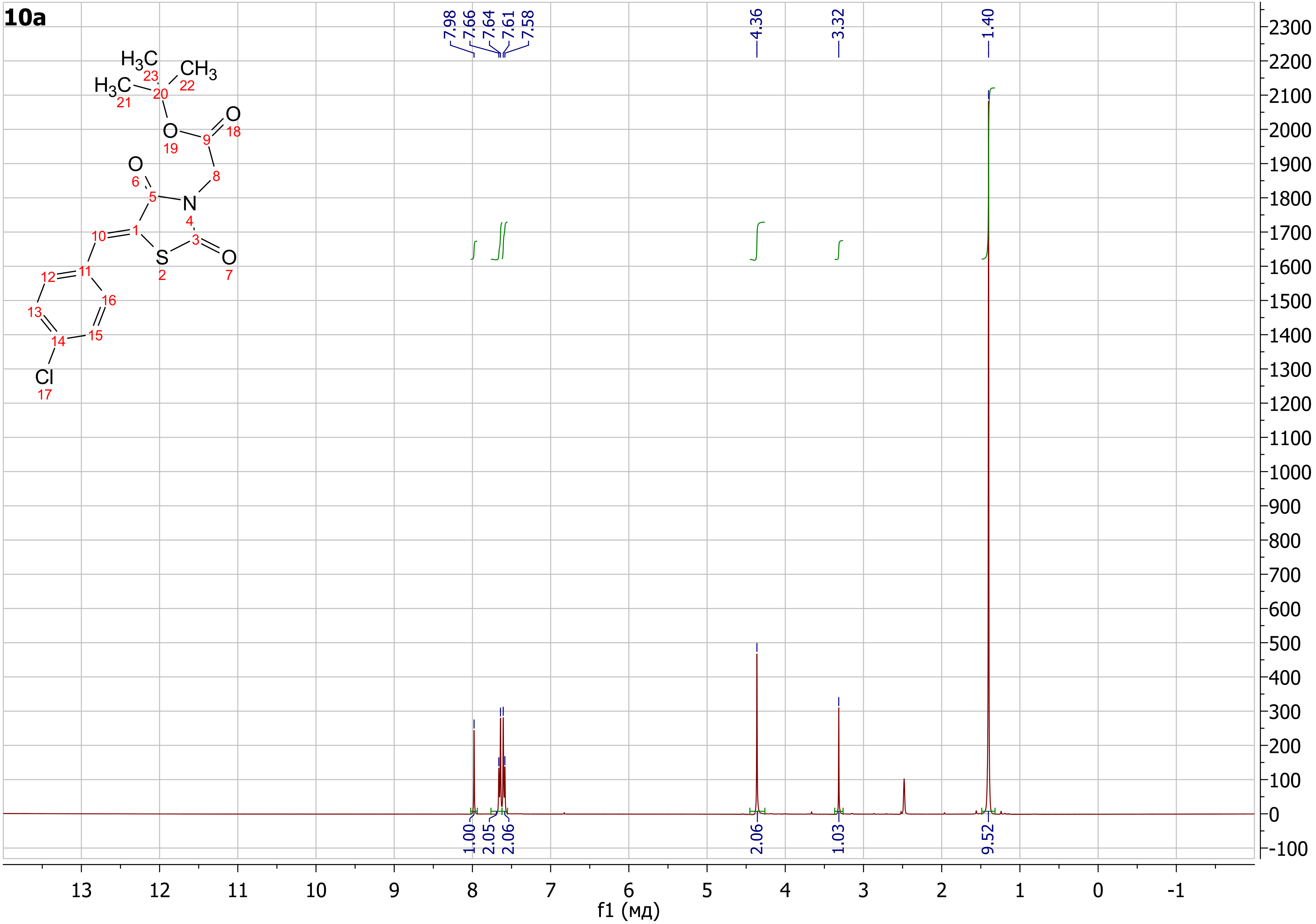

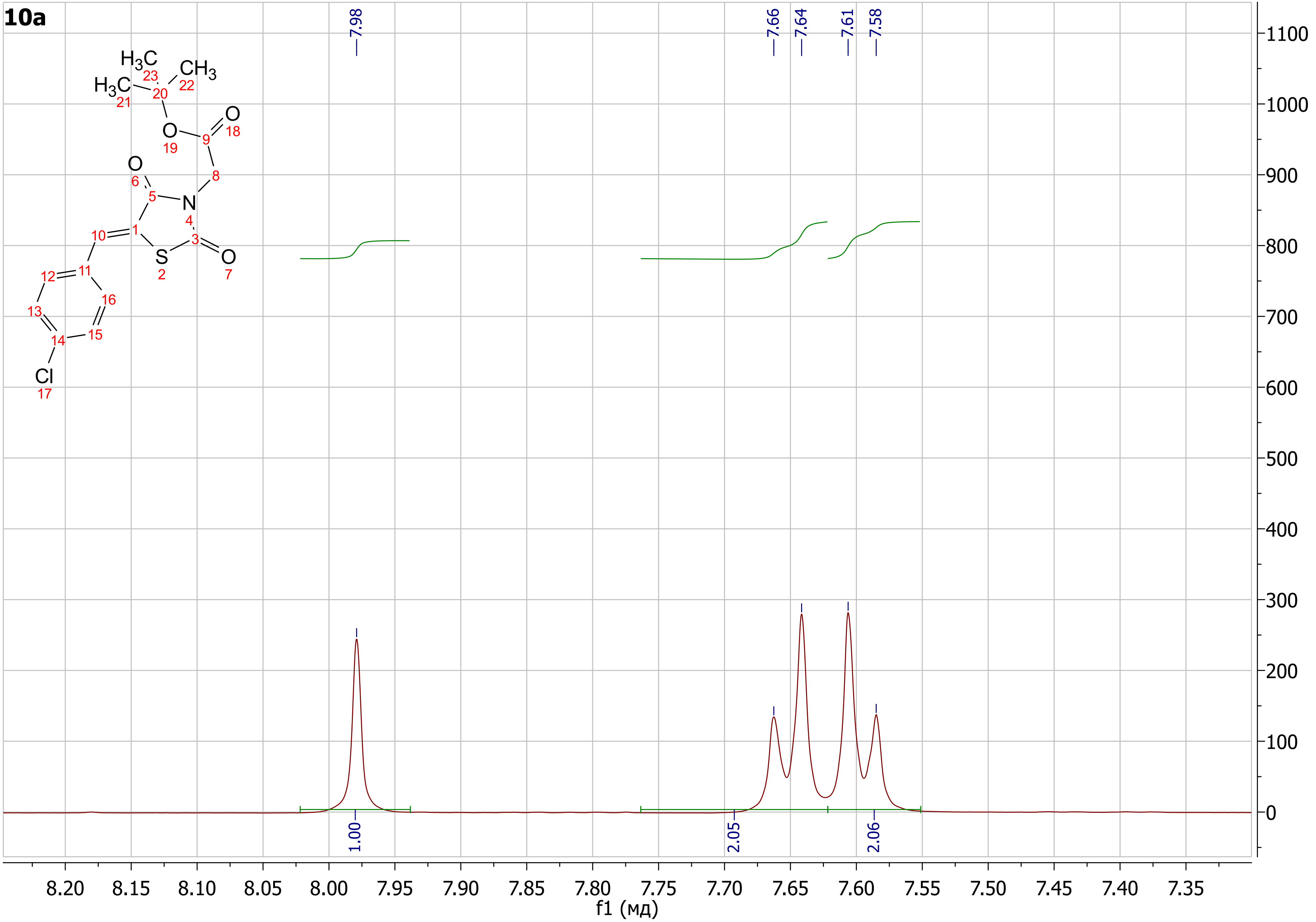

10a
7.98
7.66
7.64
7.61
7.58
1.00
2.05
2.06
H3C
CH3
O
N
S
Cl
8.20
8.15
8.10
8.05
8.00
7.95
7.90
7.85
7.80
7.75
7.70
7.65
7.60
7.55
7.50
7.45
7.40
7.35
f1 (мд)
1100
1000
900
800
700
600
500
400
300
200
100
0

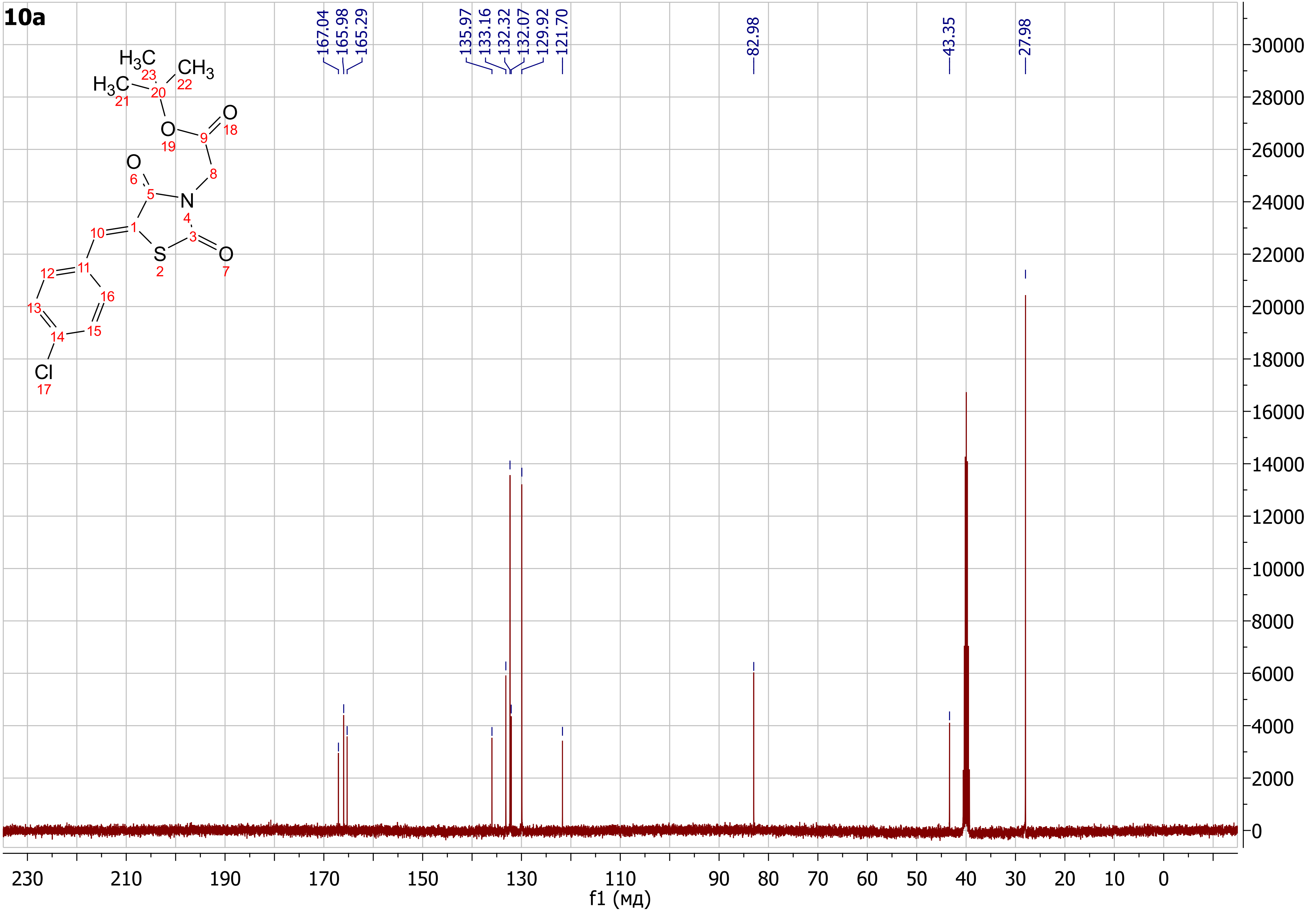

10a
167.04
165.98
165.29
135.97
133.16
132.32
132.07
129.92
121.70
82.98
43.35
27.98
f1 (мд)

10a

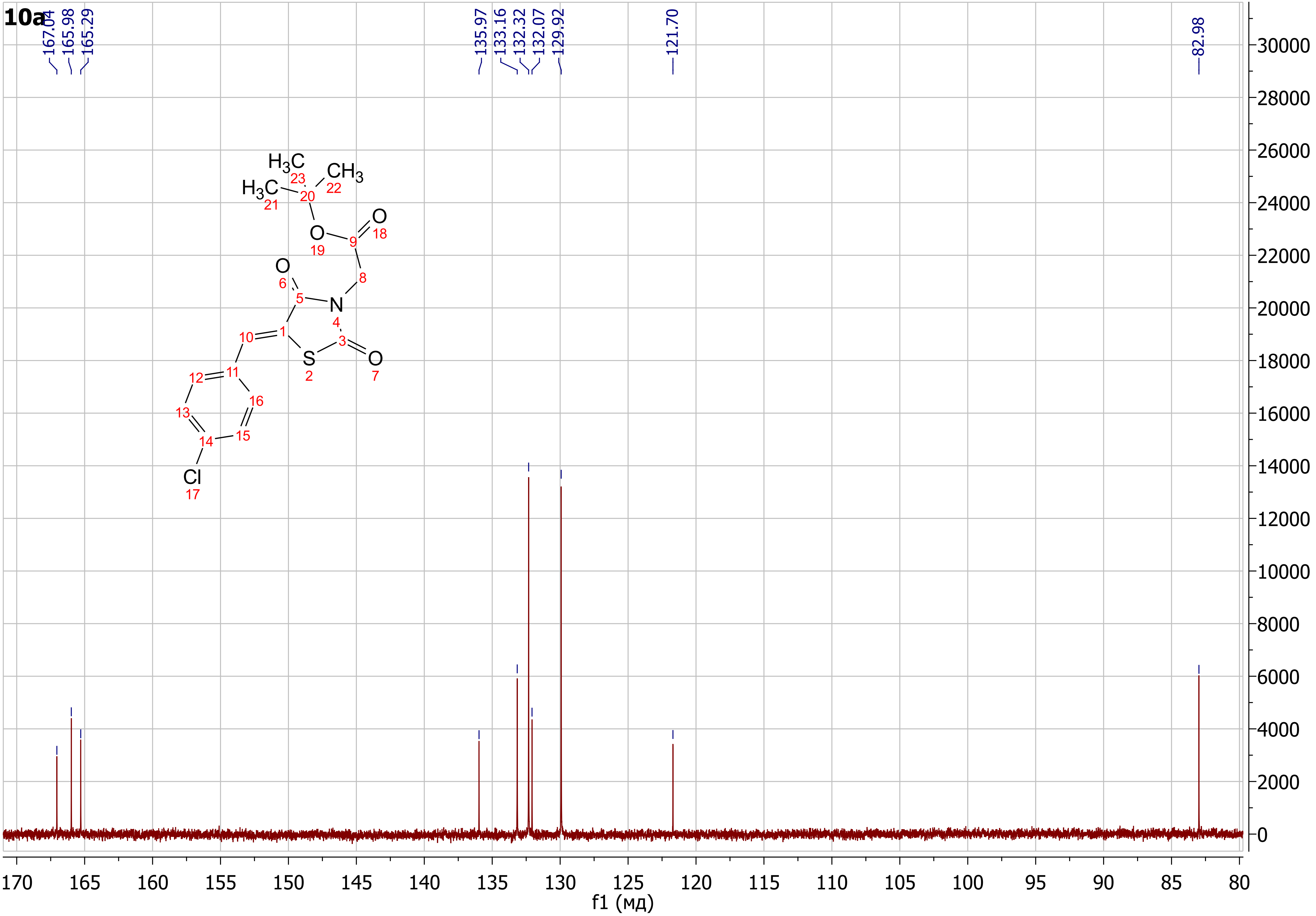

167.04
165.98
165.29
135.97
133.16
132.32
132.07
129.92
121.70
82.98
H3C
CH3
H3C
O
O
O
N
S
O
Cl
1
2
3
4
5
6
7
8
9
10
11
12
13
14
15
16
17
18
19
20
21
22
23
30000
28000
26000
24000
22000
20000
18000
16000
14000
12000
10000
8000
6000
4000
2000
0
170
165
160
155
150
145
140
135
130
125
120
115
110
105
100
95
90
85
80
f1 (мд)

10a

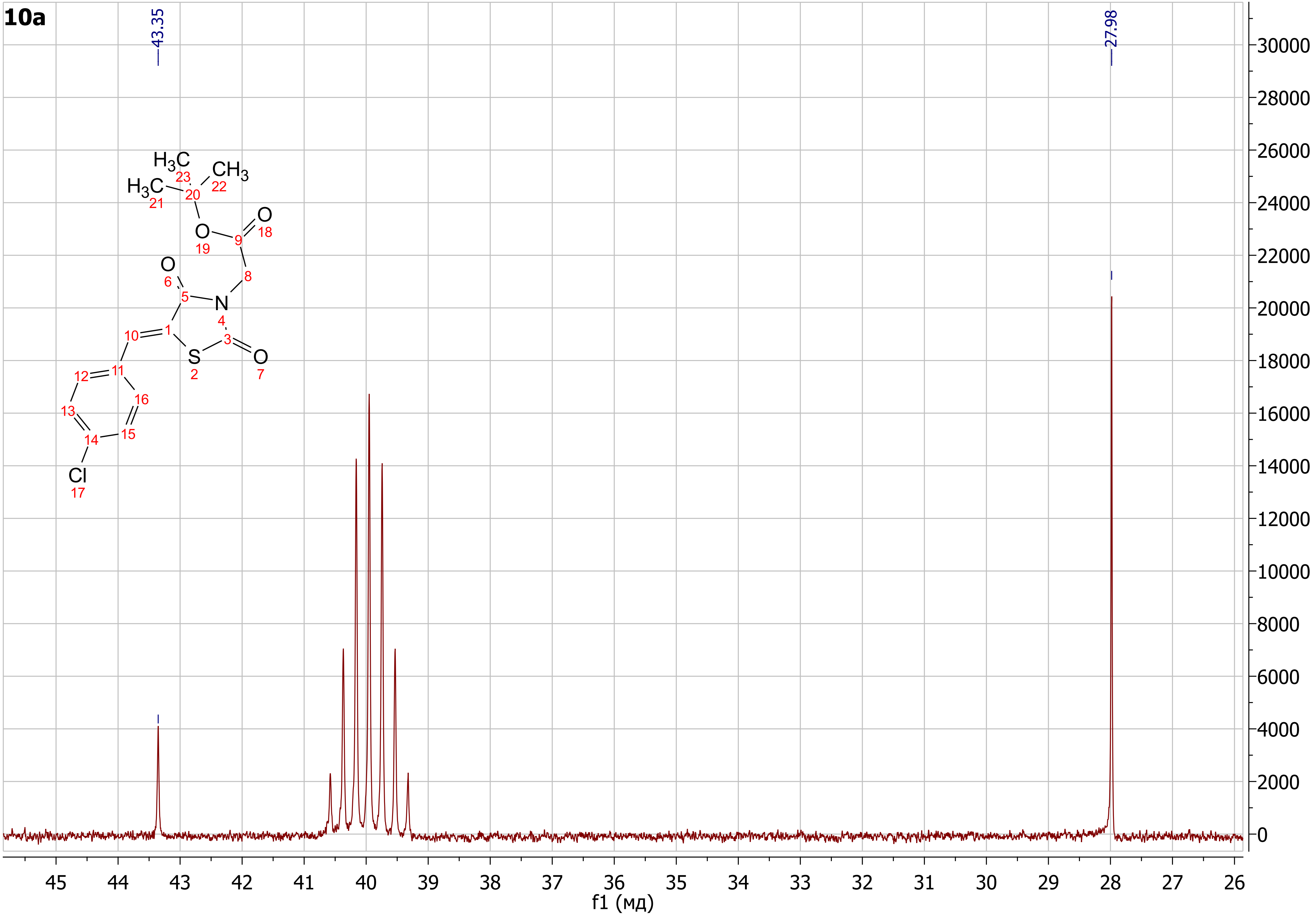

43.35
27.98
H3C
CH3
H3C
O
O
O
N
S
O
Cl
1
2
3
4
5
6
7
8
9
10
11
12
13
14
15
16
17
18
19
20
21
22
23
30000
28000
26000
24000
22000
20000
18000
16000
14000
12000
10000
8000
6000
4000
2000
0
45
44
43
42
41
40
39
38
37
36
35
34
33
32
31
30
29
28
27
26
f1 (мд)

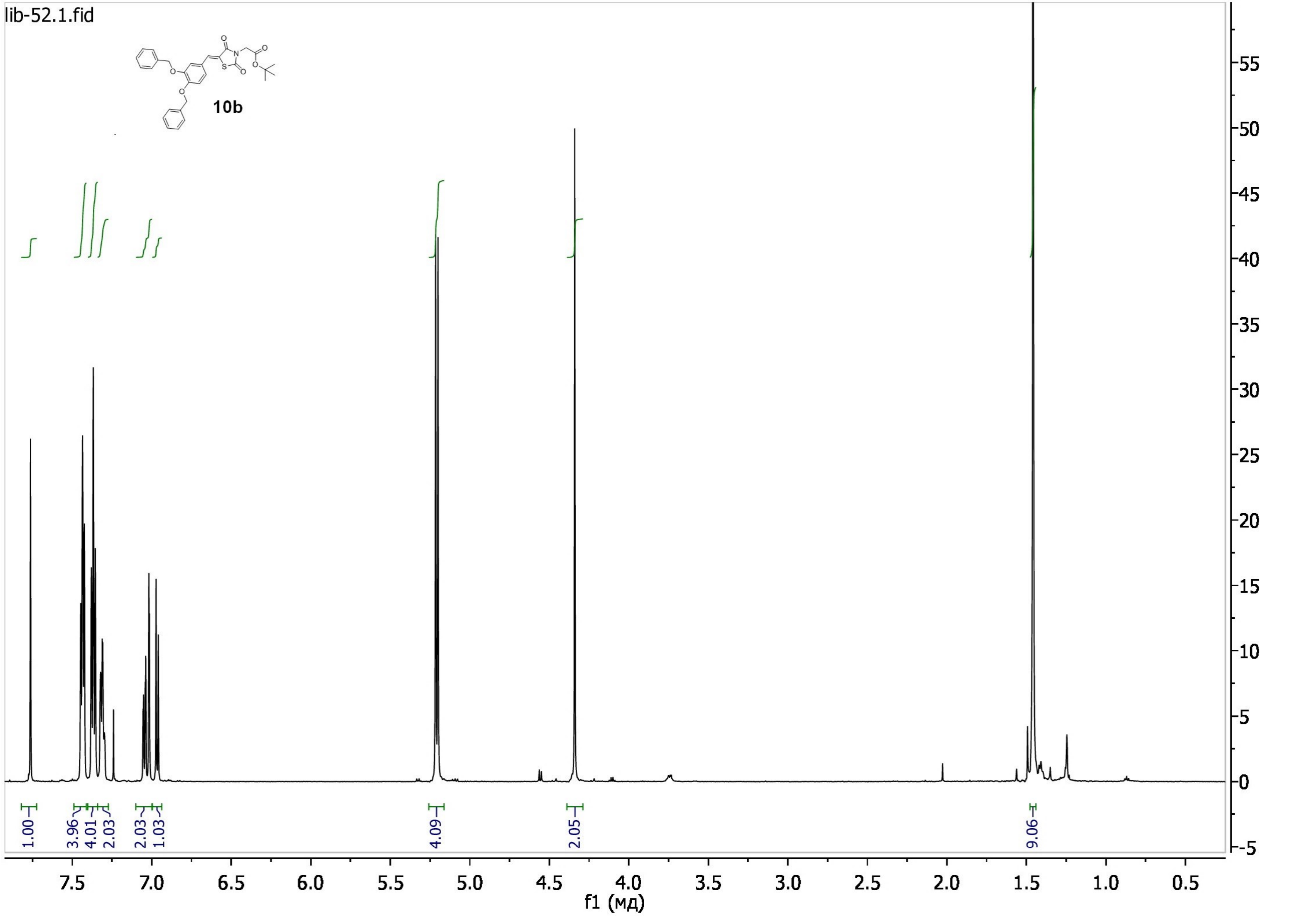
lib-52.1.fid
10b
1.00
3.96
4.01
2.03
2.03
1.03
4.09
2.05
9.06
f1 (мд)

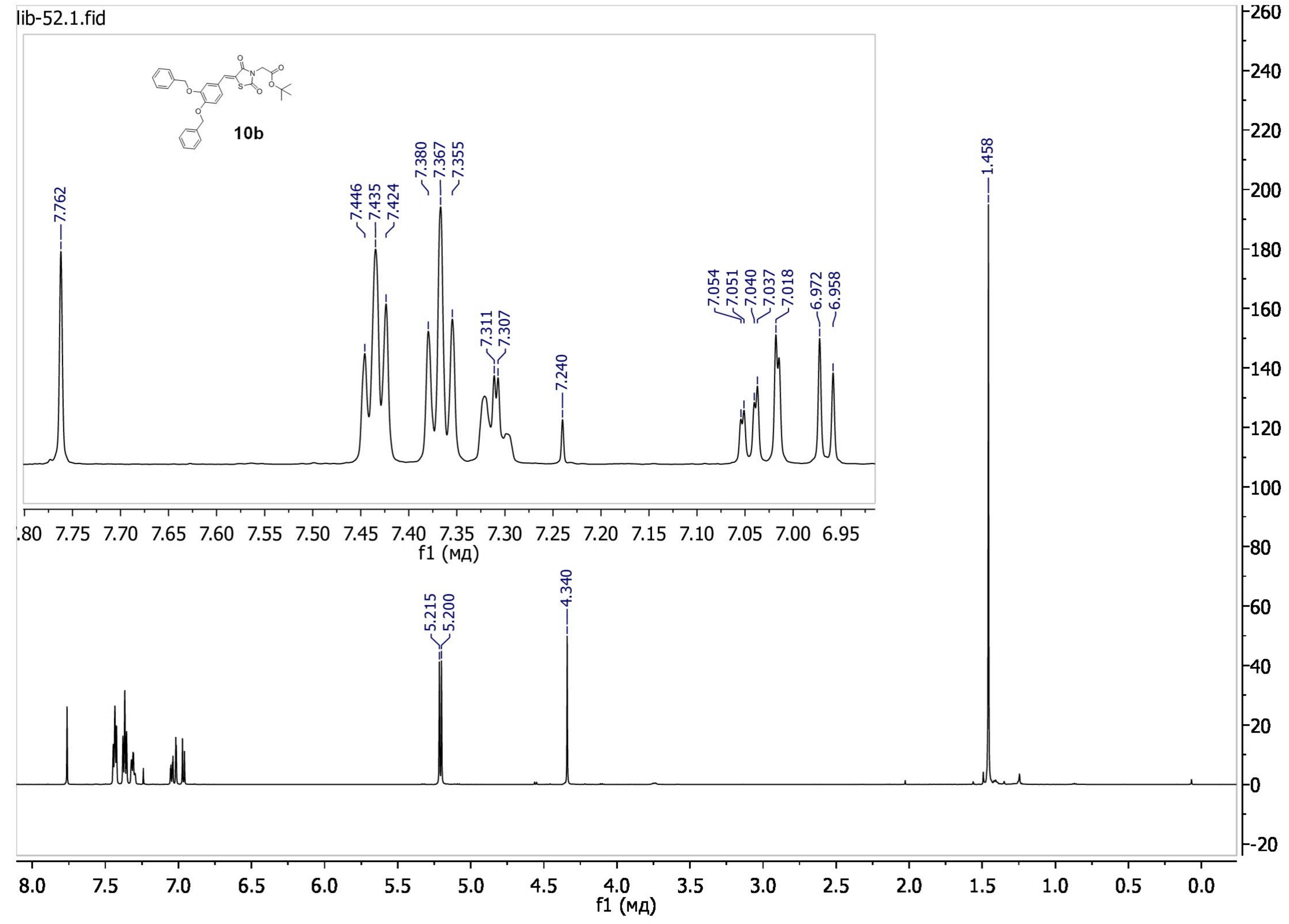

lib-52.1.fid
10b
7.762
7.446
7.435
7.424
7.380
7.367
7.355
7.311
7.307
7.240
7.054
7.051
7.040
7.037
7.018
6.972
6.958
.80 7.75 7.70 7.65 7.60 7.55 7.50 7.45 7.40 7.35 7.30 7.25 7.20 7.15 7.10 7.05 7.00 6.95
f1 (мд)
5.215
5.200
4.340
1.458
8.0 7.5 7.0 6.5 6.0 5.5 5.0 4.5 4.0 3.5 3.0 2.5 2.0 1.5 1.0 0.5 0.0
f1 (мд)
260 240 220 200 180 160 140 120 100 80 60 40 20 0 -20

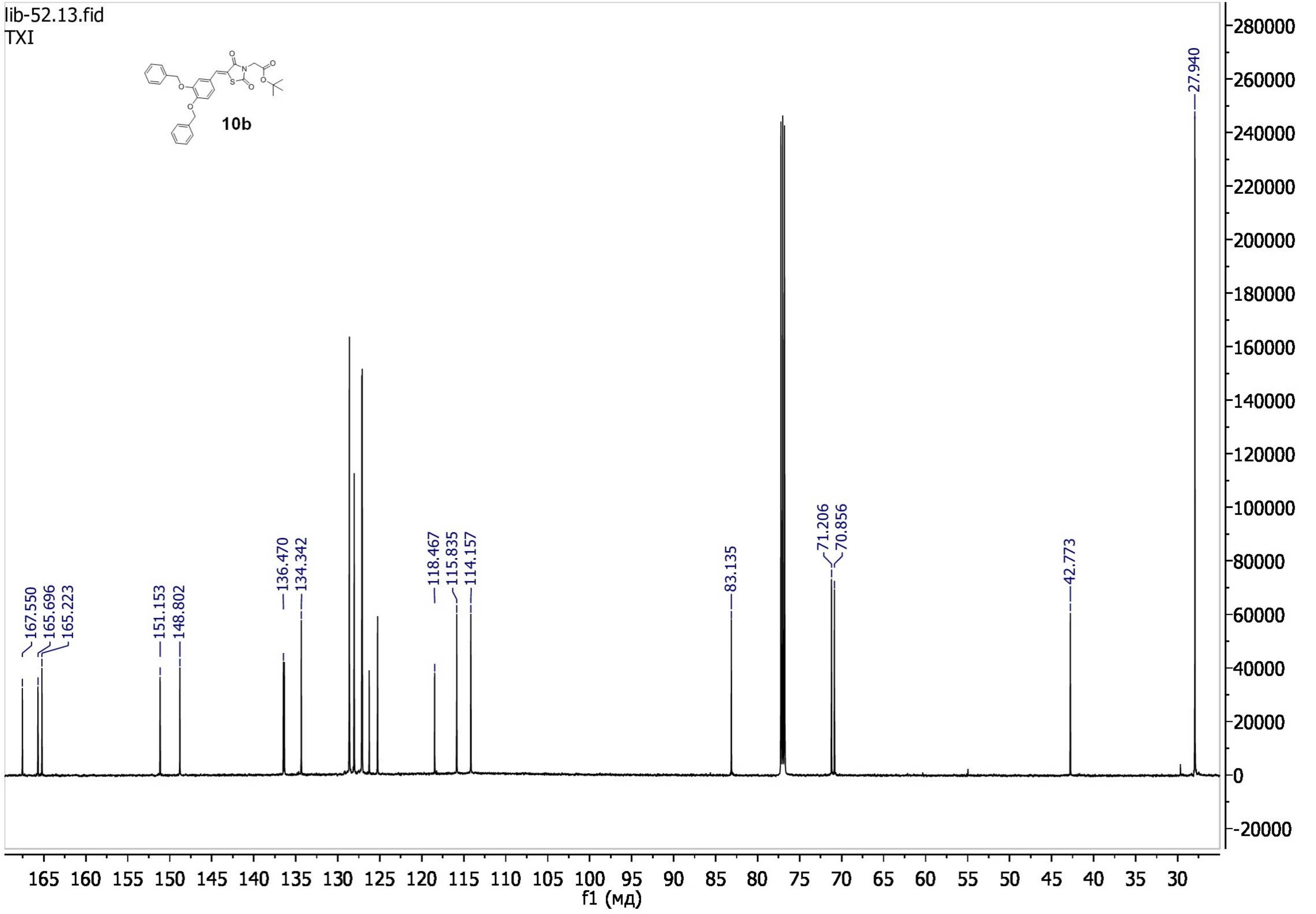
lib-52.13.fid
TXI
10b
167.550
165.696
165.223
151.153
148.802
136.470
134.342
118.467
115.835
114.157
83.135
71.206
70.856
42.773
27.940
f1 (мд)

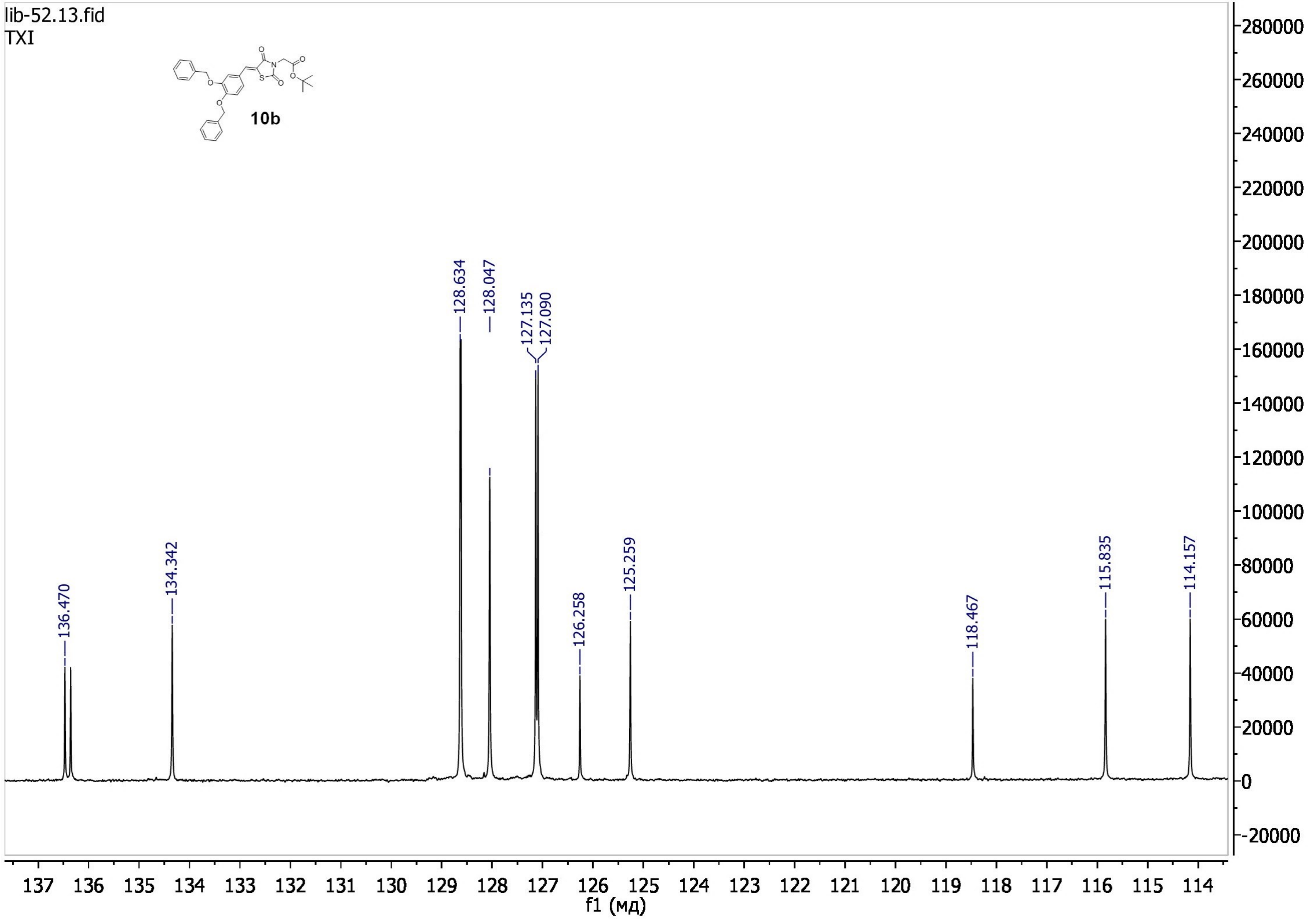
lib-52.13.fid
TXI
10b
136.470
134.342
128.634
128.047
127.135
127.090
126.258
125.259
118.467
115.835
114.157
280000
260000
240000
220000
200000
180000
160000
140000
120000
100000
80000
60000
40000
20000
0
-20000
137 136 135 134 133 132 131 130 129 128 127 126 125 124 123 122 121 120 119 118 117 116 115 114
f1 (мд)

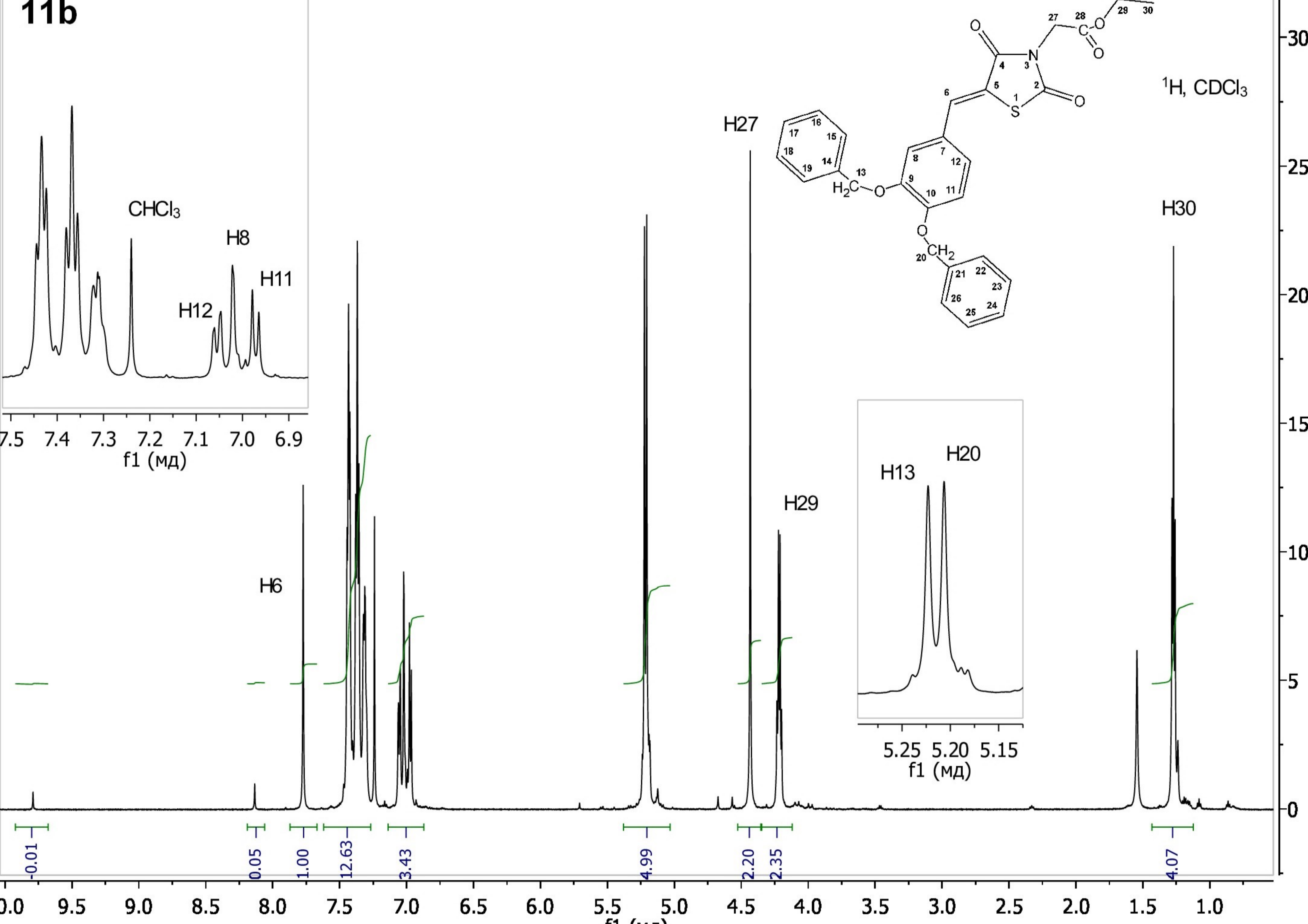

11b
¹H, CDCl₃
CHCl₃
H8
H11
H12
H6
H27
H29
H30
H13
H20
f1 (мд)
-0.01
0.05
1.00
12.63
3.43
4.99
2.20
2.35
4.07

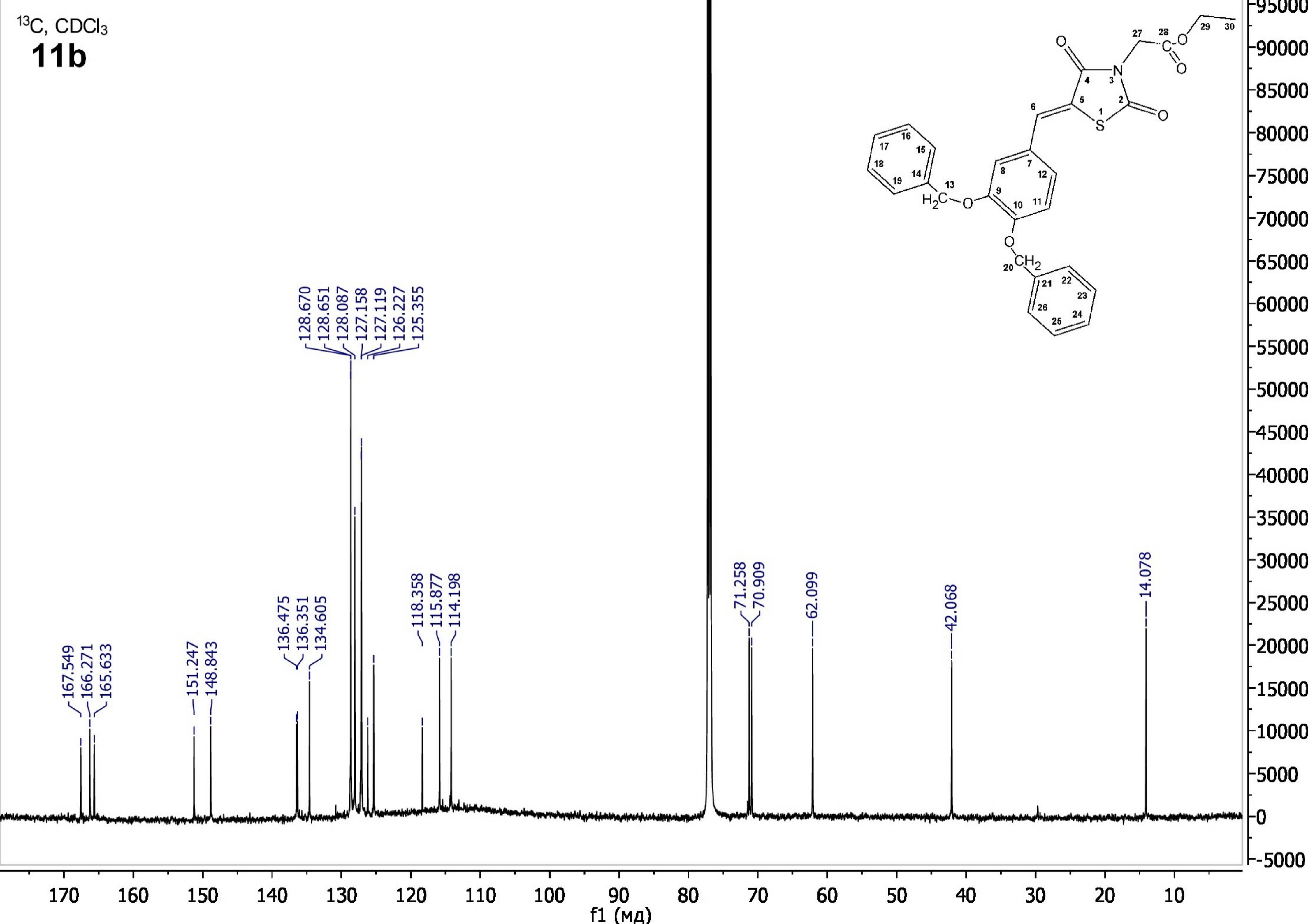

13C, CDCl3
11b
167.549
166.271
165.633
151.247
148.843
136.475
136.351
134.605
128.670
128.651
128.087
127.158
127.119
126.227
125.355
118.358
115.877
114.198
71.258
70.909
62.099
42.068
14.078
f1 (мд)

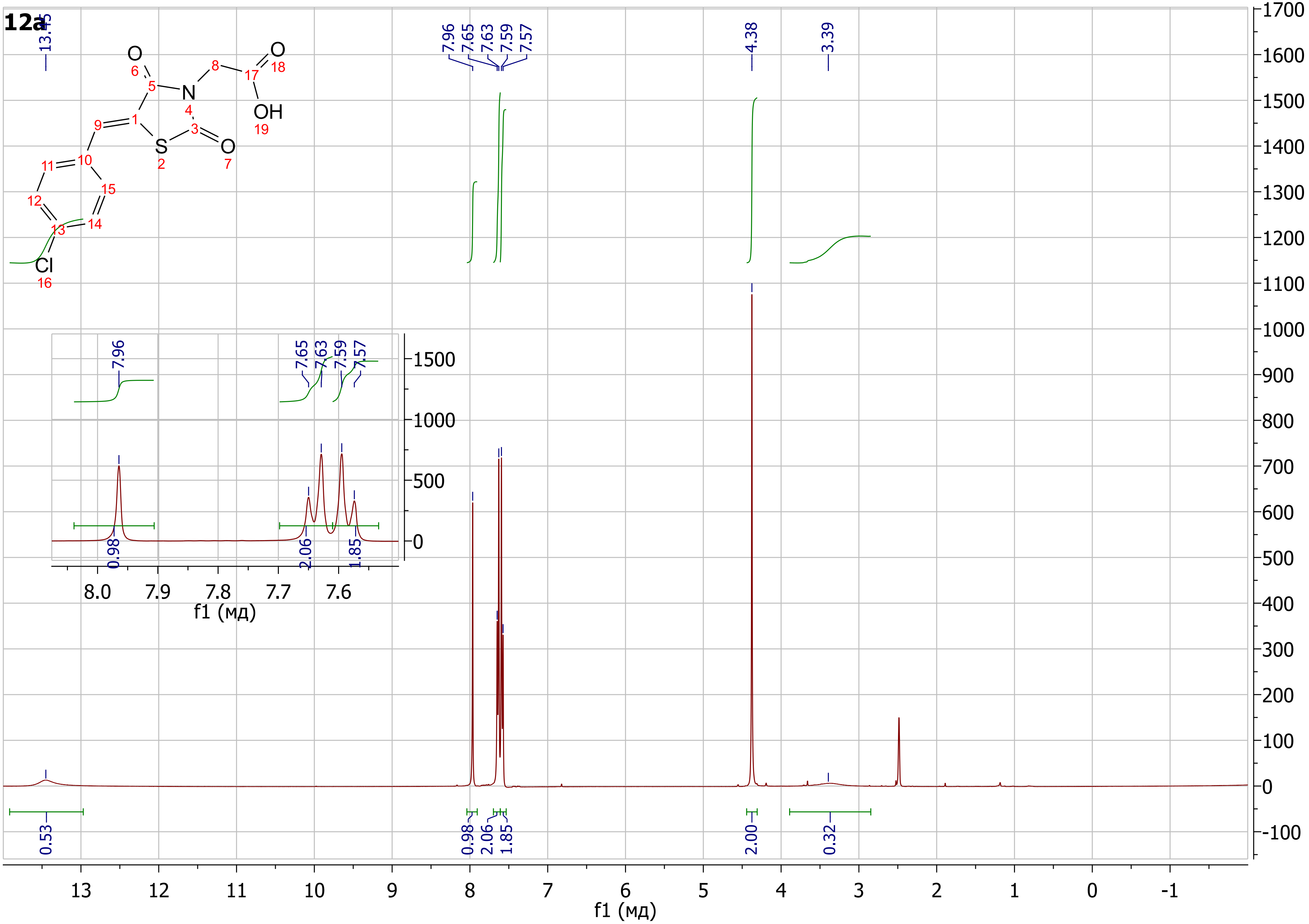

12a
13.45
7.96
7.65
7.63
7.59
7.57
4.38
3.39
0.53
0.98
2.06
1.85
2.00
0.32
f1 (мд)

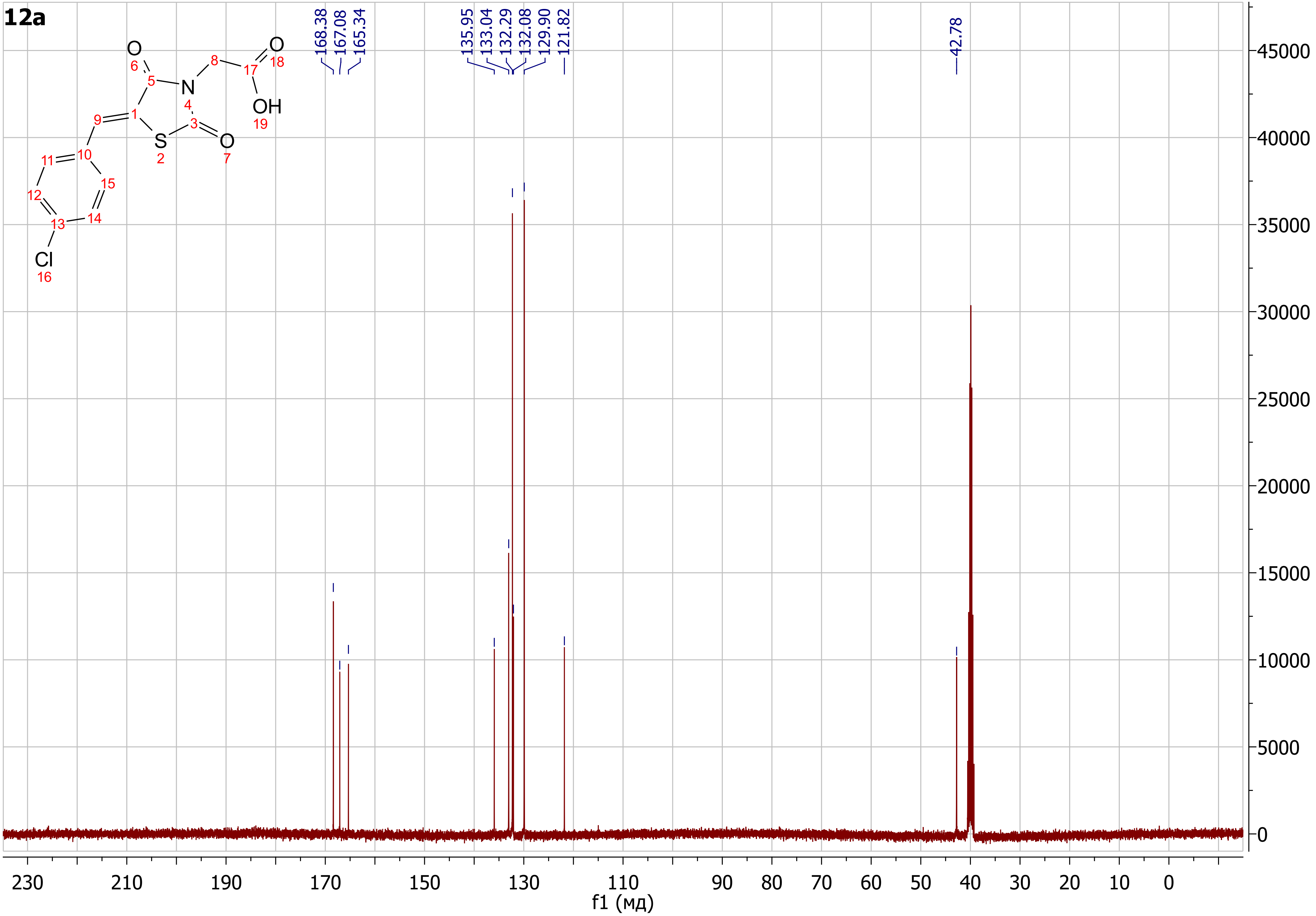

12a
O
6
5
N
4
8
17
O
18
OH
19
9
1
3
S
2
O
7
11
10
15
12
13
14
Cl
16
168.38
167.08
165.34
135.95
133.04
132.29
132.08
129.90
121.82
42.78
45000
40000
35000
30000
25000
20000
15000
10000
5000
0
230
210
190
170
150
130
110
90
80
70
60
50
40
30
20
10
0
f1 (мд)

lib-53.1.fid

^{1}H NMR (600 MHz, Chloroform-*d*) δ 7.78 (s, 1H), 7.46 – 7.41 (m, 5H), 7.37 (td, *J* = 7.6, 2.5 Hz, 5H), 7.31 (td, *J* = 7.3, 4.6 Hz, 2H), 7.05 (dd, *J* = 8.4, 2.0 Hz, 1H), 7.01 (d, *J* = 1.9 Hz, 1H), 6.97 (d, *J* = 8.4 Hz, 1H), 5.22 (s, 2H), 5.20 (s, 2H), 4.49 (s, 2H).

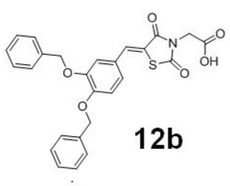

12b

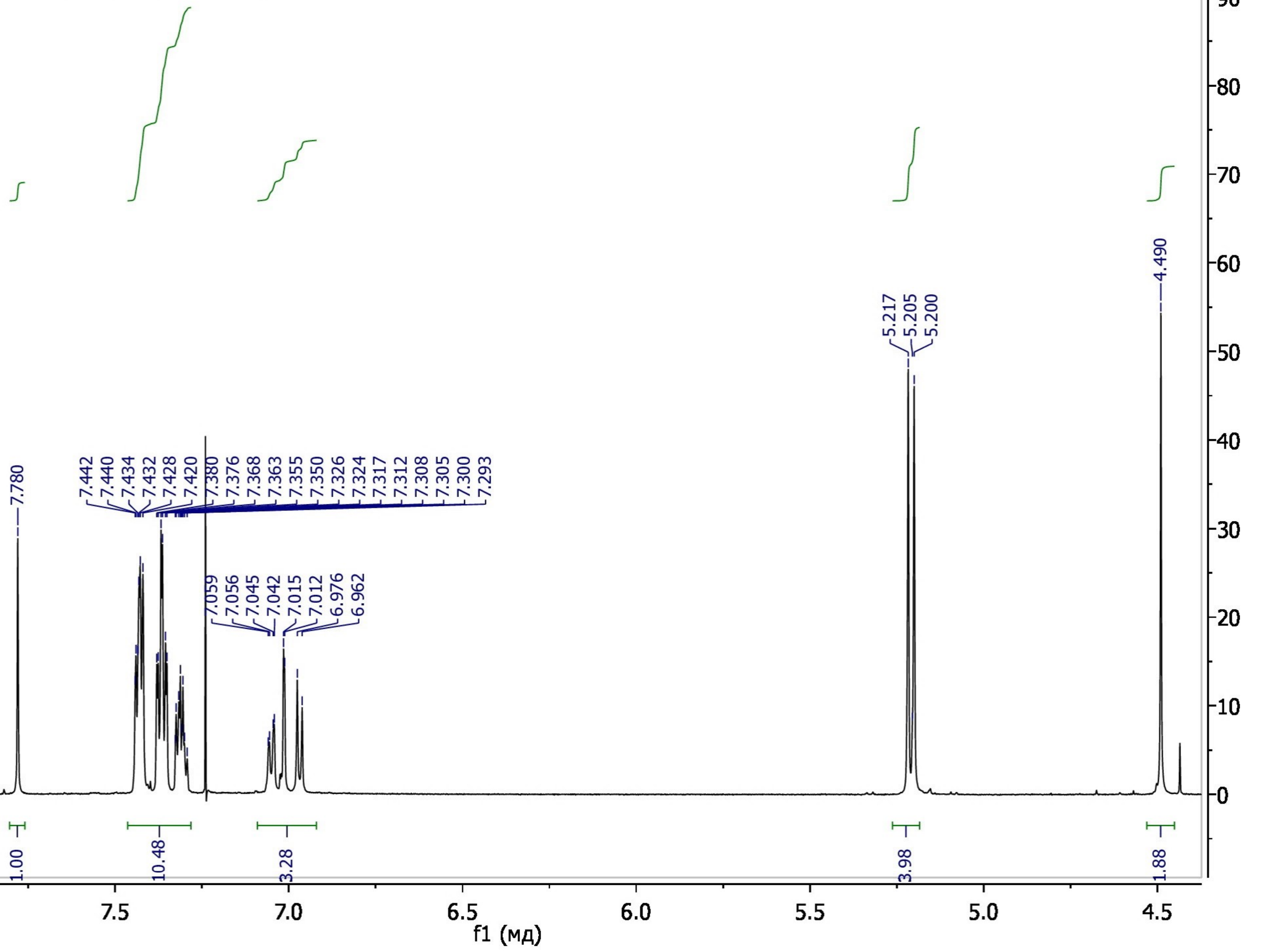

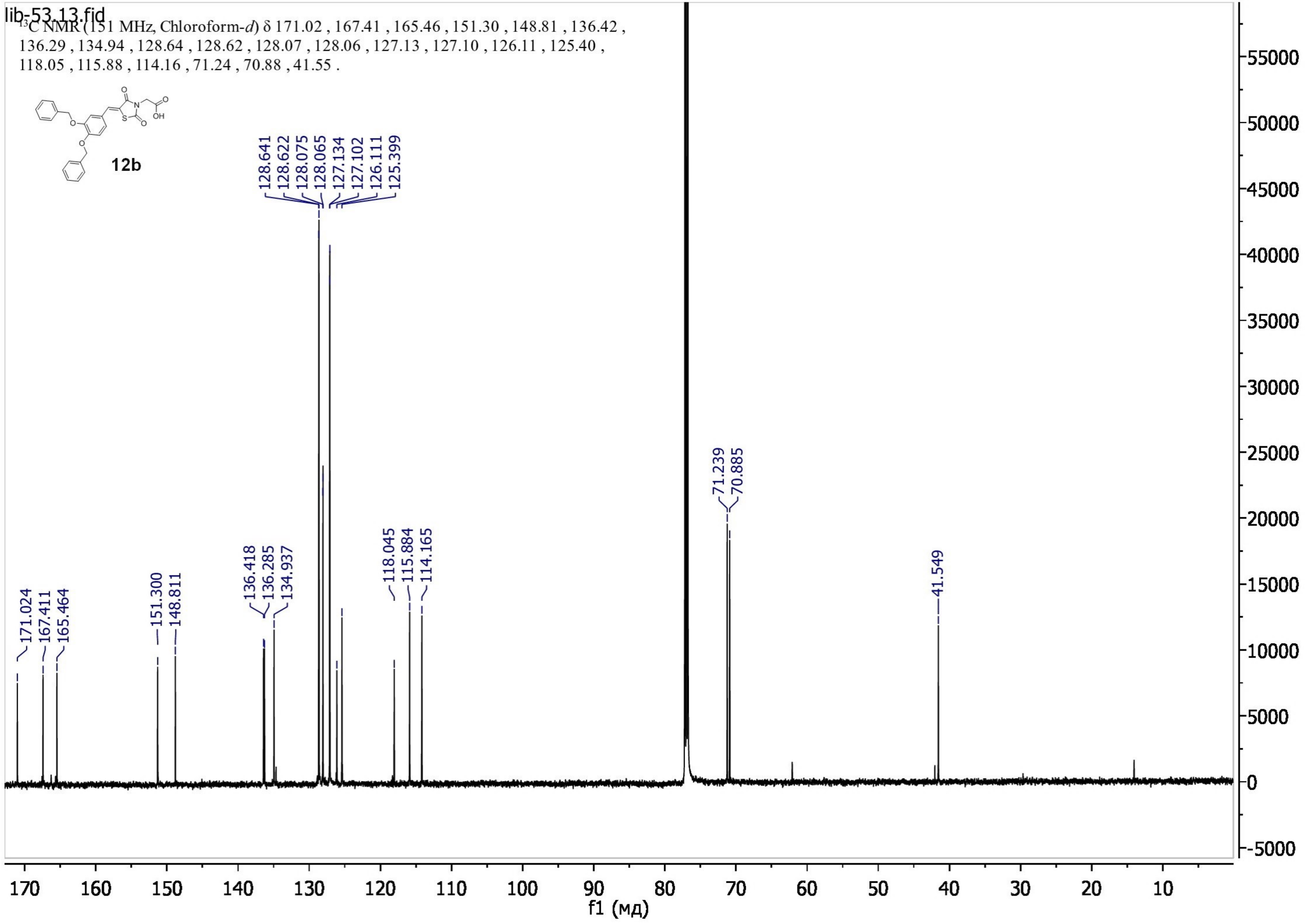
lib-53.13.fid
^{13}C NMR (151 MHz, Chloroform-*d*) δ 171.02 , 167.41 , 165.46 , 151.30 , 148.81 , 136.42 , 136.29 , 134.94 , 128.64 , 128.62 , 128.07 , 128.06 , 127.13 , 127.10 , 126.11 , 125.40 , 118.05 , 115.88 , 114.16 , 71.24 , 70.88 , 41.55 .
12b
171.024
167.411
165.464
151.300
148.811
136.418
136.285
134.937
128.641
128.622
128.075
128.065
127.134
127.102
126.111
125.399
118.045
115.884
114.165
71.239
70.885
41.549
f1 (мд)

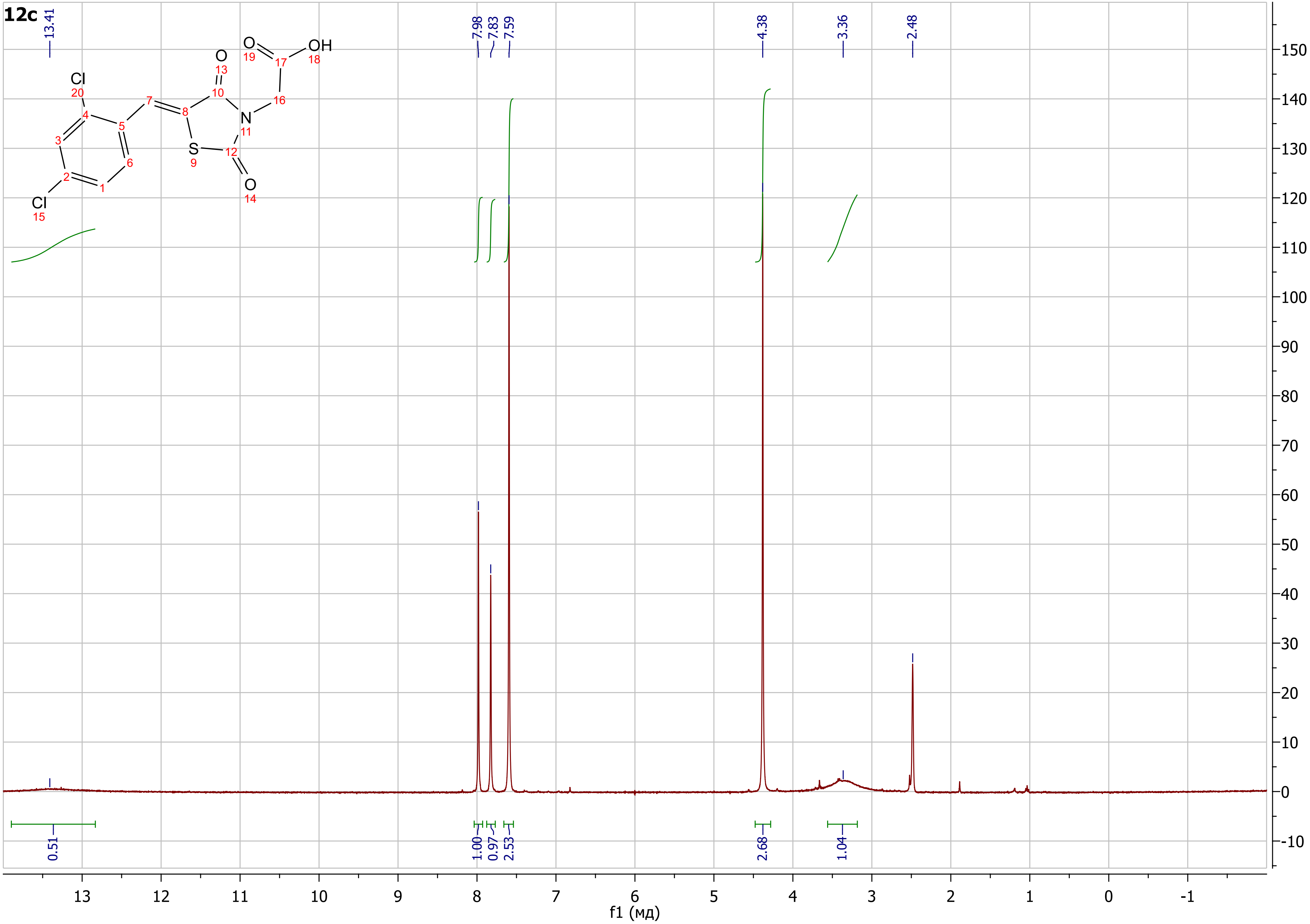
12c
13.41
7.98
7.83
7.59
4.38
3.36
2.48
O
OH
19
17
18
Cl
20
13
10
16
7
8
N
4
5
11
3
S
12
6
9
2
1
O
14
Cl
15
0.51
1.00
0.97
2.53
2.68
1.04
f1 (мд)

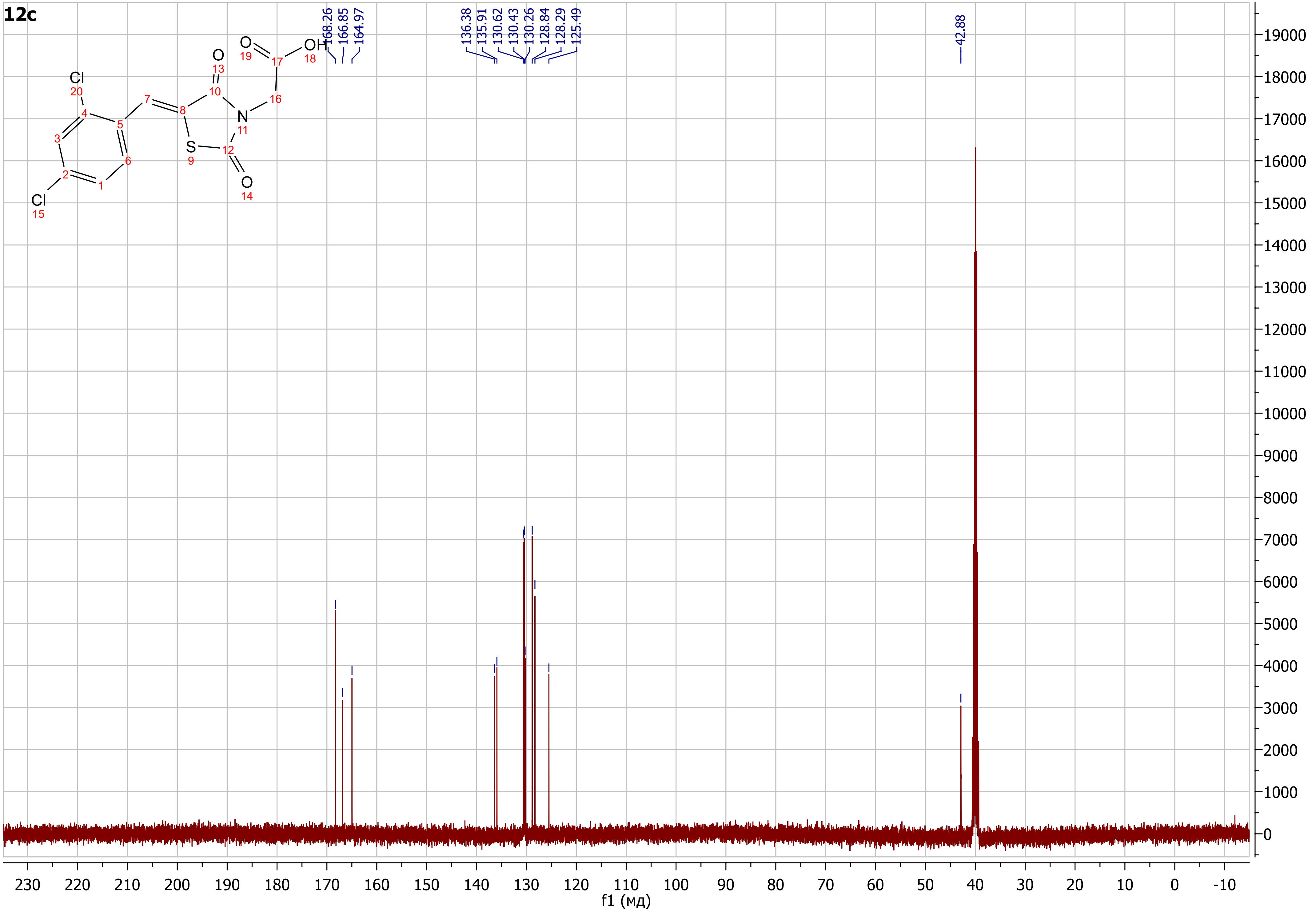
12c
Cl
20
4
3
5
6
2
1
Cl
15
7
8
S
9
10
O
13
N
11
12
O
14
16
17
O
19
OH
18
168.26
166.85
164.97
136.38
135.91
130.62
130.43
130.26
128.84
128.29
125.49
42.88
230
220
210
200
190
180
170
160
150
140
130
120
110
100
90
80
70
60
50
40
30
20
10
0
-10
f1 (мд)
19000
18000
17000
16000
15000
14000
13000
12000
11000
10000
9000
8000
7000
6000
5000
4000
3000
2000
1000
0

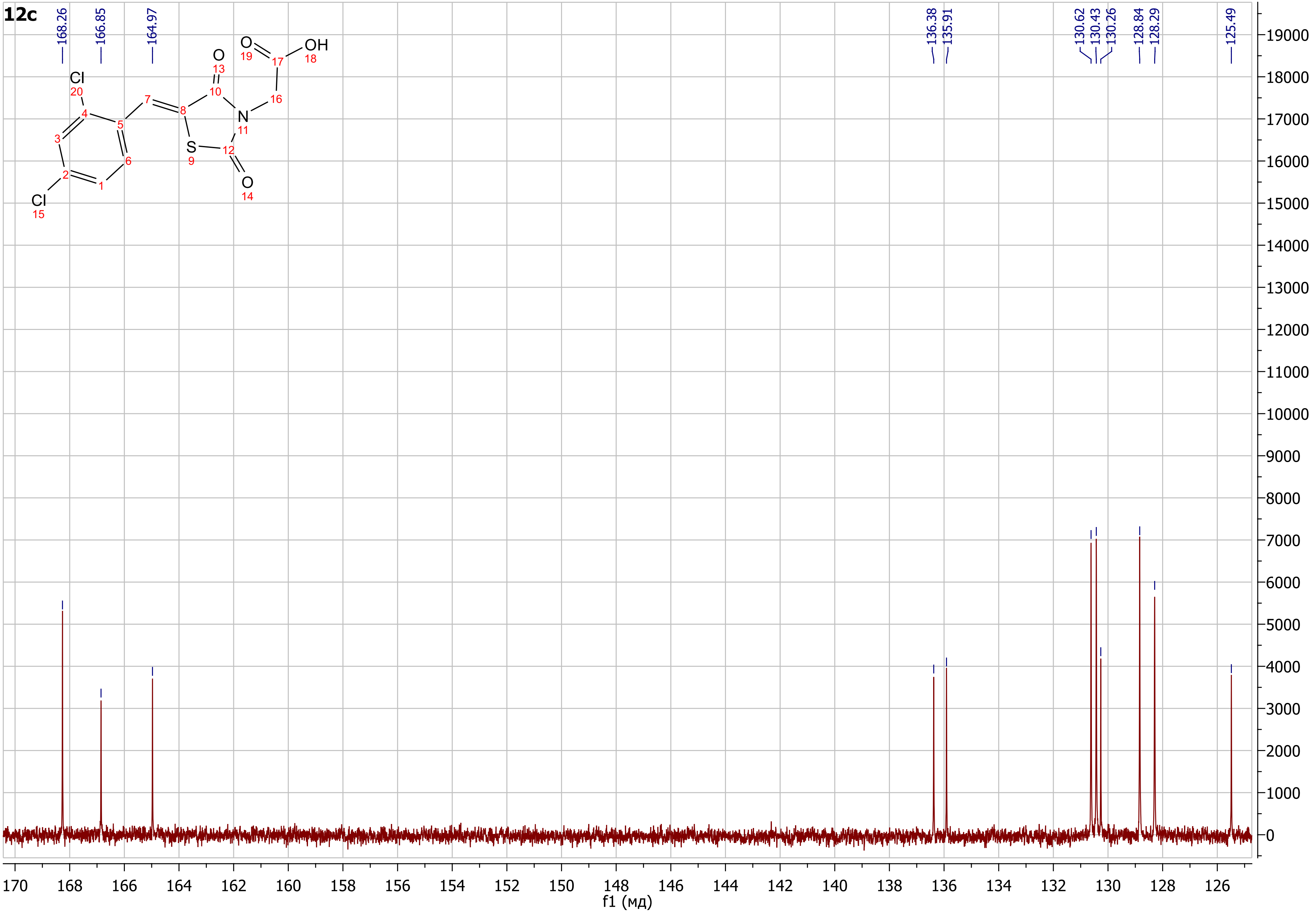
12c
168.26
166.85
164.97
136.38
135.91
130.62
130.43
130.26
128.84
128.29
125.49
Cl
20
4
3
2
1
Cl
15
5
6
7
8
S
9
10
O
13
N
11
12
O
14
16
17
O
19
OH
18
f1 (мд)

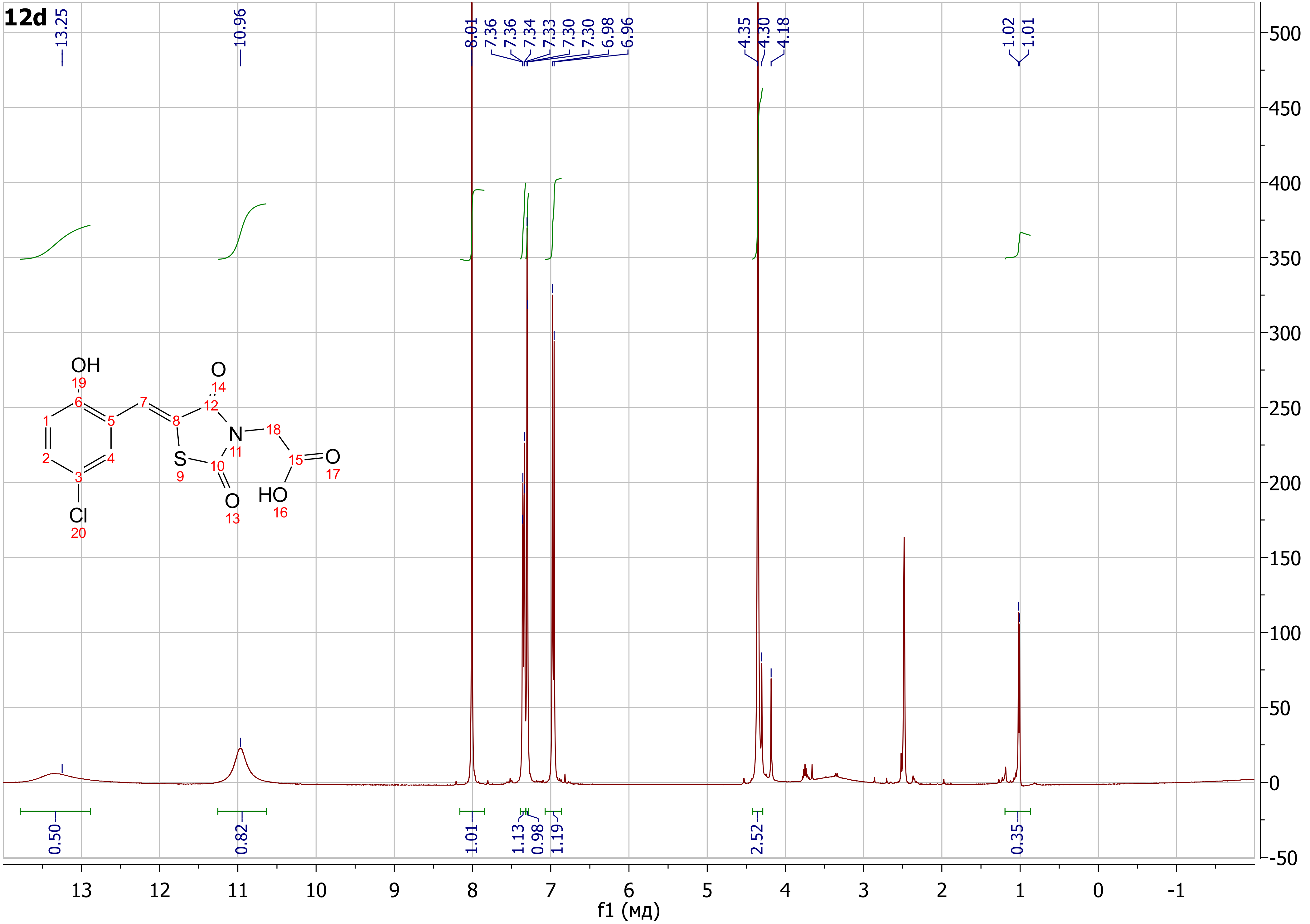

12d
13.25
10.96
8.01
7.36
7.36
7.34
7.33
7.30
7.30
6.98
6.96
4.35
4.30
4.18
1.02
1.01
OH
O
N
S
O
HO
O
Cl
0.50
0.82
1.01
1.13
0.98
1.19
2.52
0.35
f1 (мд)

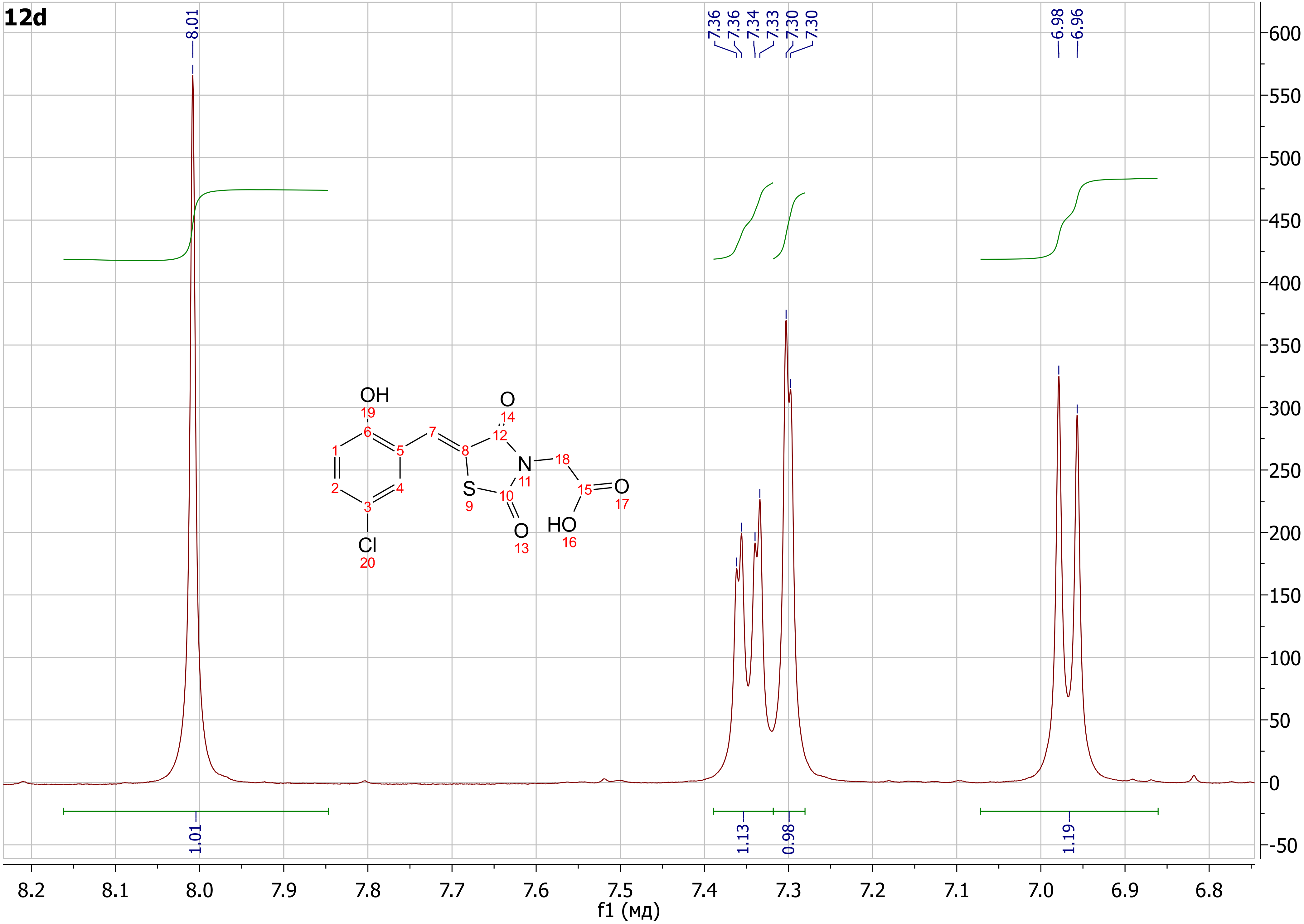
12d
8.01
7.36
7.36
7.34
7.33
7.30
7.30
6.98
6.96
1.01
1.13
0.98
1.19
OH
Cl
S
N
O
HO
8.2
8.1
8.0
7.9
7.8
7.7
7.6
7.5
7.4
7.3
7.2
7.1
7.0
6.9
6.8
f1 (мд)
600
550
500
450
400
350
300
250
200
150
100
50
0
-50

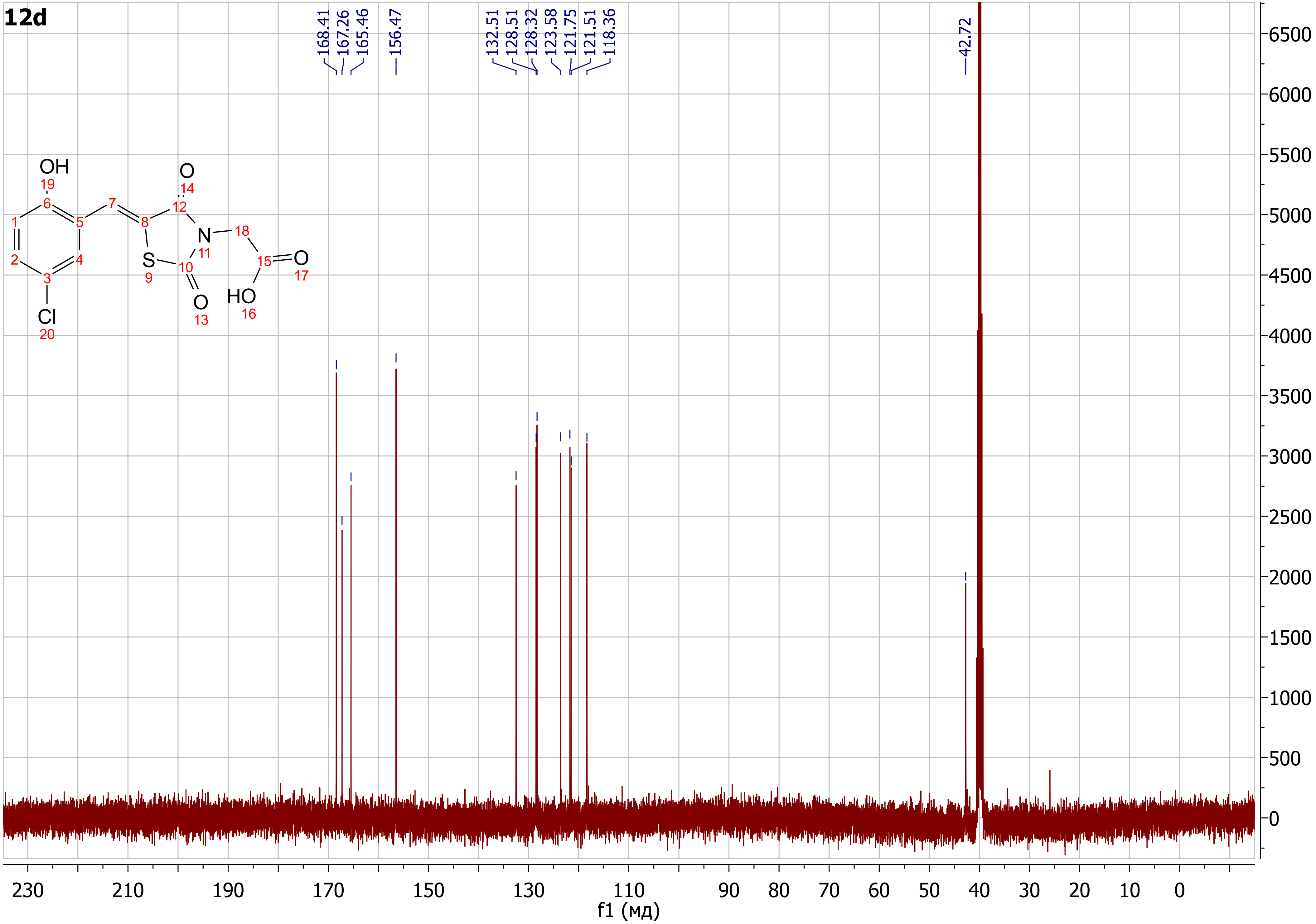
12d
168.41
167.26
165.46
156.47
132.51
128.51
128.32
123.58
121.75
121.51
118.36
42.72
OH
19
6
1
5
7
8
12
O
14
N
11
18
15
O
17
HO
16
S
9
10
O
13
2
3
4
Cl
20
f1 (мд)

12e

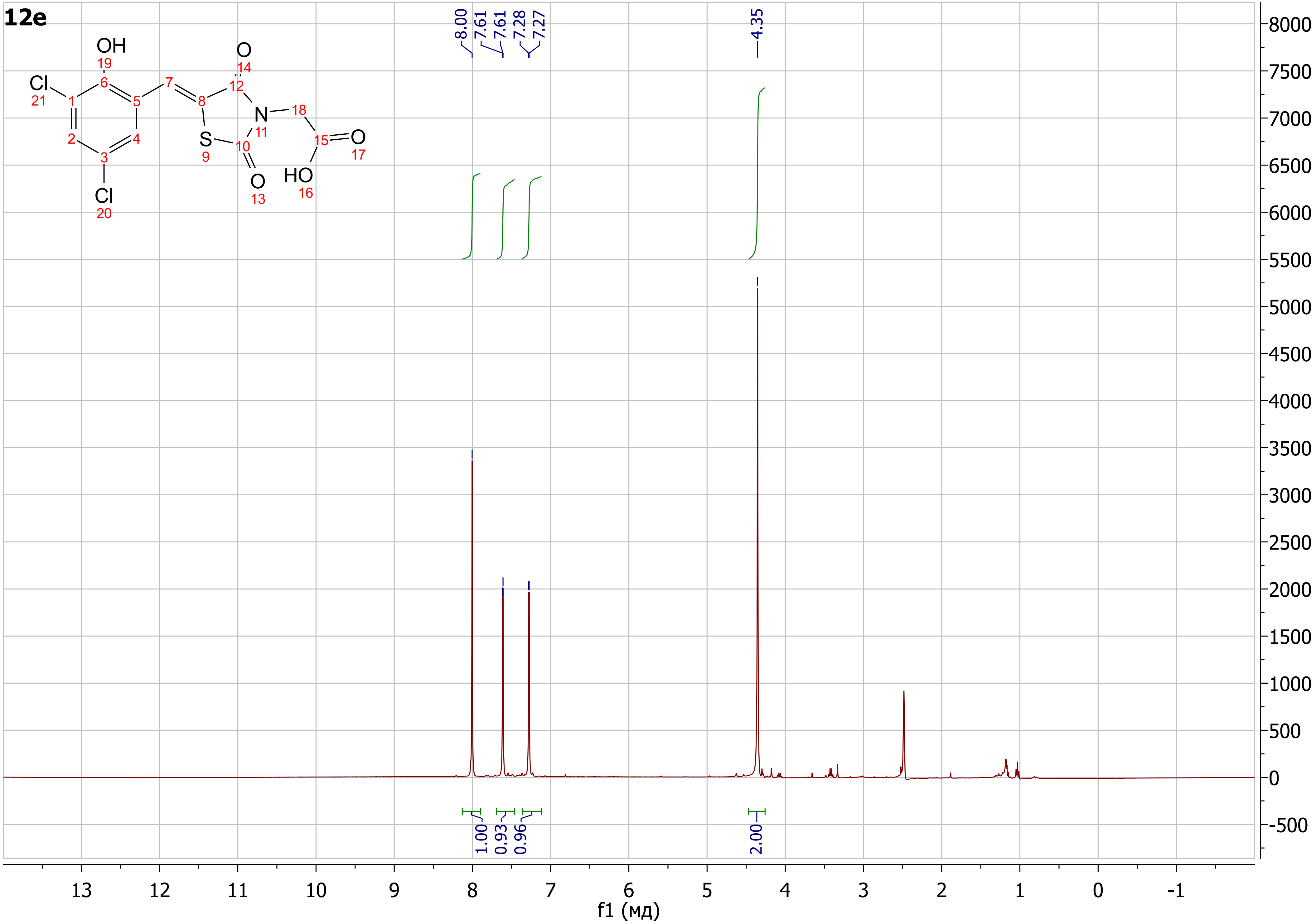

OH
19
Cl
21
O
14
N
11
18
S
9
O
17
HO
16
O
13
Cl
20
8.00
7.61
7.61
7.28
7.27
4.35
1.00
0.93
0.96
2.00
f1 (мд)

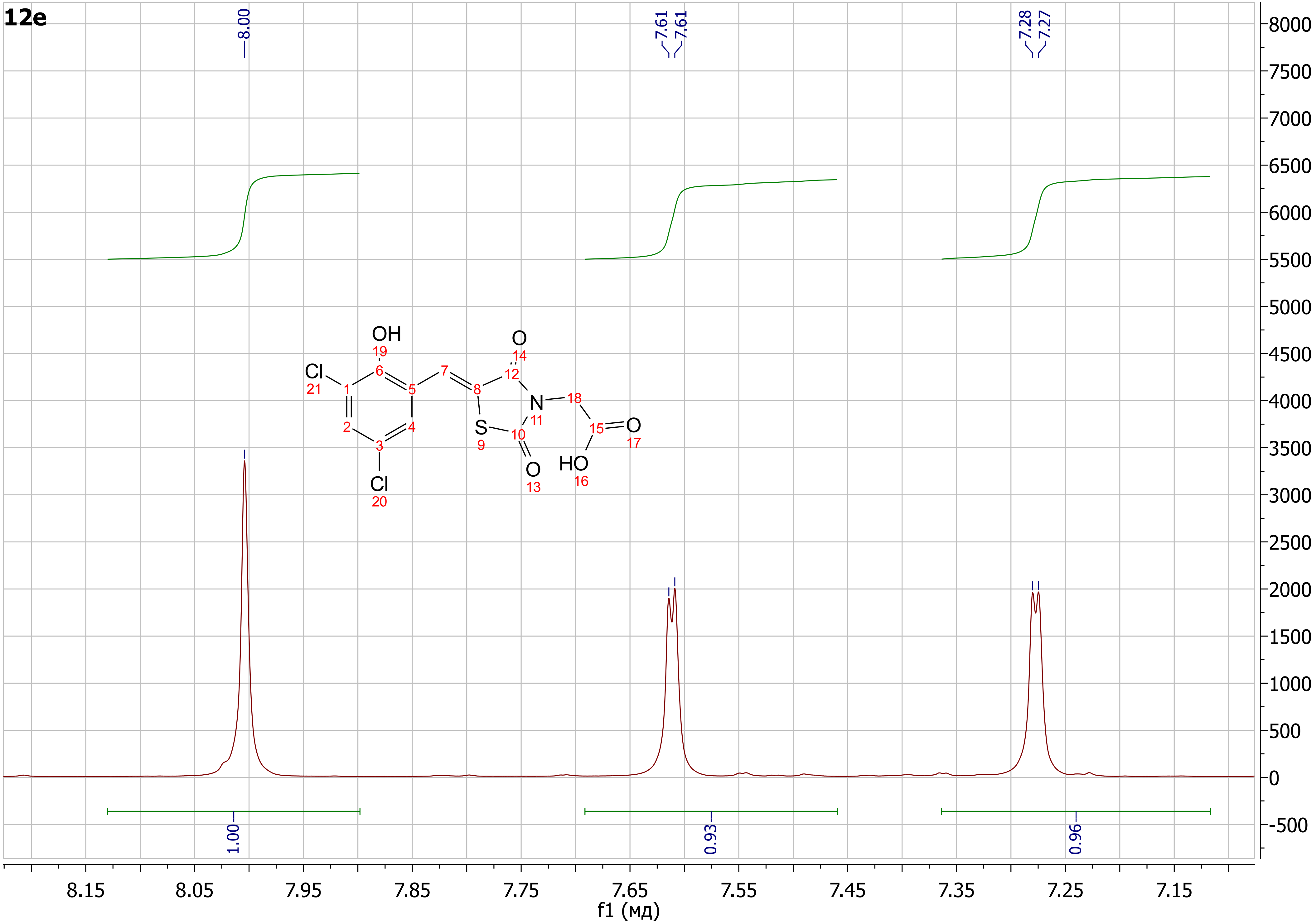

12e
8.00
7.61
7.61
7.28
7.27
OH
19
Cl
21
6
1
5
7
8
12
14
O
N
11
18
15
O
17
2
4
3
S
9
10
O
13
HO
16
Cl
20
1.00
0.93
0.96
8.15
8.05
7.95
7.85
7.75
7.65
7.55
7.45
7.35
7.25
7.15
f1 (мд)
8000
7500
7000
6500
6000
5500
5000
4500
4000
3500
3000
2500
2000
1500
1000
500
0
-500

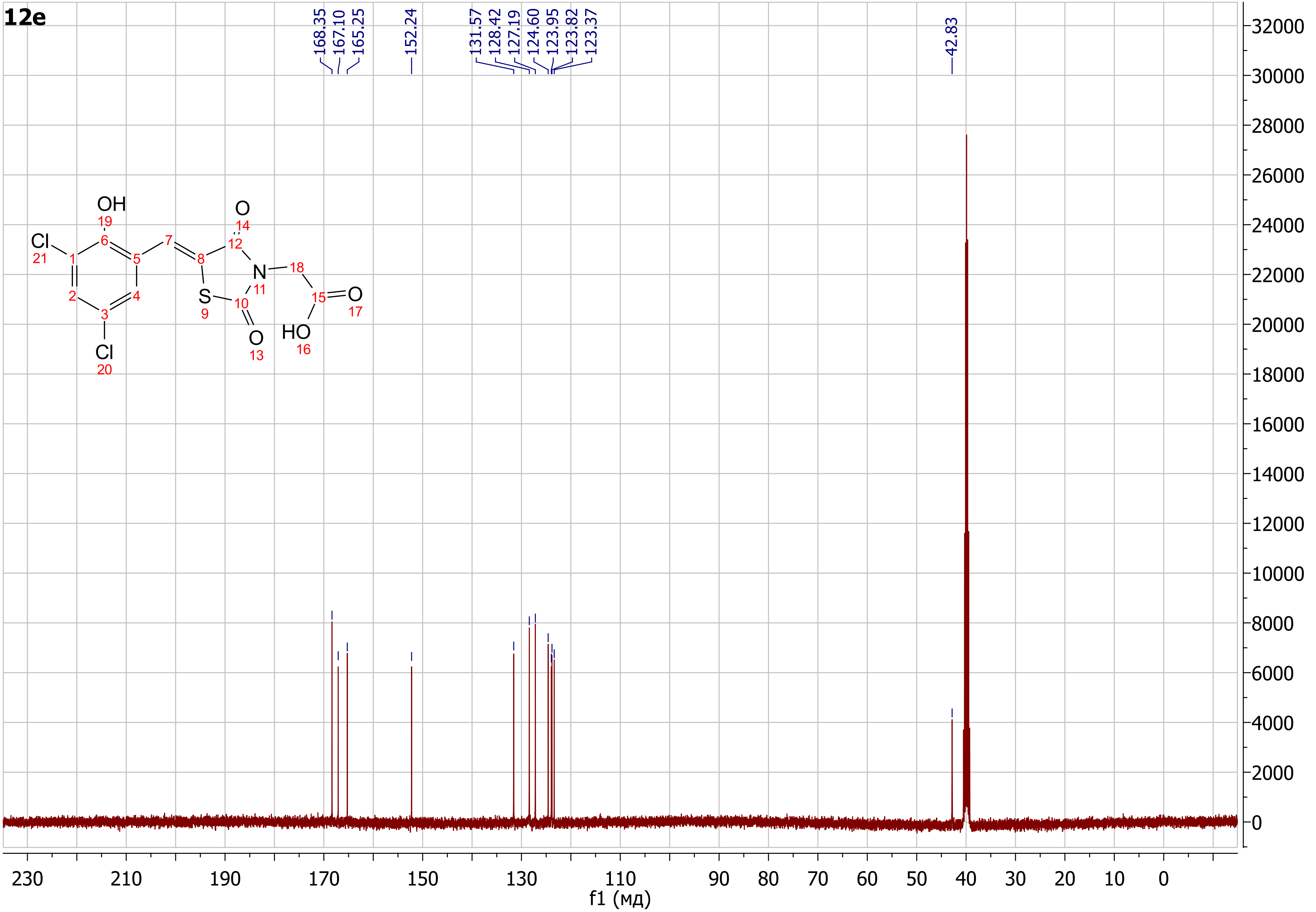

12e
168.35
167.10
165.25
152.24
131.57
128.42
127.19
124.60
123.95
123.82
123.37
42.83
OH
19
Cl
21
6
7
5
8
1
2
4
3
Cl
20
O
14
12
N
11
18
S
9
10
O
13
15
O
17
HO
16
230
210
190
170
150
130
110
90
80
70
60
50
40
30
20
10
0
f1 (мд)
32000
30000
28000
26000
24000
22000
20000
18000
16000
14000
12000
10000
8000
6000
4000
2000
0

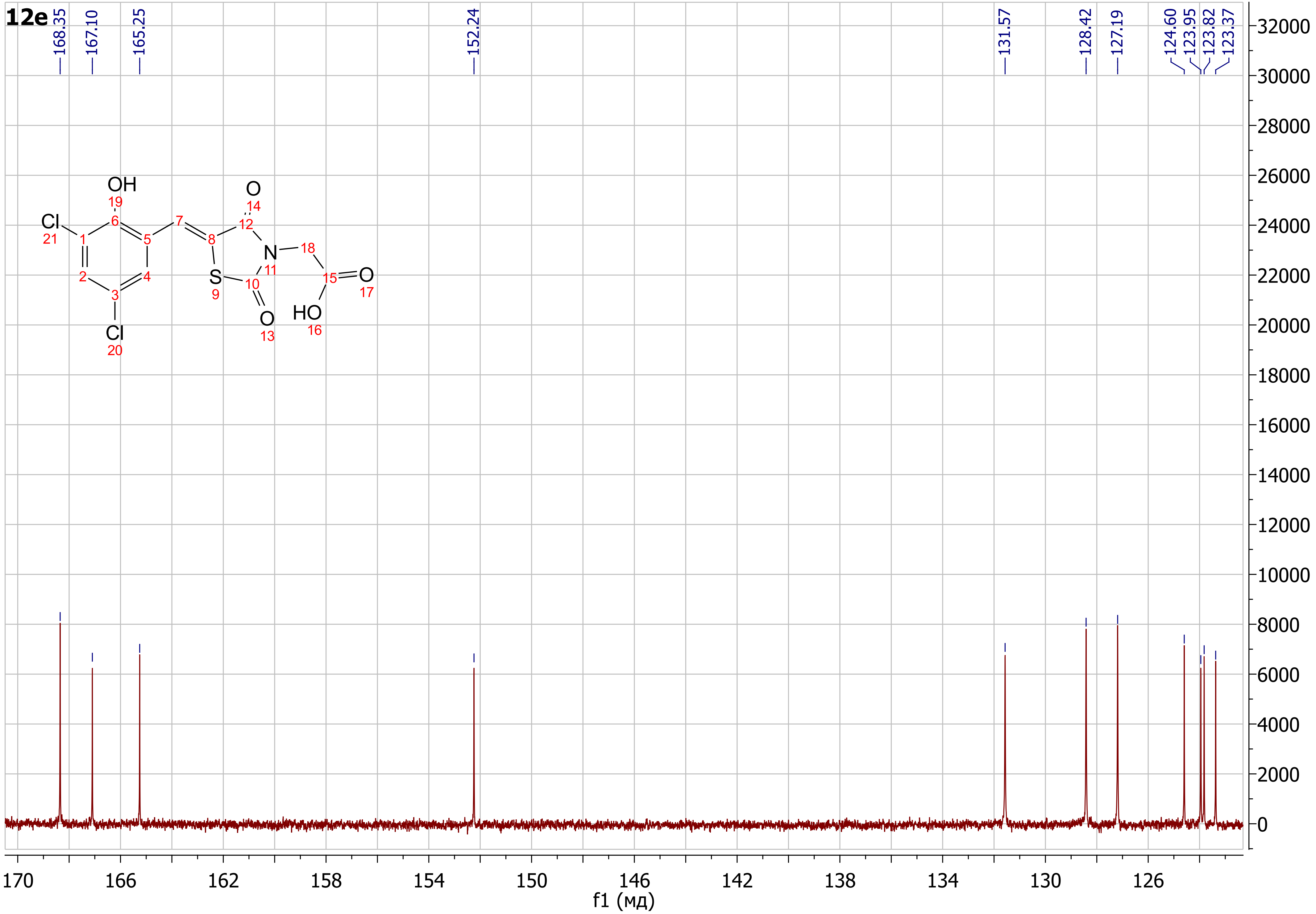

12e
168.35
167.10
165.25
152.24
131.57
128.42
127.19
124.60
123.95
123.82
123.37
OH
19
Cl
21
1
2
3
4
5
6
7
8
S
9
10
N
11
12
O
14
O
13
18
15
O
17
HO
16
Cl
20
f1 (мд)

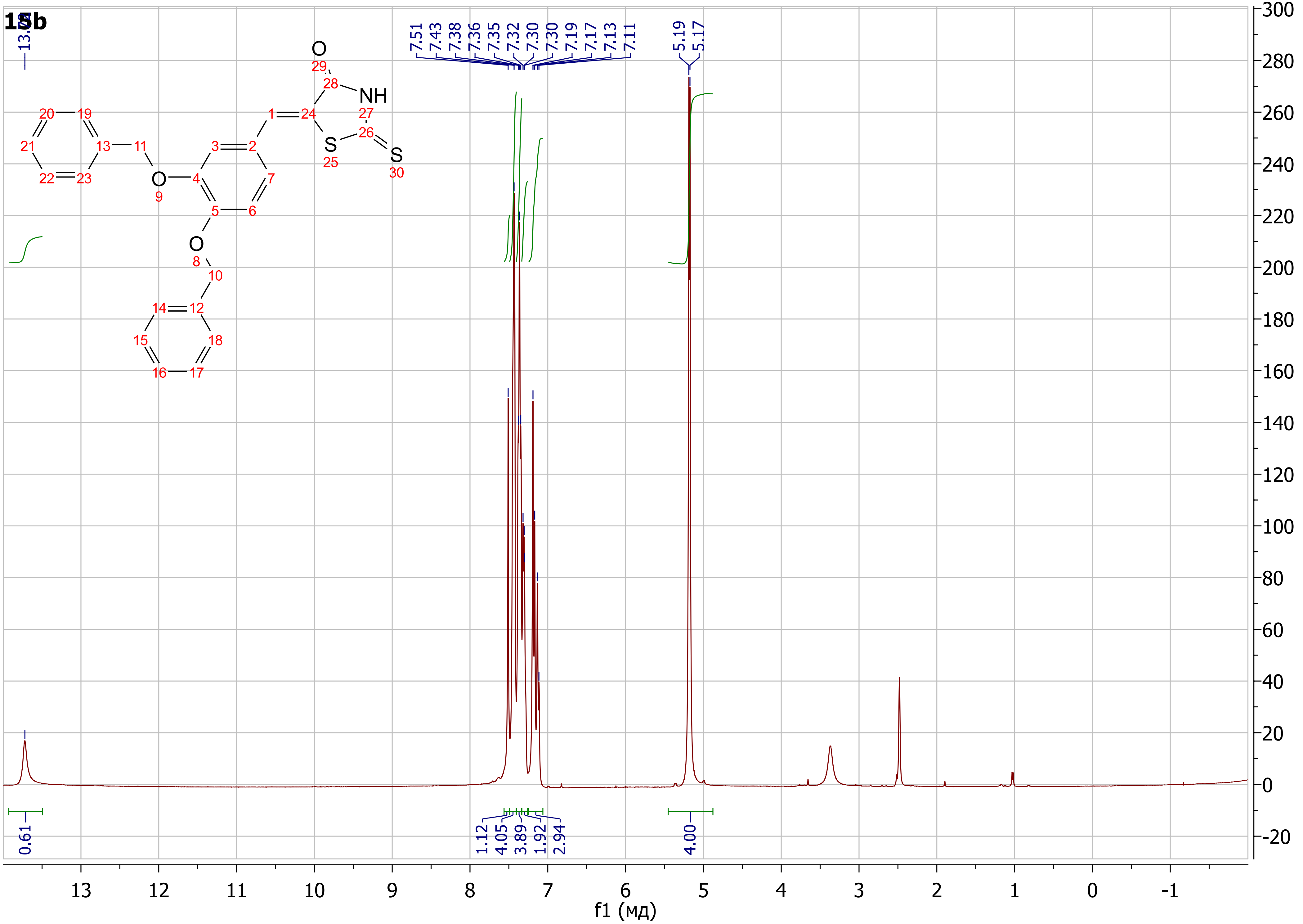

15b
7.51
7.43
7.38
7.36
7.35
7.32
7.30
7.30
7.19
7.17
7.13
7.11
5.19
5.17
O
NH
S
S
O
O
0.61
1.12
4.05
3.89
1.92
2.94
4.00
f1 (мд)

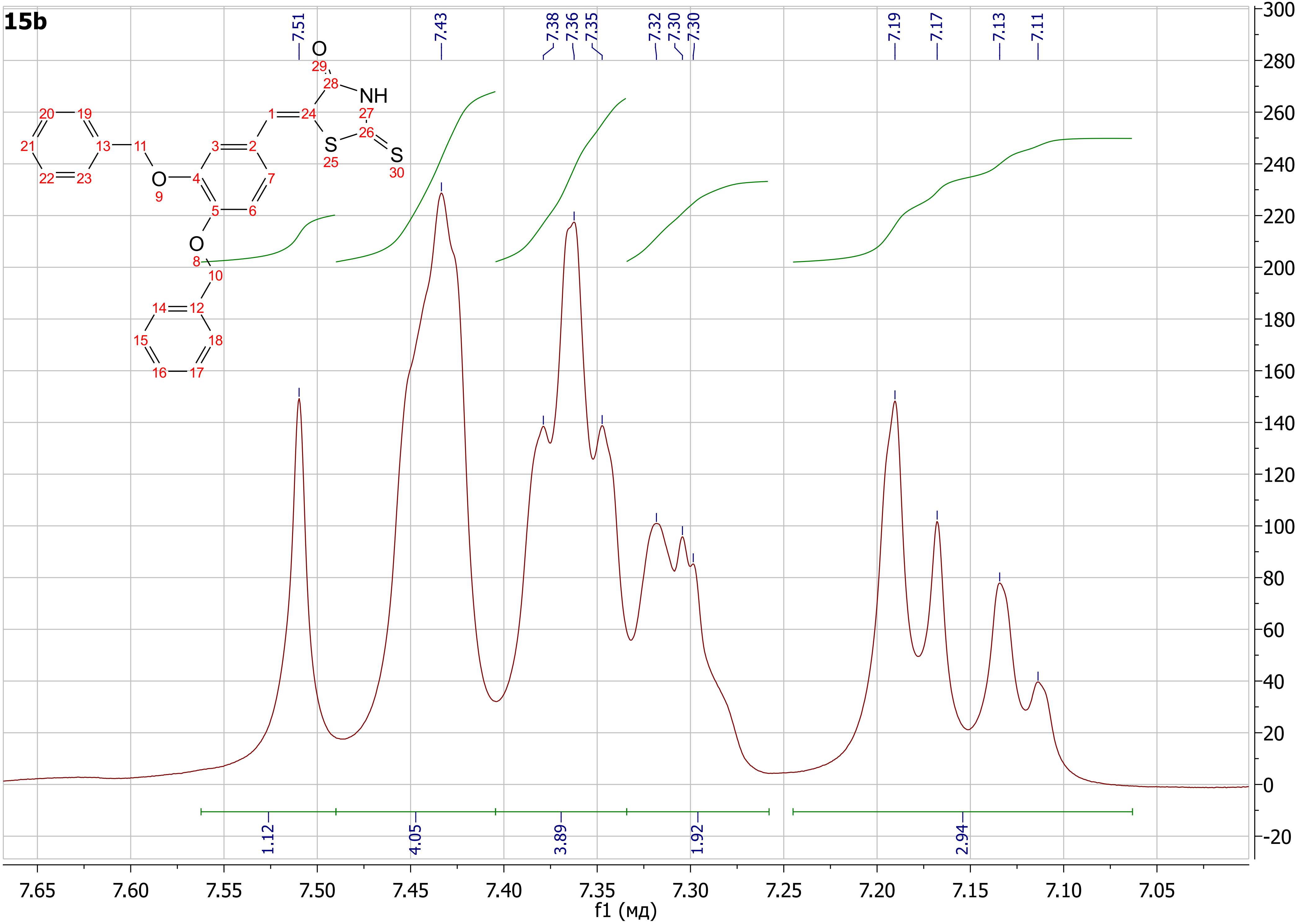

15b
7.51
7.43
7.38
7.36
7.35
7.32
7.30
7.30
7.19
7.17
7.13
7.11
1.12
4.05
3.89
1.92
2.94
O
NH
S
S
O
O
f1 (мд)

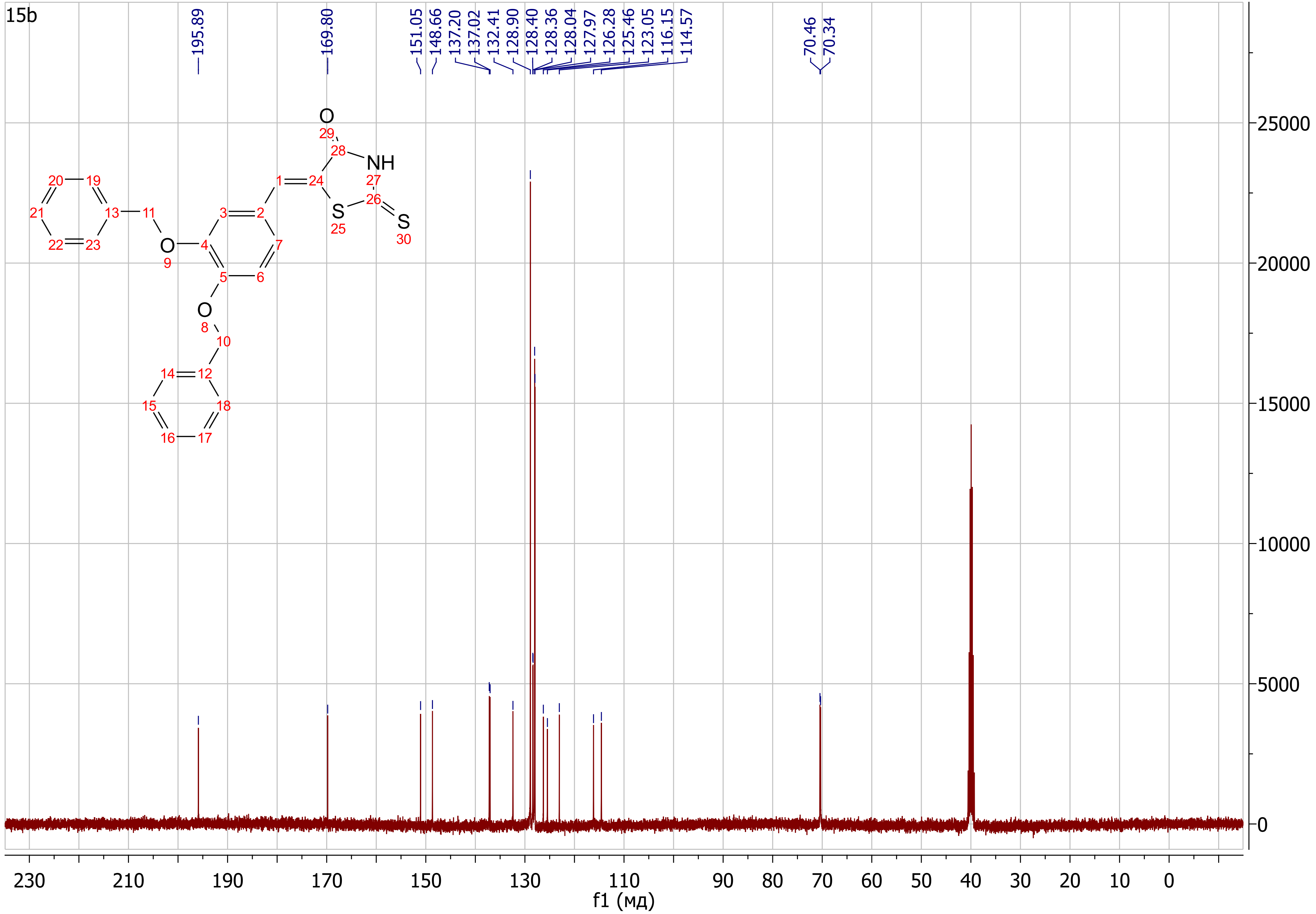

15b
195.89
169.80
151.05
148.66
137.20
137.02
132.41
128.90
128.40
128.36
128.04
127.97
126.28
125.46
123.05
116.15
114.57
70.46
70.34
O
NH
S
S
O
O
25000
20000
15000
10000
5000
0
230
210
190
170
150
130
110
90
80
70
60
50
40
30
20
10
0
f1 (мд)

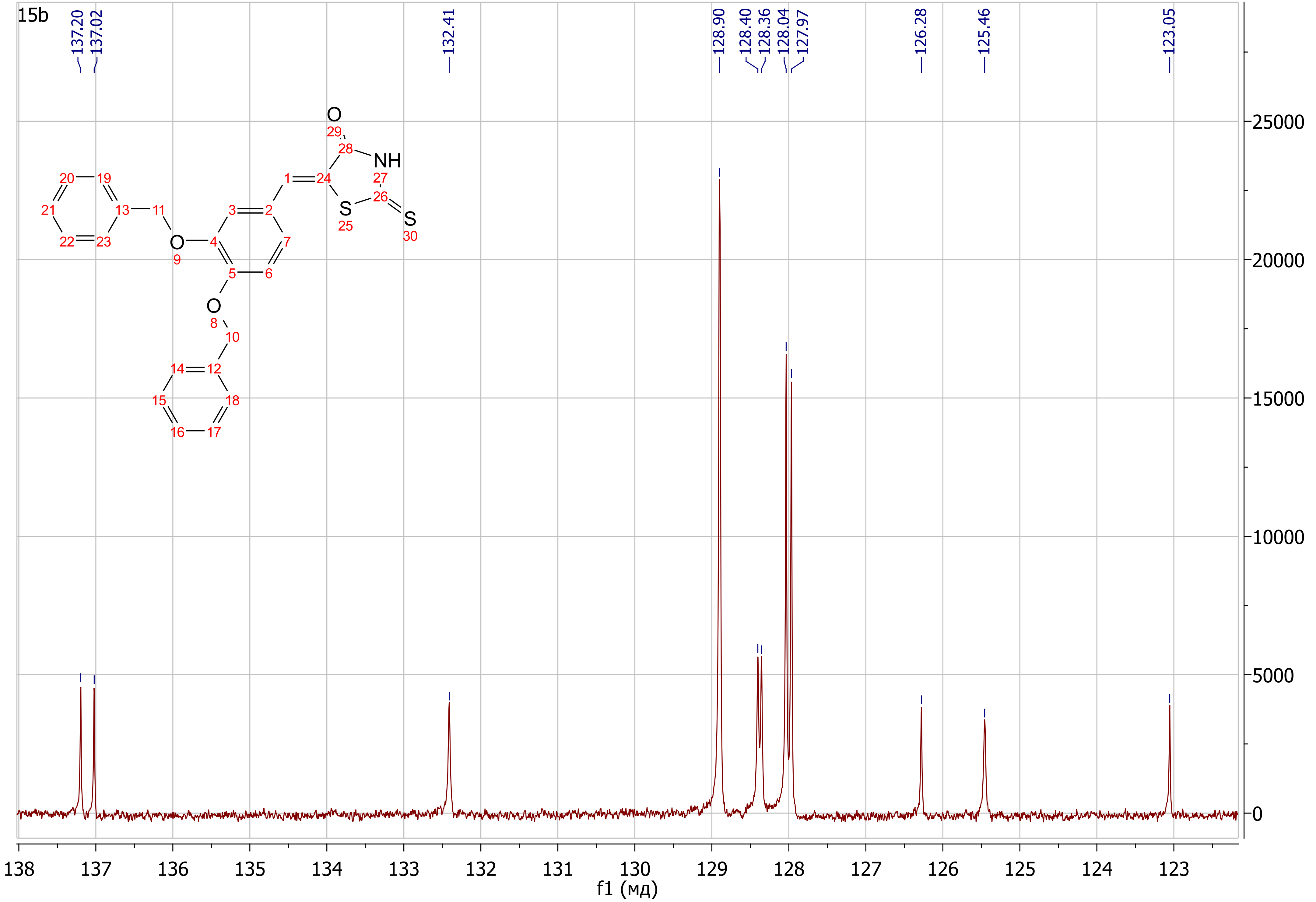

15b
137.20
137.02
132.41
128.90
128.40
128.36
128.04
127.97
126.28
125.46
123.05
O
NH
S
S
O
O
25000
20000
15000
10000
5000
0
138
137
136
135
134
133
132
131
130
129
128
127
126
125
124
123
f1 (мд)

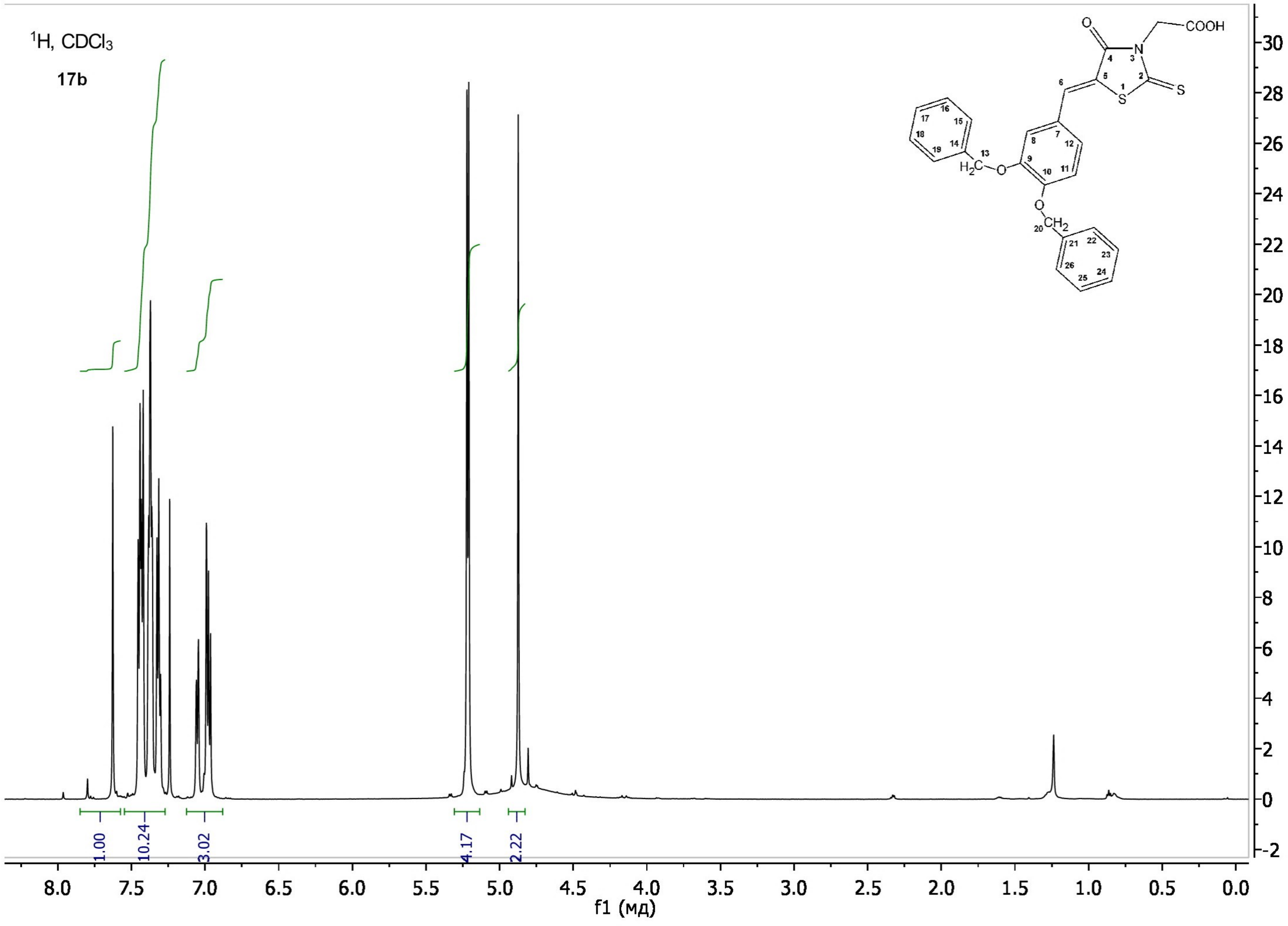

¹H, CDCl3
17b
COOH
1.00
10.24
3.02
4.17
2.22
f1 (мд)

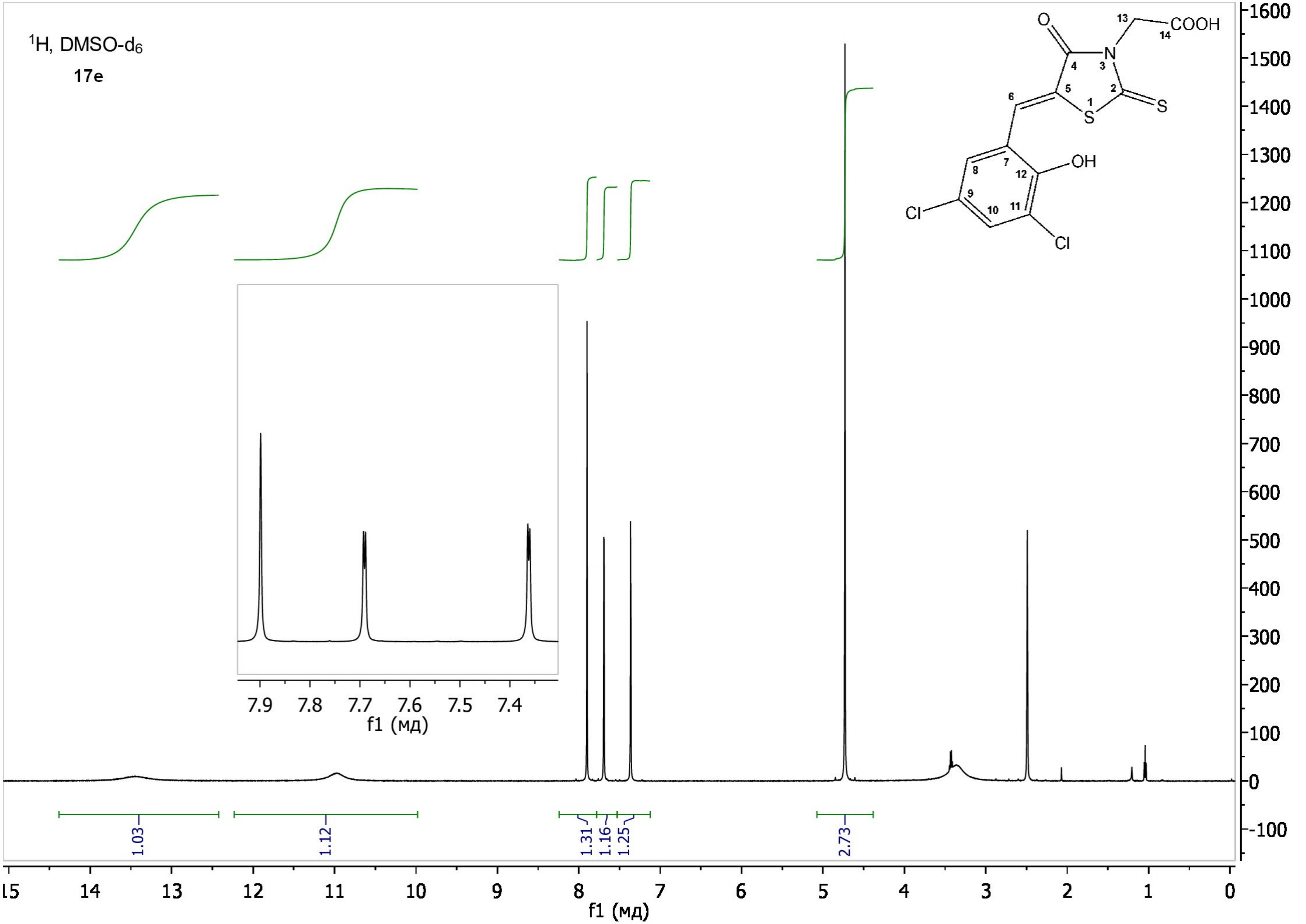

^{1}H, DMSO-d$_6$
17e
O
13
COOH
14
4
3
N
5
2
6
1
S
S
8
7
12
OH
9
Cl
10
11
Cl
1.03
1.12
1.31
1.16
1.25
2.73
7.9
7.8
7.7
7.6
7.5
7.4
f1 (мд)
15
14
13
12
11
10
9
8
7
6
5
4
3
2
1
0
f1 (мд)
1600
1500
1400
1300
1200
1100
1000
900
800
700
600
500
400
300
200
100
0
-100

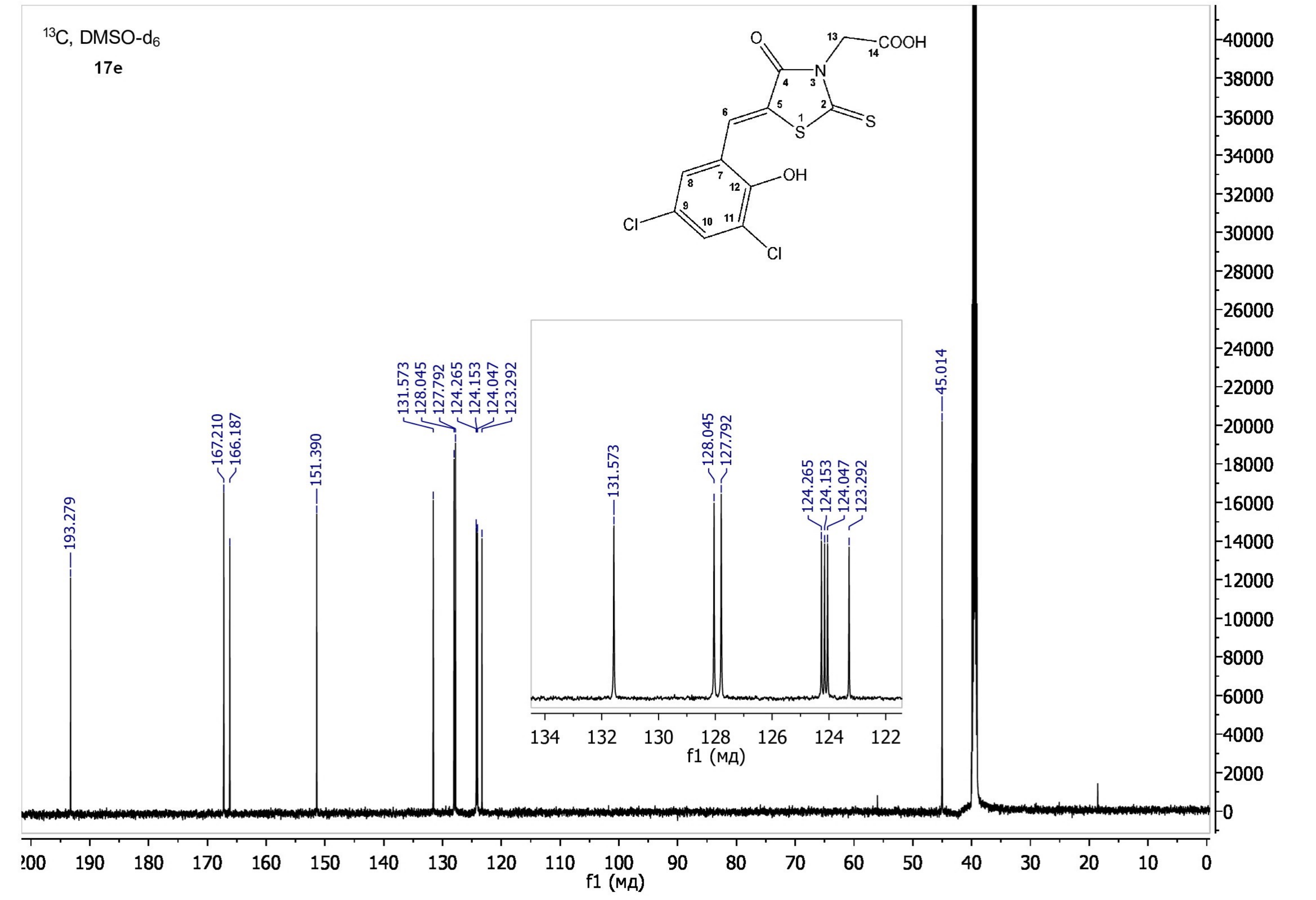

13C, DMSO-d6
17e
O
N
COOH
S
S
OH
Cl
Cl
1 2 3 4 5 6 7 8 9 10 11 12 13 14
193.279
167.210
166.187
151.390
131.573
128.045
127.792
124.265
124.153
124.047
123.292
45.014
f1 (мд)
