# Supplementary material for: Antifungal Thiazolidines: Synthesis and Biological Evaluation of Mycosidine Congeners
Source: Pharmaceuticals (Basel). 2022 May 1;15(5):563. doi: 10.3390/ph15050563 (PMC9145892; doi:10.3390/ph15050563)
Supplement: Supplementary file 1 [file pharmaceuticals-15-00563-s001.zip › Supplement_2d_spectra.pdf]

DQF-COSY

11b

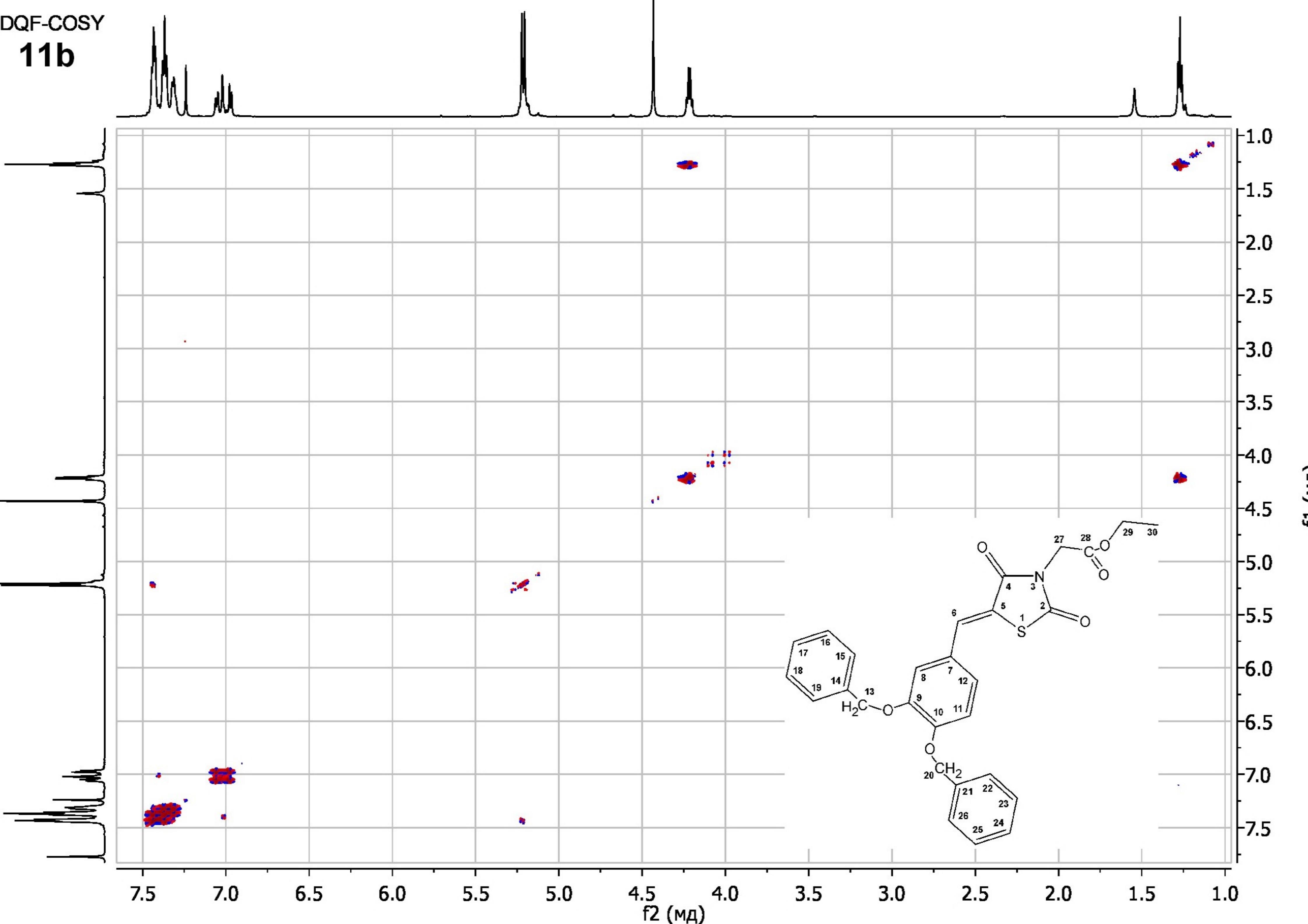

7.5
7.0
6.5
6.0
5.5
5.0
4.5
4.0
3.5
3.0
2.5
2.0
1.5
1.0
f2 (мд)
O
N
O
O
O
S
H2C
O
O
CH2
1
2
3
4
5
6
7
8
9
10
11
12
13
14
15
16
17
18
19
20
21
22
23
24
25
26
27
28
29
30

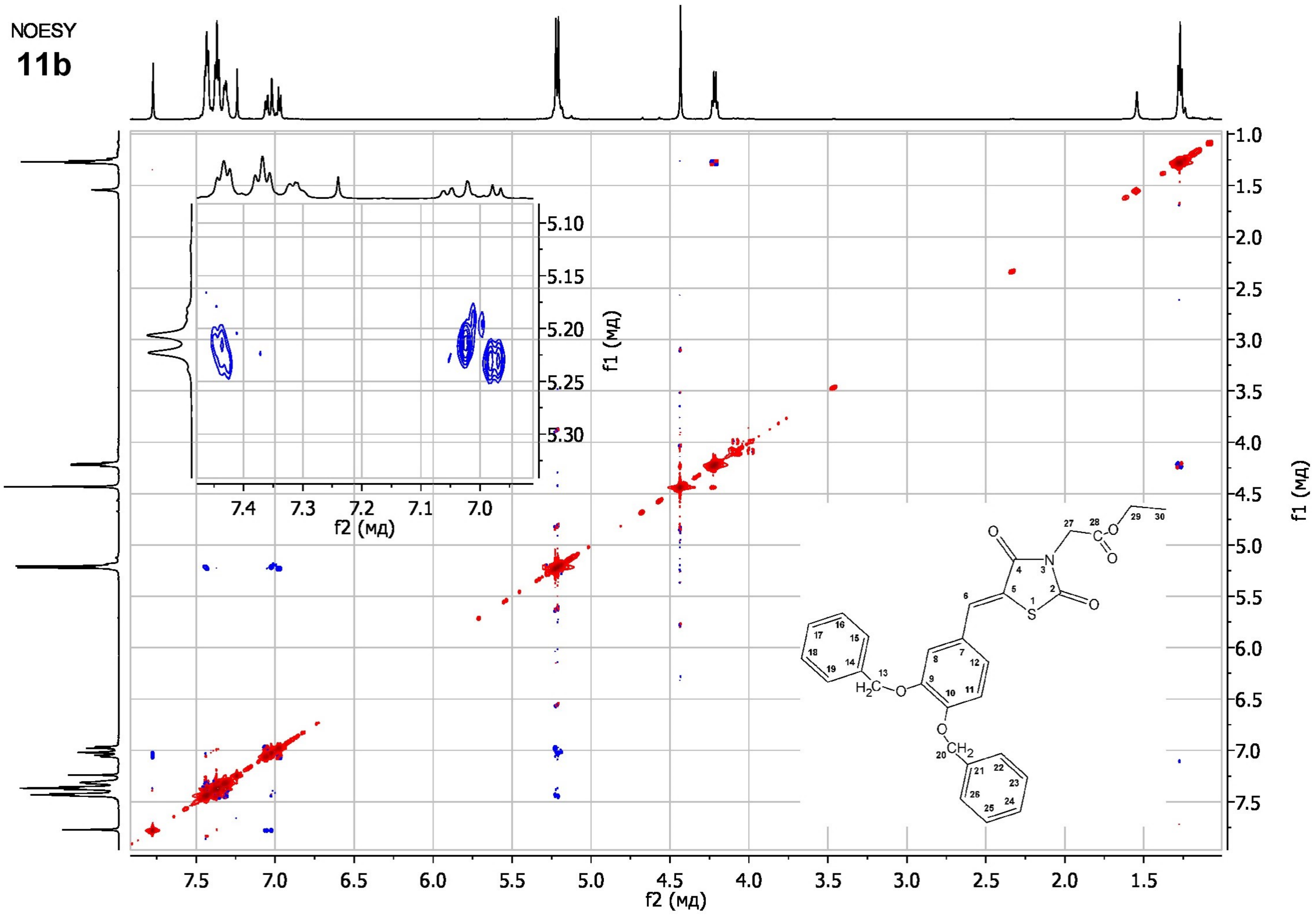
NOESY
11b
f2 (мд)
f1 (мд)

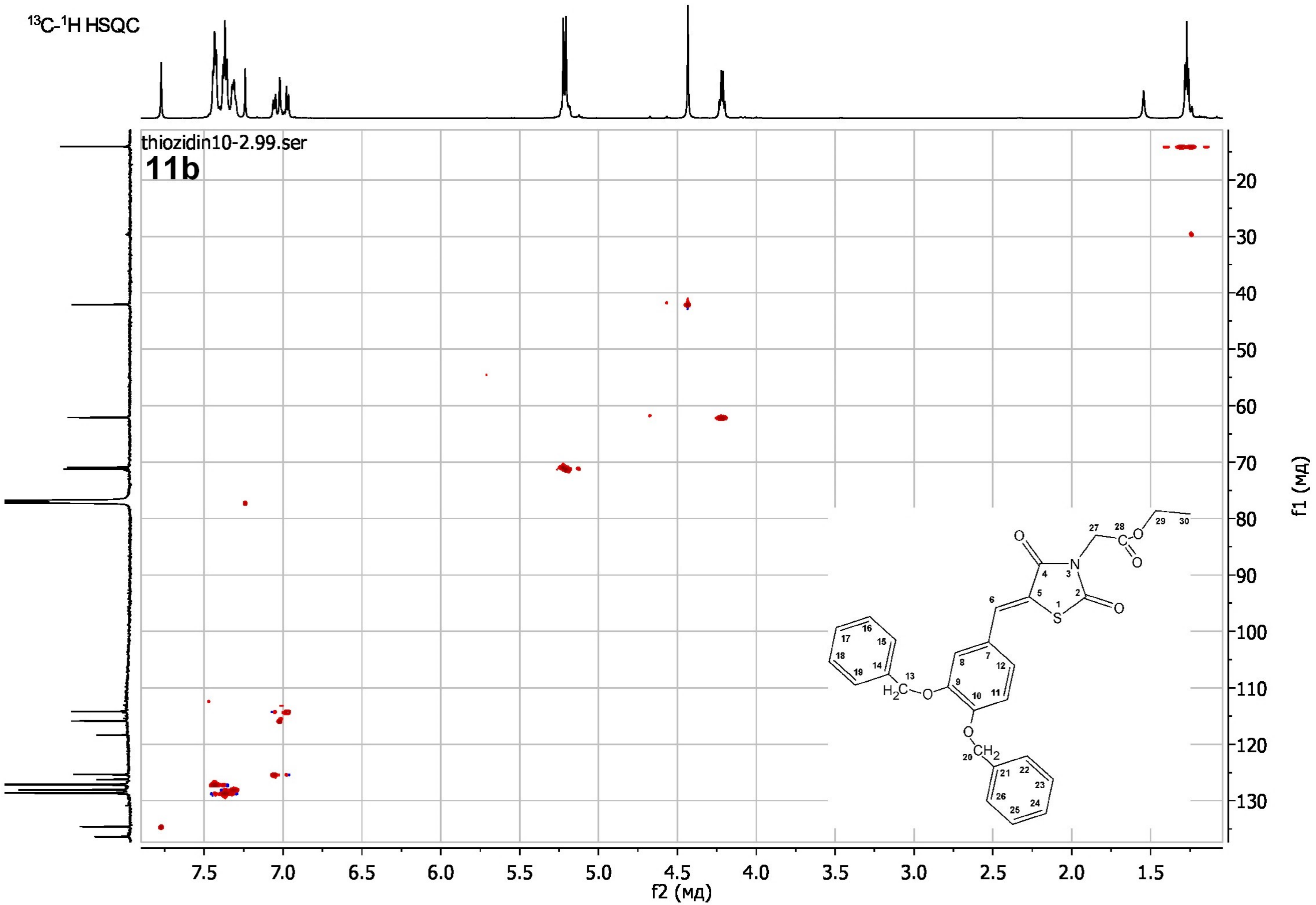

^{13}C-^{1}H HSQC
thiozidin10-2.99.ser
11b
f2 (мд)
f1 (мд)
7.5
7.0
6.5
6.0
5.5
5.0
4.5
4.0
3.5
3.0
2.5
2.0
1.5
20
30
40
50
60
70
80
90
100
110
120
130

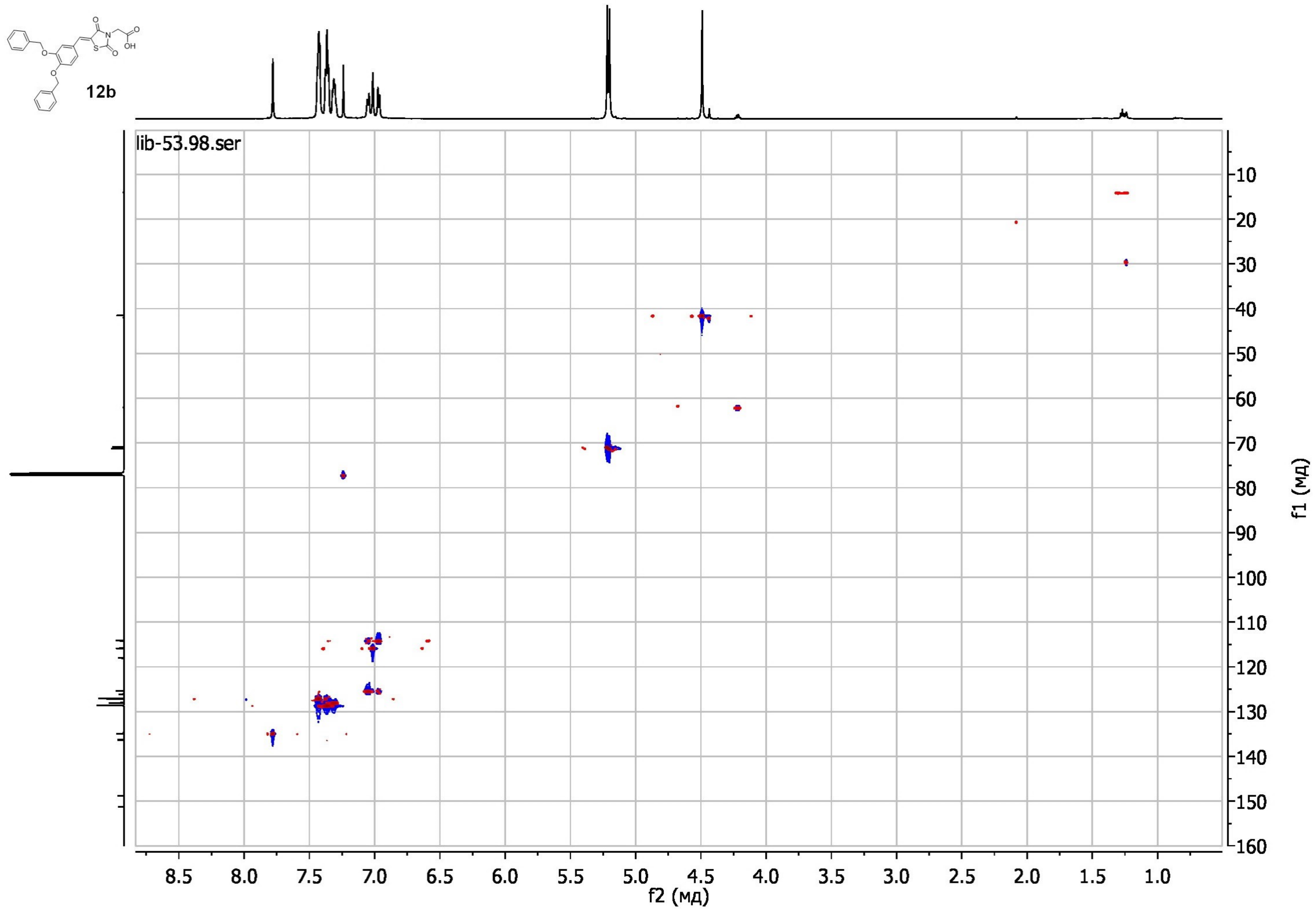
12b
lib-53.98.ser
f1 (мд)
f2 (мд)
10
20
30
40
50
60
70
80
90
100
110
120
130
140
150
160
8.5
8.0
7.5
7.0
6.5
6.0
5.5
5.0
4.5
4.0
3.5
3.0
2.5
2.0
1.5
1.0

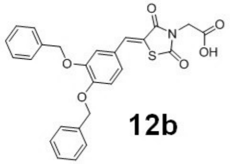

12b

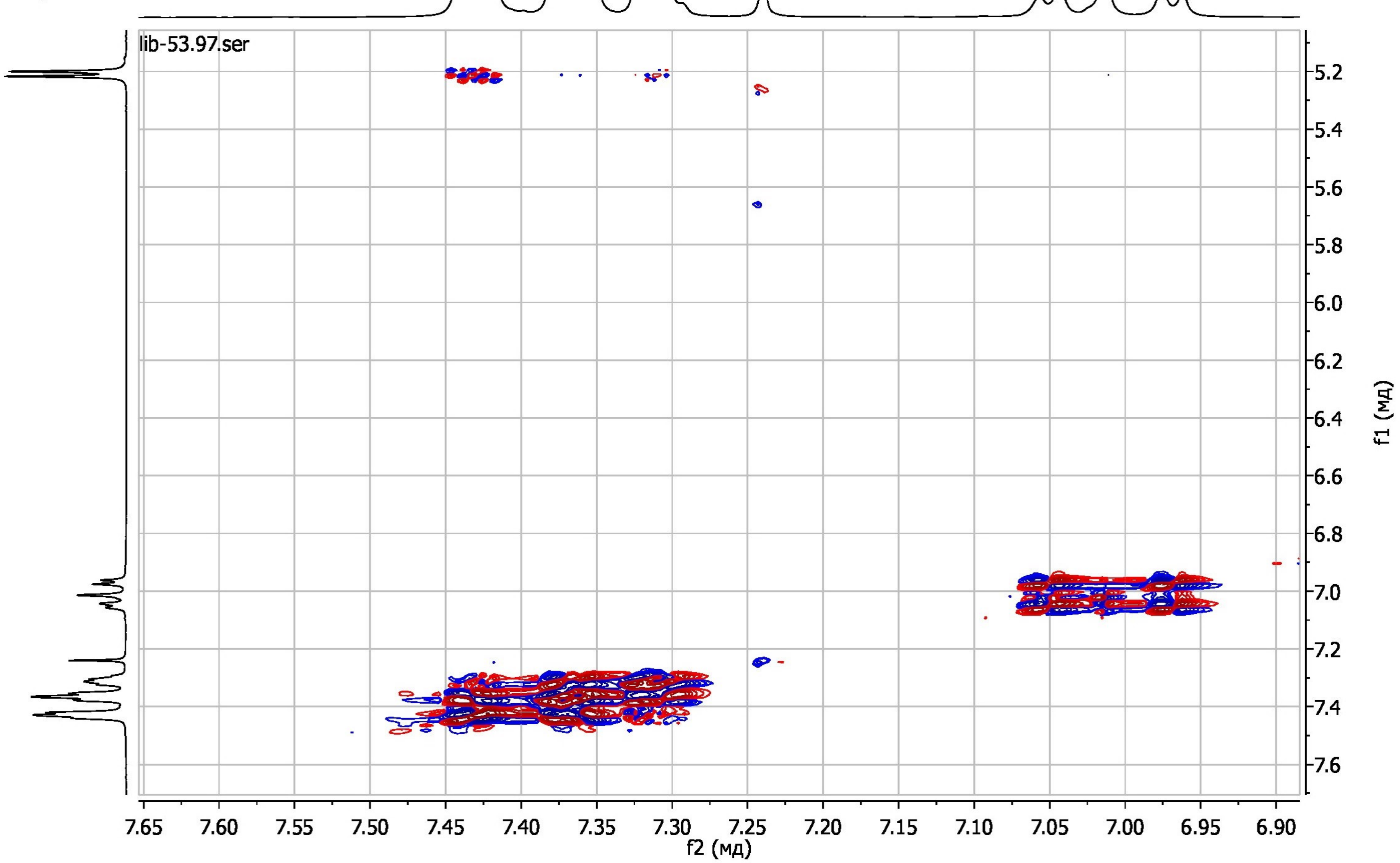

lib-53.97.ser
5.2
5.4
5.6
5.8
6.0
6.2
6.4
6.6
6.8
7.0
7.2
7.4
7.6
f1 (мд)
7.65
7.60
7.55
7.50
7.45
7.40
7.35
7.30
7.25
7.20
7.15
7.10
7.05
7.00
6.95
6.90
f2 (мд)

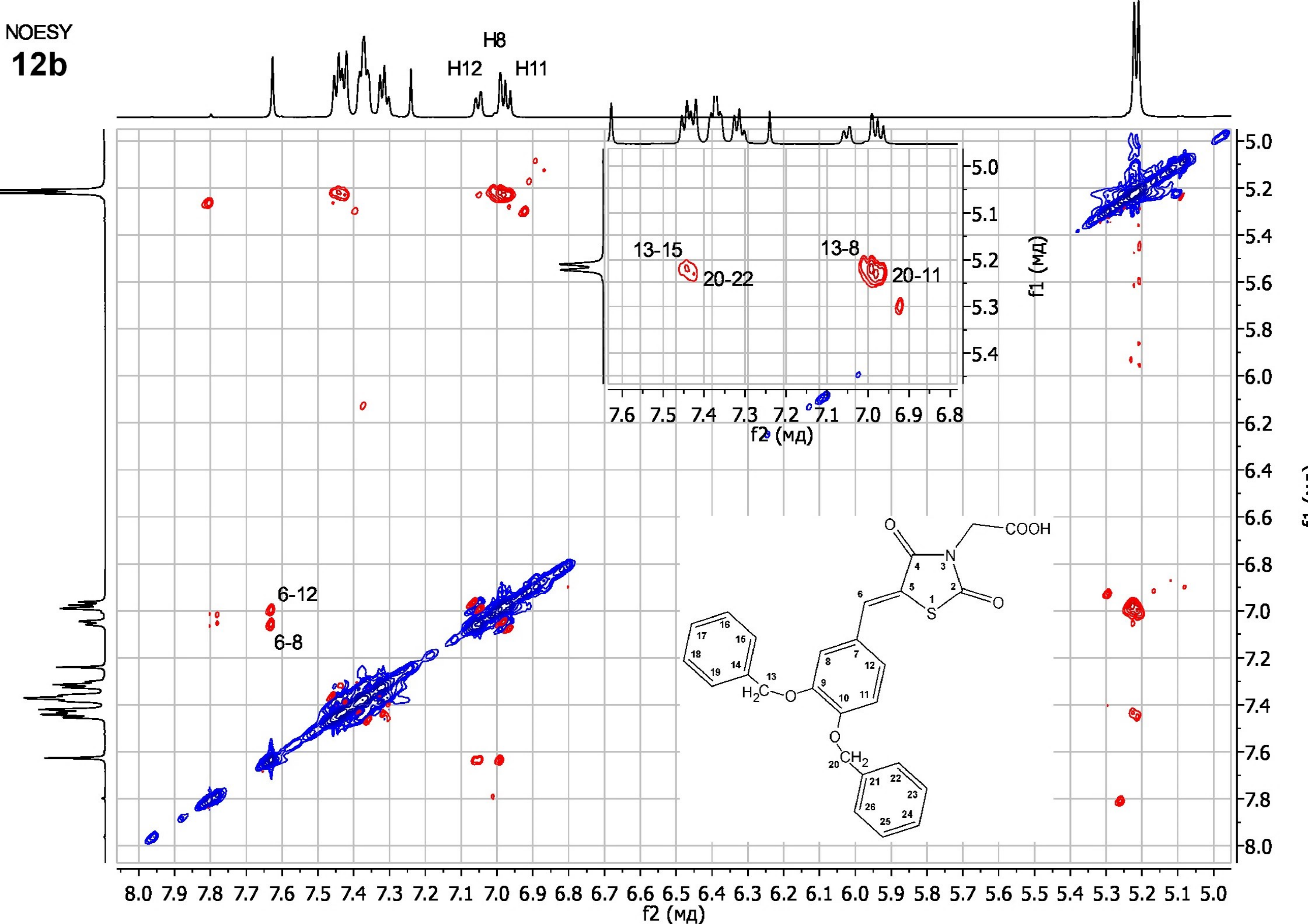
NOESY
12b
H8
H12
H11
13-15
20-22
13-8
20-11
6-12
6-8
f1 (мд)
f2 (мд)
COOH
O
N
S
H_2C
CH_2

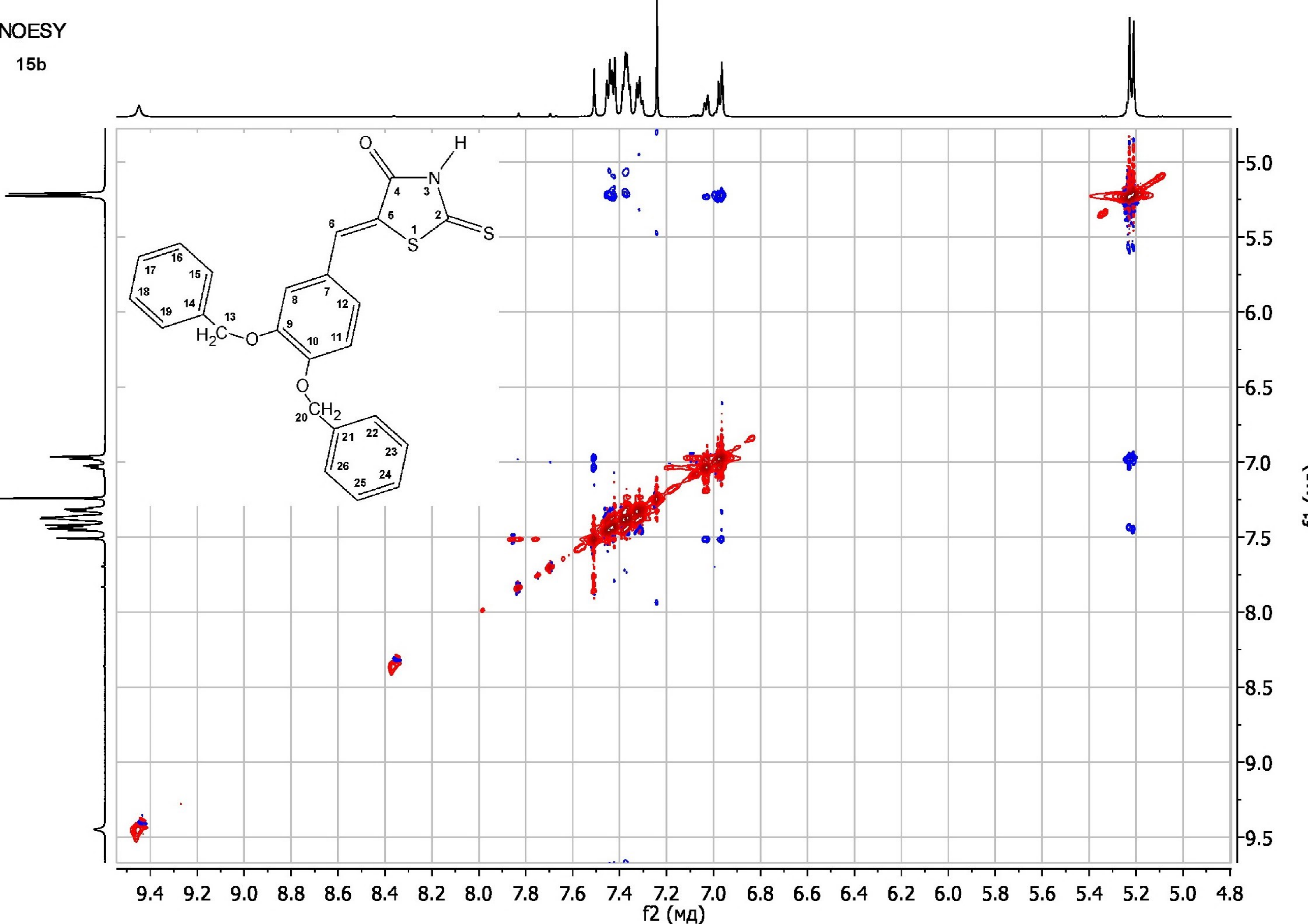
NOESY
15b
f2 (мд)

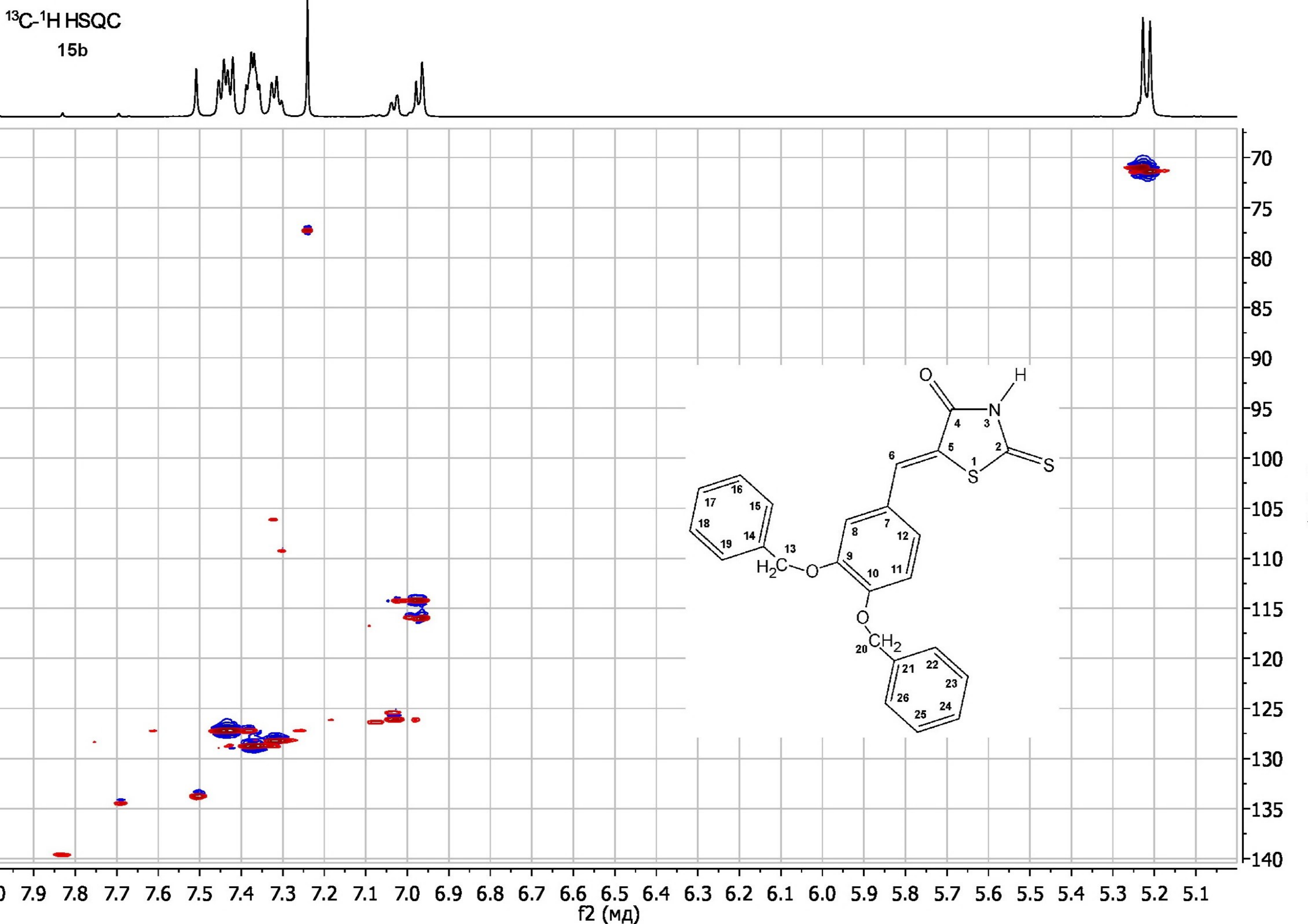

13C-1H HSQC
15b
f2 (мд)

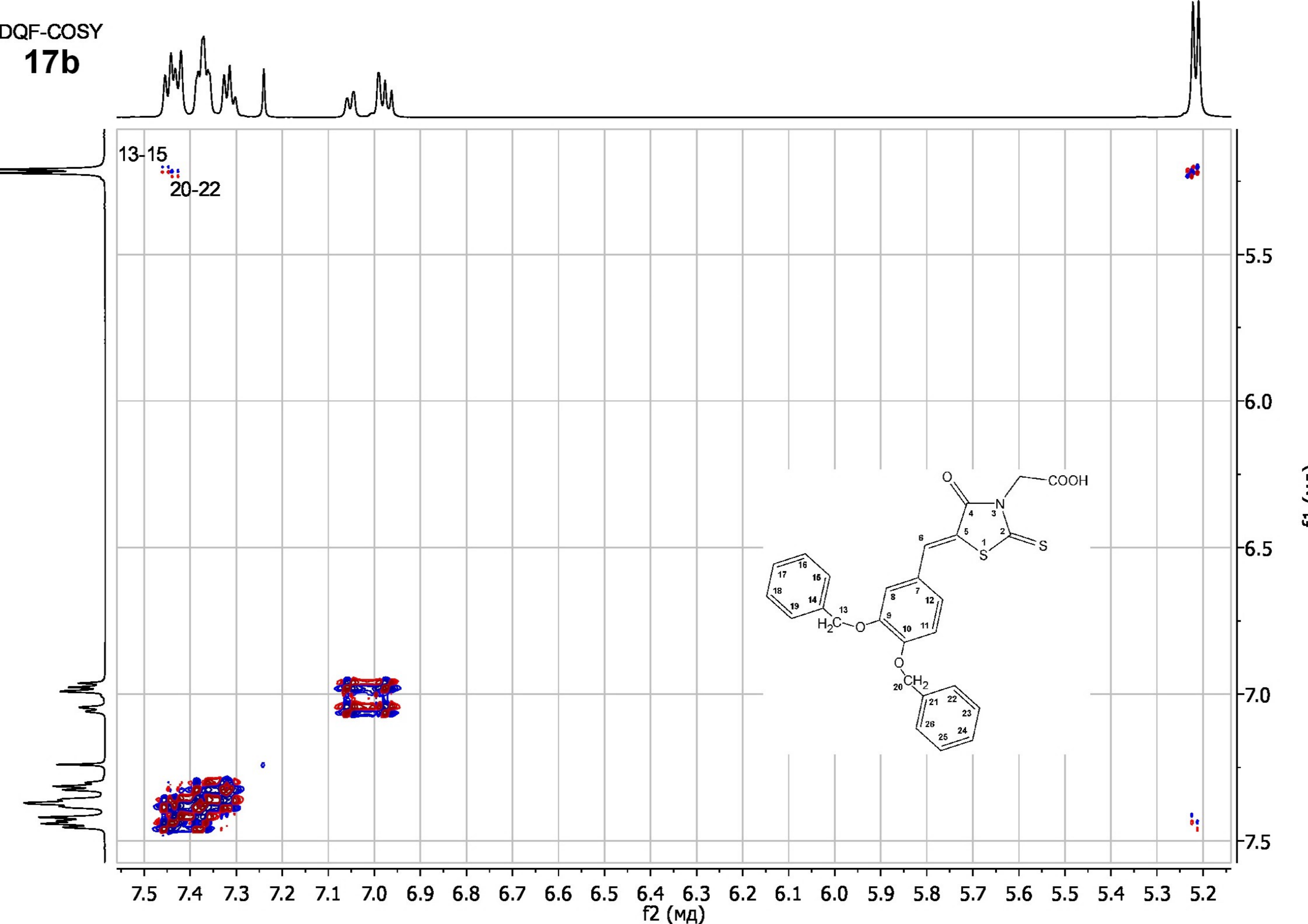
DQF-COSY
17b
13-15
20-22
5.5
6.0
6.5
7.0
7.5
7.5 7.4 7.3 7.2 7.1 7.0 6.9 6.8 6.7 6.6 6.5 6.4 6.3 6.2 6.1 6.0 5.9 5.8 5.7 5.6 5.5 5.4 5.3 5.2
f2 (мд)
O
N
COOH
S
S
H2C
O
O
CH2
1 2 3 4 5 6 7 8 9 10 11 12 13 14 15 16 17 18 19 20 21 22 23 24 25 26

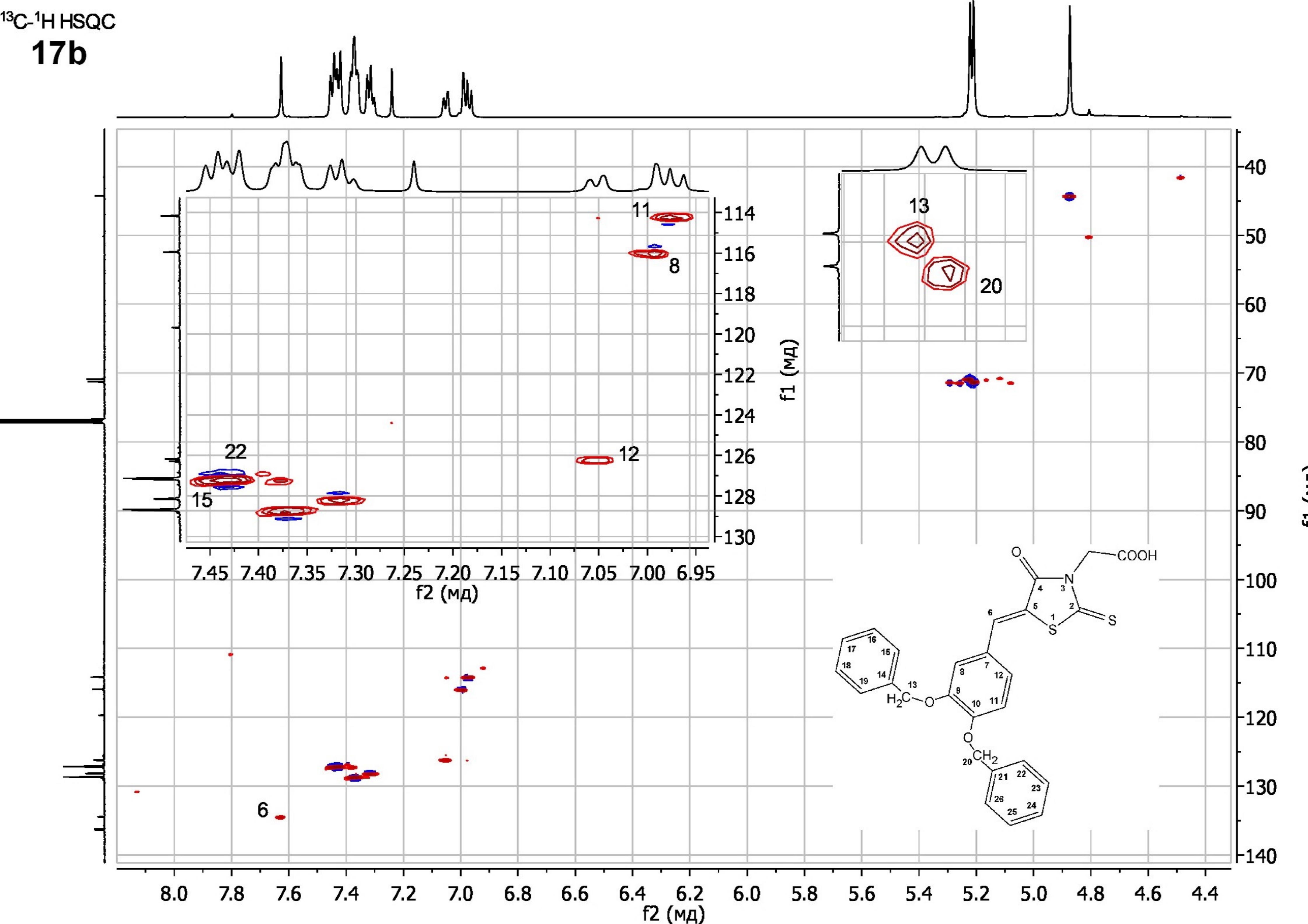
^{13}C-^{1}H HSQC
17b
f2 (мд)
f1 (мд)
8.0 7.8 7.6 7.4 7.2 7.0 6.8 6.6 6.4 6.2 6.0 5.8 5.6 5.4 5.2 5.0 4.8 4.6 4.4
40 50 60 70 80 90 100 110 120 130 140
7.45 7.40 7.35 7.30 7.25 7.20 7.15 7.10 7.05 7.00 6.95
114 116 118 120 122 124 126 128 130
11
8
12
22
15
6
13
20
O
N
COOH
S
H2C
CH2
1 2 3 4 5 6 7 8 9 10 11 12 13 14 15 16 17 18 19 20 21 22 23 24 25 26

DQF-COSY
17b

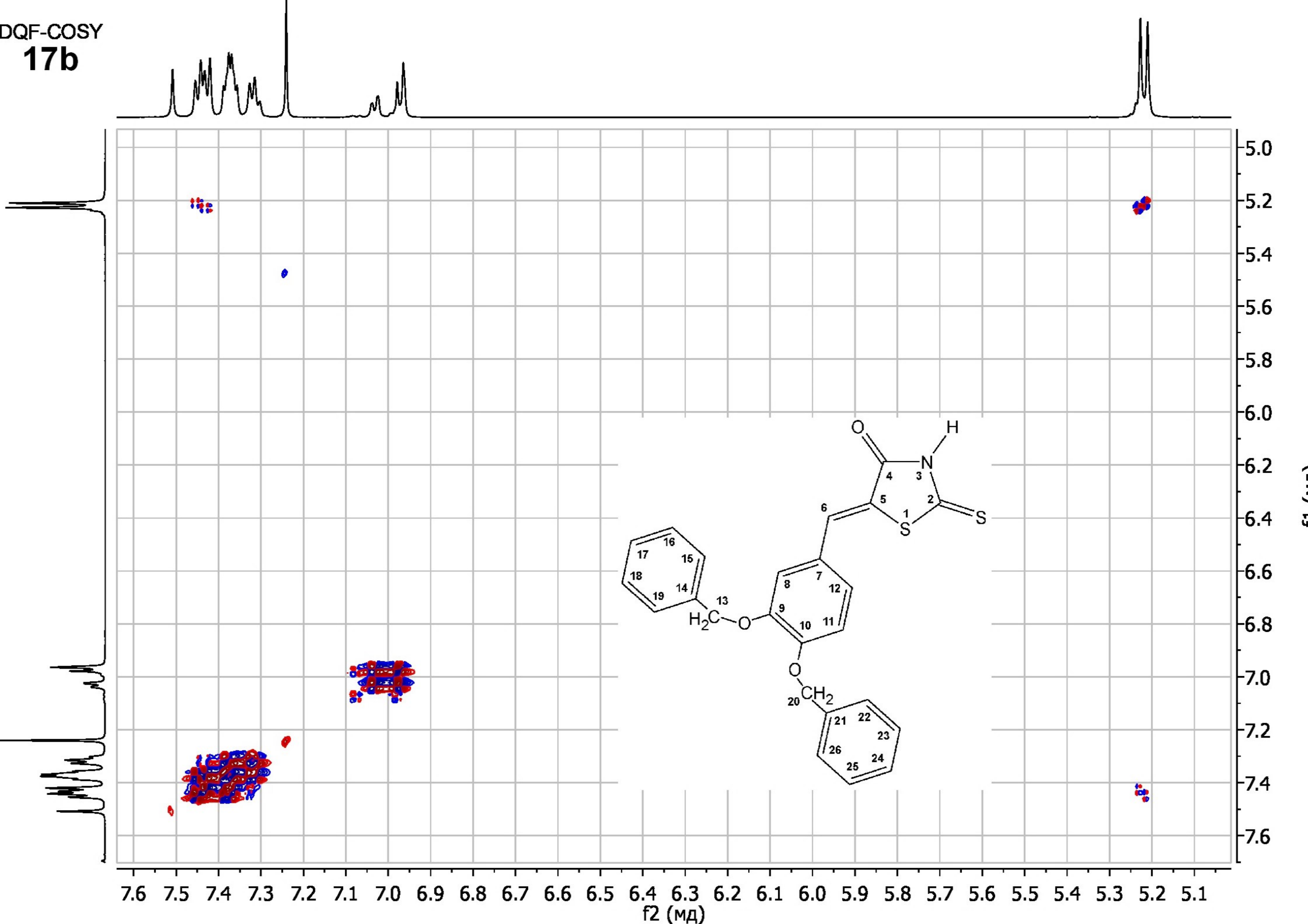

f2 (мд)
f1 (мд)
7.6 7.5 7.4 7.3 7.2 7.1 7.0 6.9 6.8 6.7 6.6 6.5 6.4 6.3 6.2 6.1 6.0 5.9 5.8 5.7 5.6 5.5 5.4 5.3 5.2 5.1
5.0 5.2 5.4 5.6 5.8 6.0 6.2 6.4 6.6 6.8 7.0 7.2 7.4 7.6

NOESY
17b

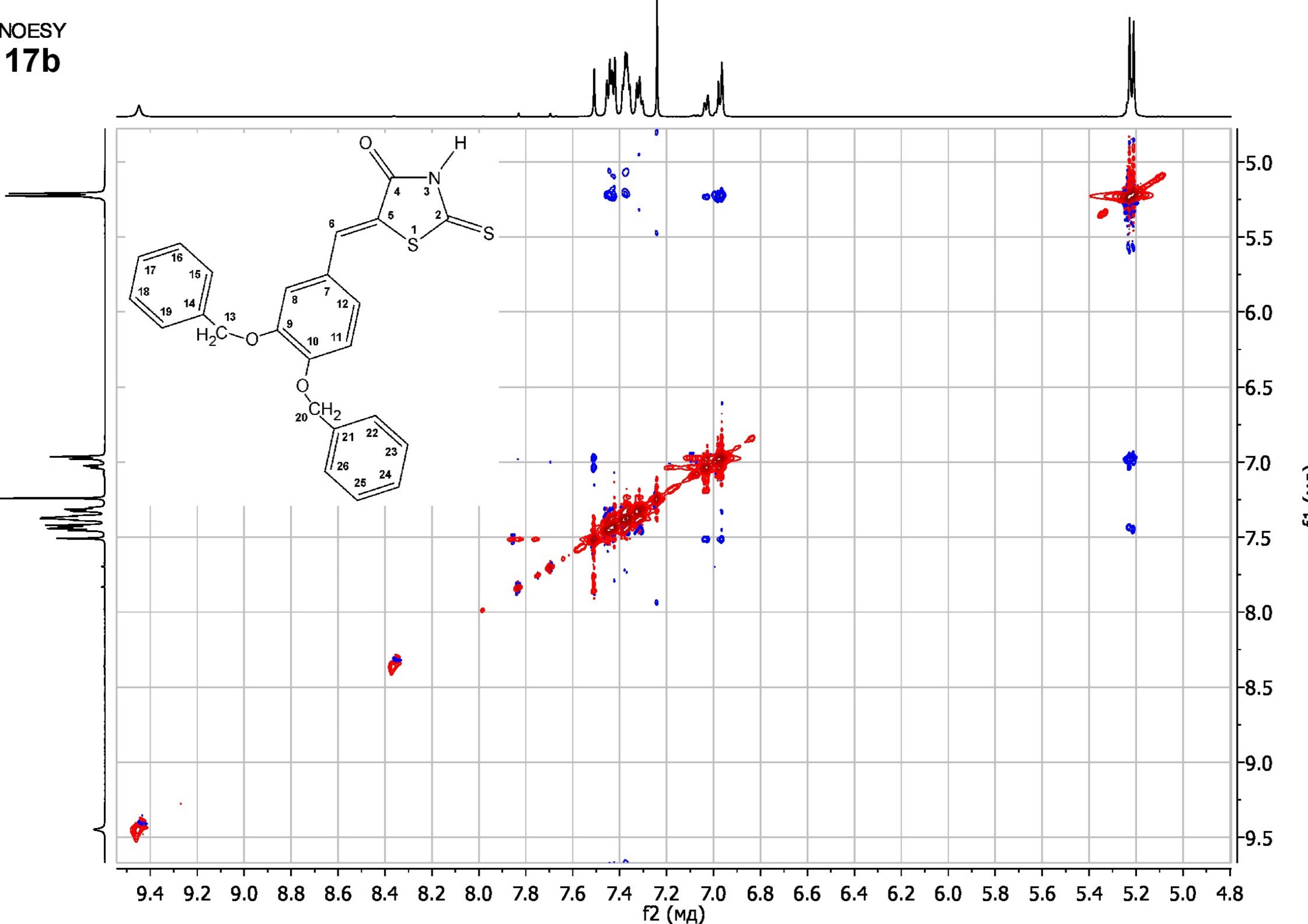
O
H
N
4
3
6
5
2
1
S
S
17
16
15
18
14
19
13
H2C
O
8
7
12
9
10
11
O
20 CH2
21
22
23
26
24
25
9.4 9.2 9.0 8.8 8.6 8.4 8.2 8.0 7.8 7.6 7.4 7.2 7.0 6.8 6.6 6.4 6.2 6.0 5.8 5.6 5.4 5.2 5.0 4.8
f2 (мд)
5.0 5.5 6.0 6.5 7.0 7.5 8.0 8.5 9.0 9.5

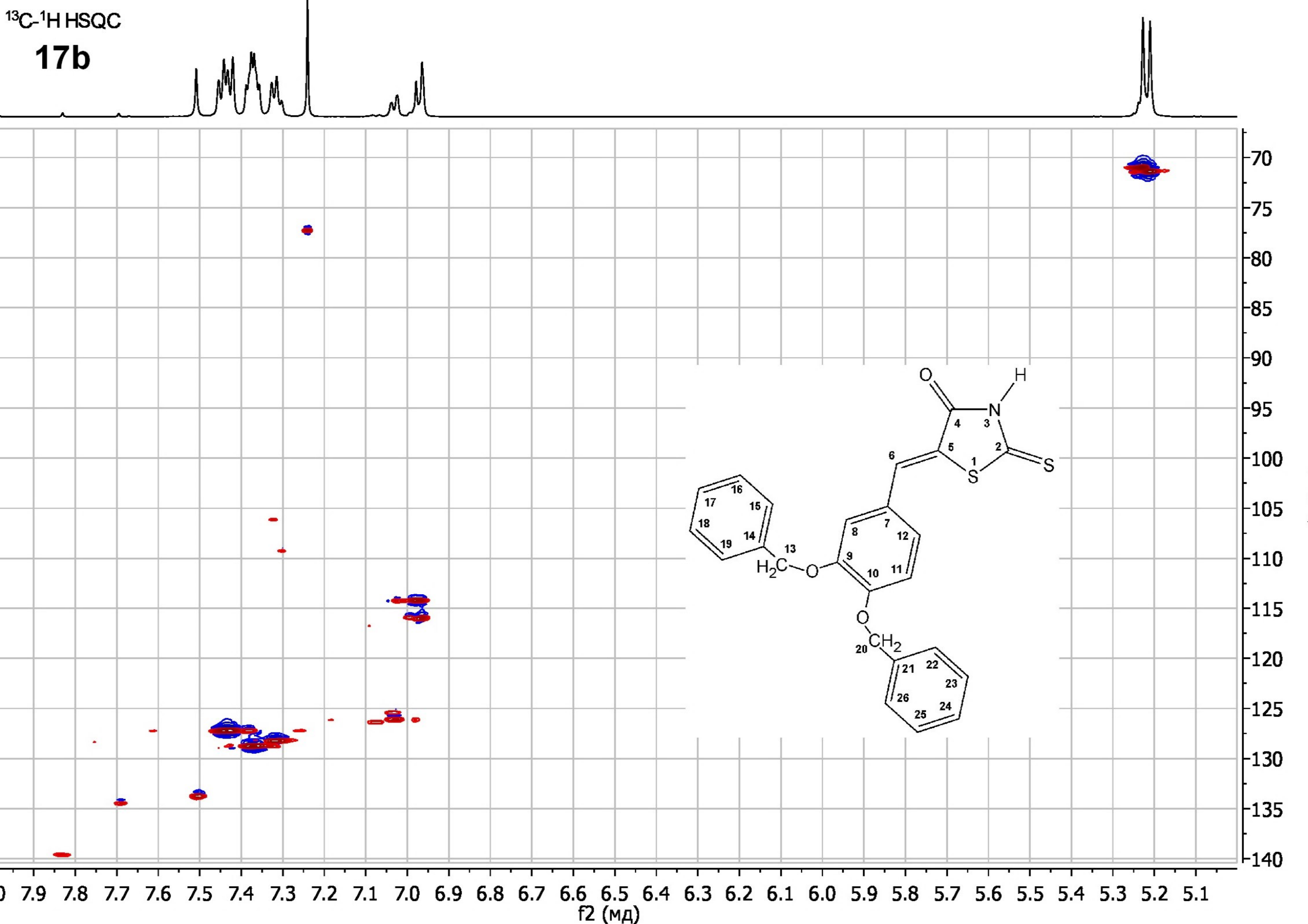

13C-1H HSQC
17b
f2 (мд)
7.9 7.8 7.7 7.6 7.5 7.4 7.3 7.2 7.1 7.0 6.9 6.8 6.7 6.6 6.5 6.4 6.3 6.2 6.1 6.0 5.9 5.8 5.7 5.6 5.5 5.4 5.3 5.2 5.1
70 75 80 85 90 95 100 105 110 115 120 125 130 135 140
O
H
N
S
S
H2C
O
O
CH2
