# Supplementary material for: Antifungal Thiazolidines: Synthesis and Biological Evaluation of Mycosidine Congeners
Source: Pharmaceuticals (Basel). 2022 May 1;15(5):563. doi: 10.3390/ph15050563 (PMC9145892; doi:10.3390/ph15050563)
Supplement: Supplementary file 1 [file pharmaceuticals-15-00563-s001.zip › Supplement_3_figures.pdf]

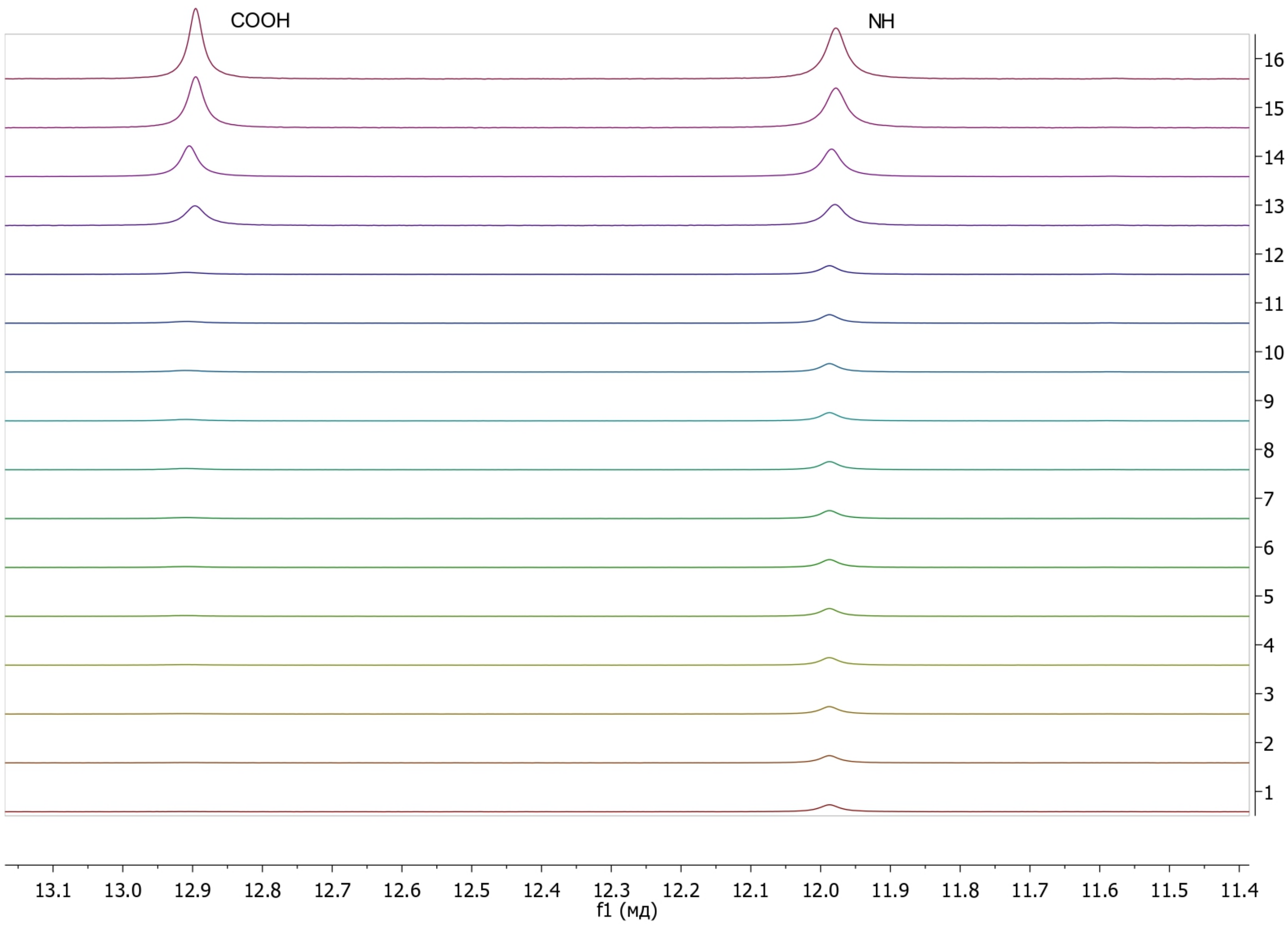

Figure S1. Fragments of ^{1}H NMR spectra of solution of **6a** in DMSO-d_6, recorded at 25°C and 600 MHz ^{1}H frequency at various time intervals after sample dissolution. Labeled are representative signals of initial compound and product of hydrolysis.

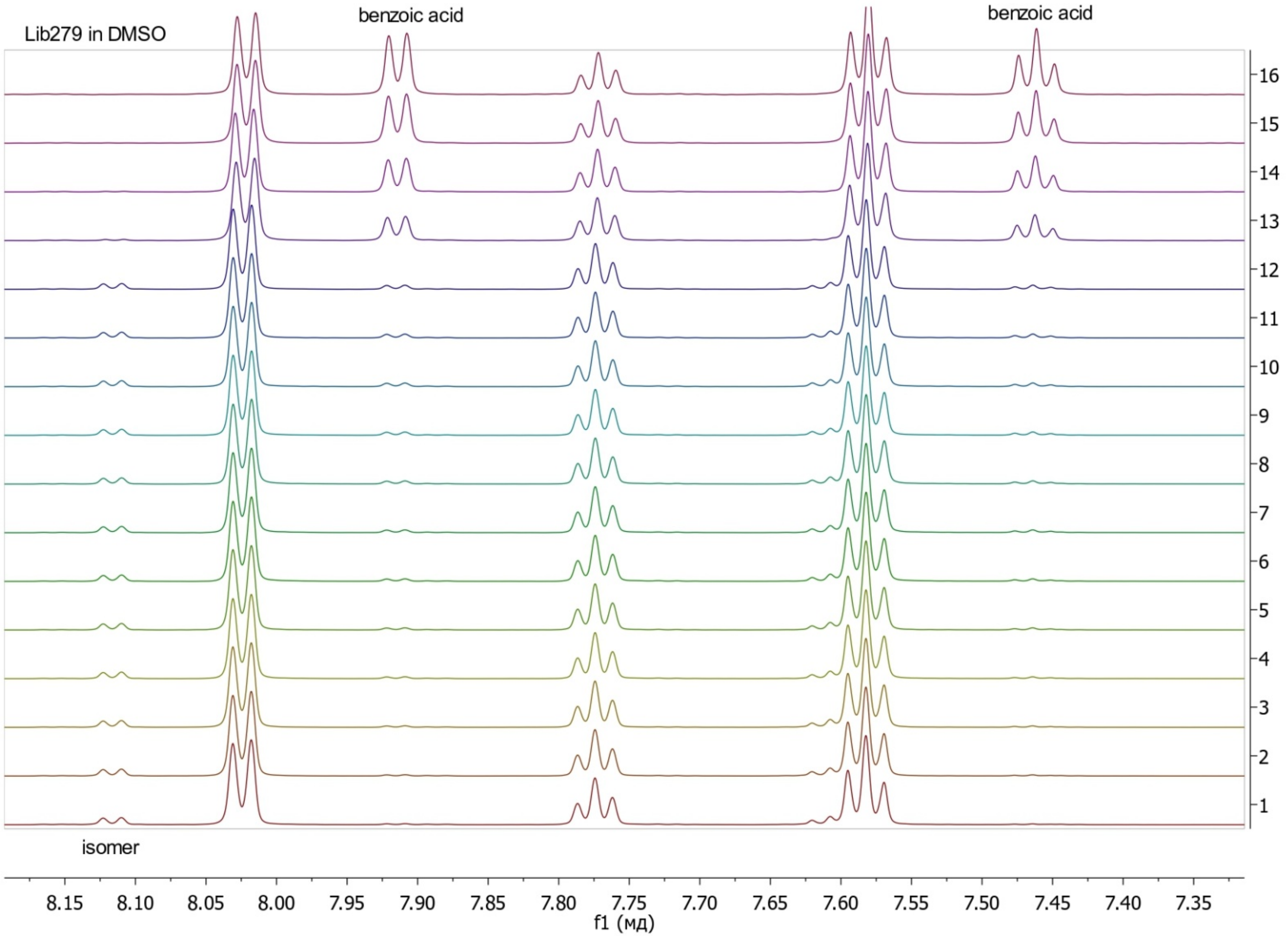

Figure S1 (contnued).

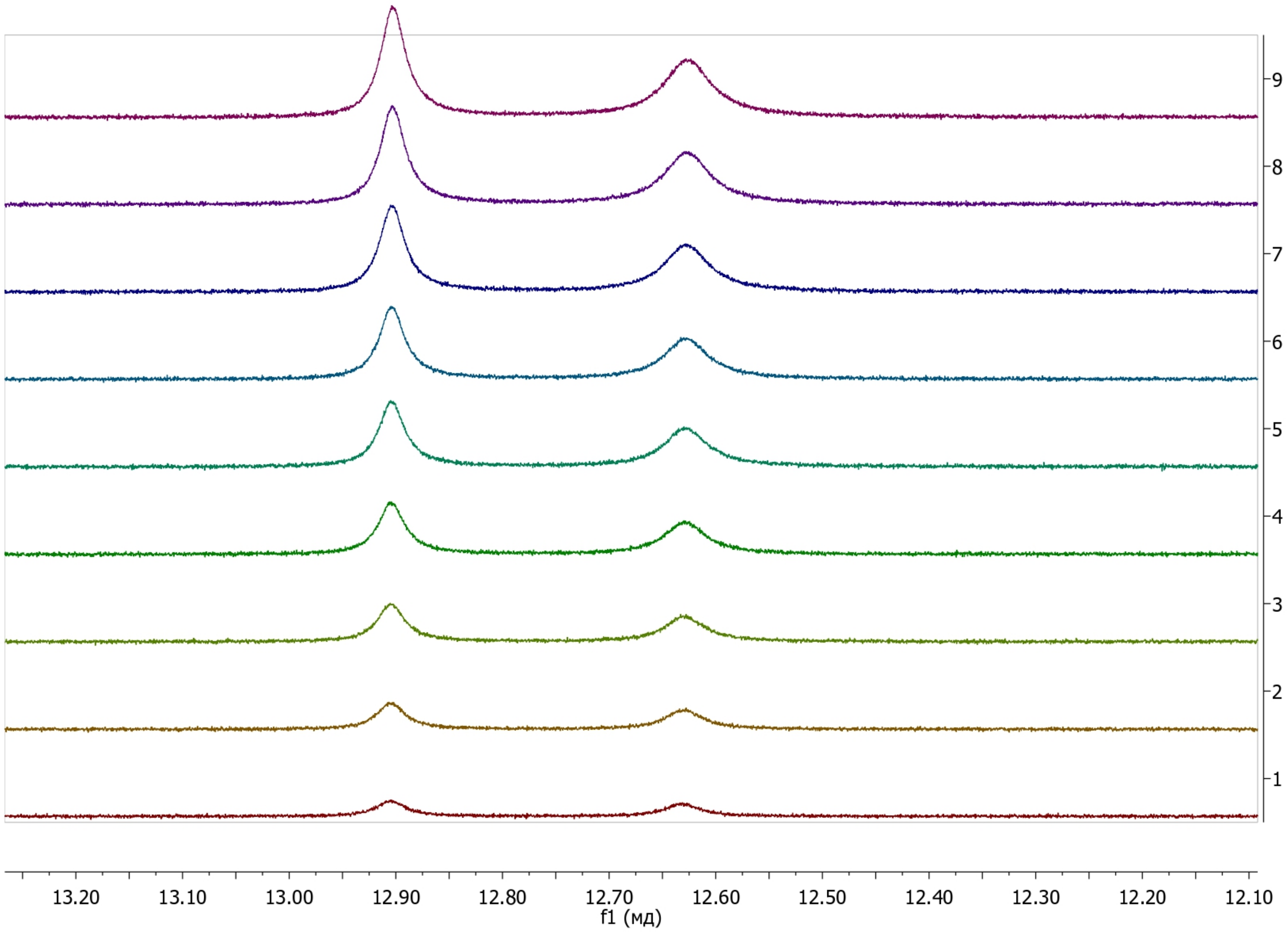

Figure S2. Fragments of ^{1}H NMR spectra of a solution of **7a** in DMSO-d_6, recorded at 25°C at 600 MHz ^{1}H frequency at various time intervals after sample dissolution. Labeled are representative signals of initial compound and product of hydrolysis.

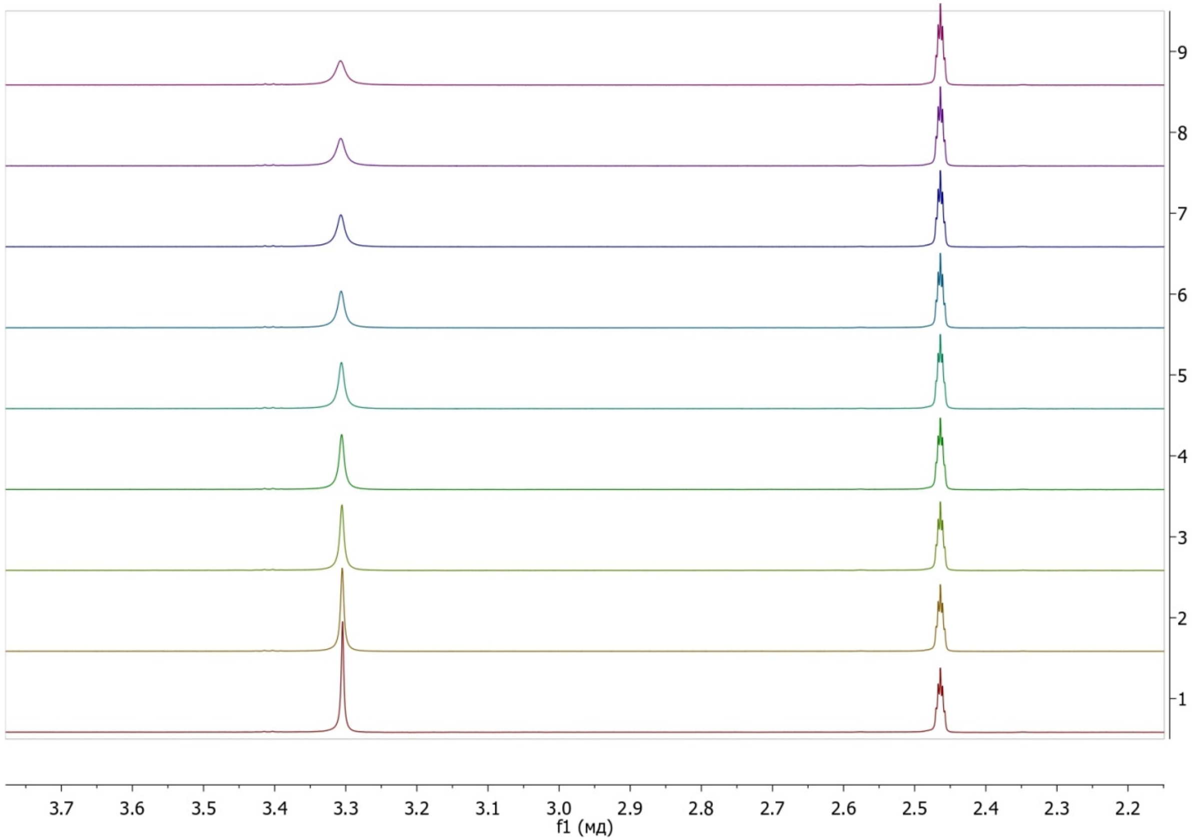

Figure S2 (continued)

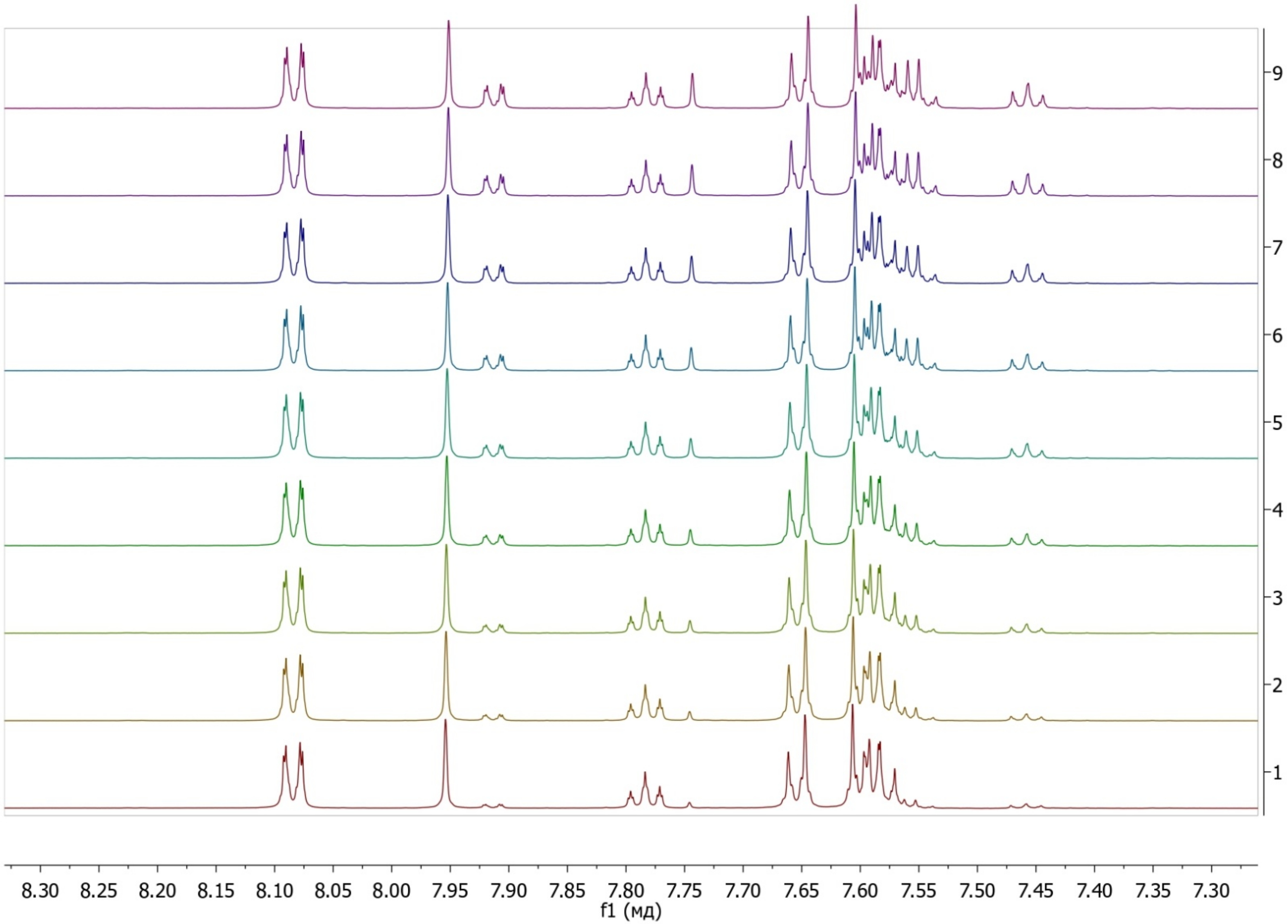

Figure S2 (continued).

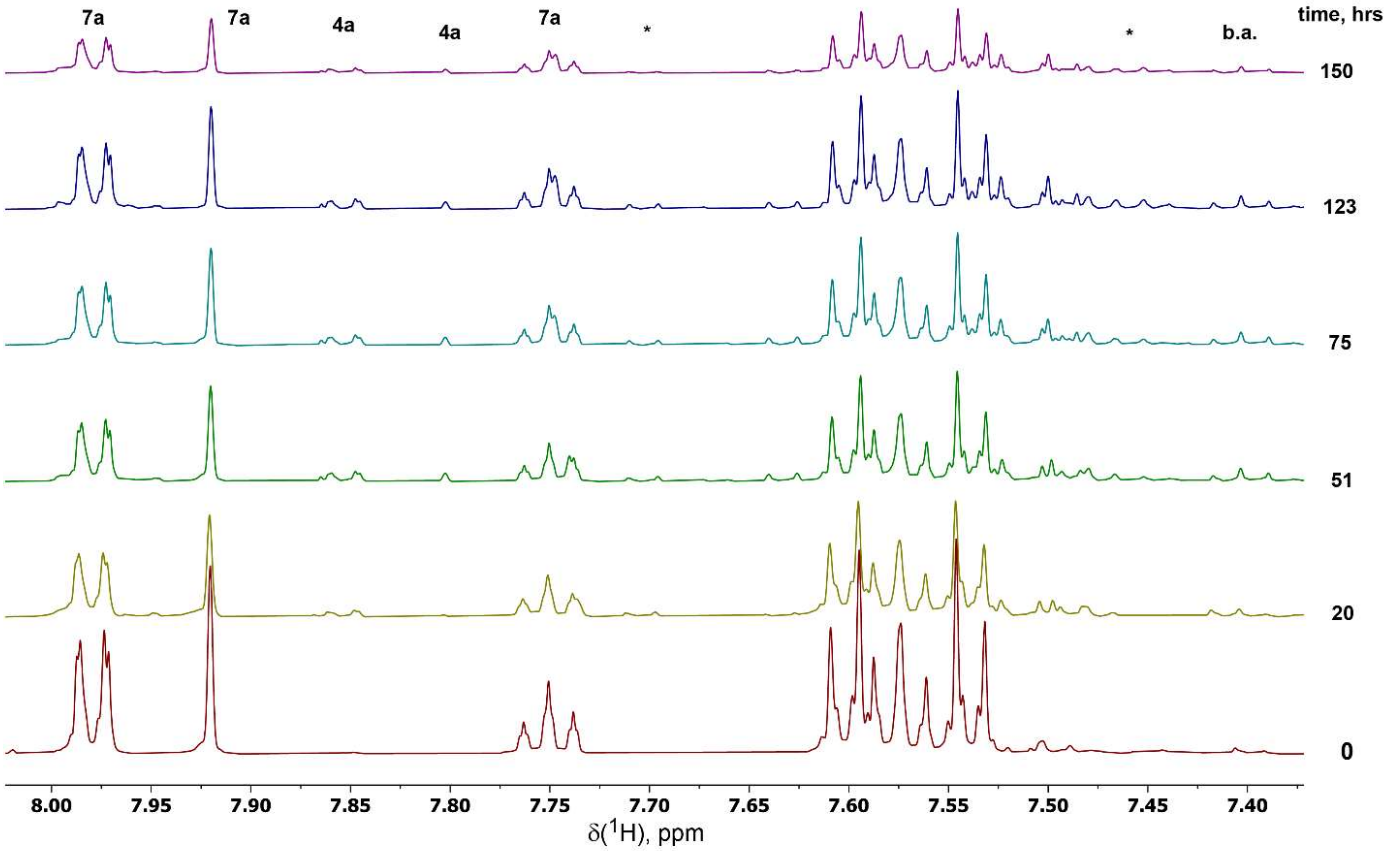

Figure S3. Fragments of ^{1}H NMR spectra of a solution of **7a** in CD_3OD, recorded at 25°C at 600 MHz ^{1}H frequency at various time intervals after sample dissolution. Labeled are representative signals of initial compound and product of hydrolysis. An asterisk marks the signals of the reaction products of **7a** with methanol.

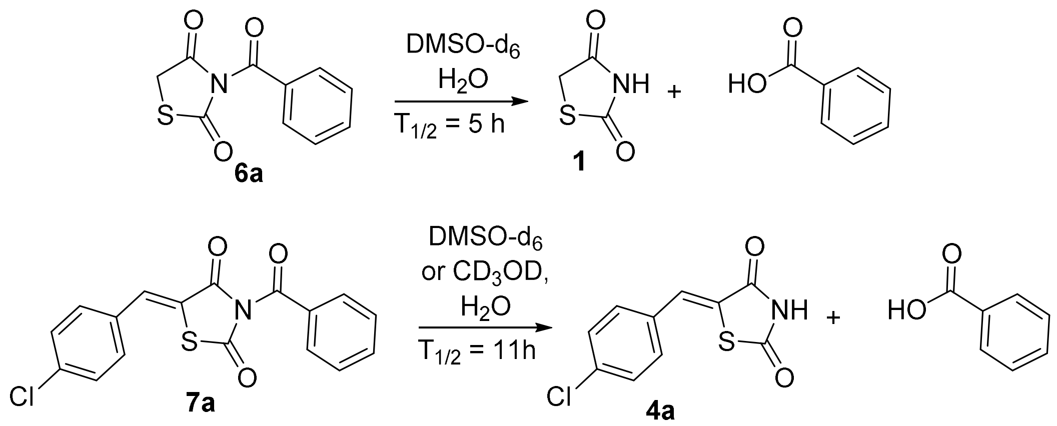

Figure S4. Hydrolysis of **6a** and **7a** to **1**,**4a** and benzoic acid in *DMSO-d_6* and CD_3OD
